# Analyzing Personalized Policies for Online Biometric Verification

**DOI:** 10.1371/journal.pone.0094087

**Published:** 2014-05-01

**Authors:** Apaar Sadhwani, Yan Yang, Lawrence M. Wein

**Affiliations:** 1 Management Science and Engineering Department, Stanford University, Stanford, California, United States of America; 2 Institute for Computational and Mathematical Engineering, Stanford University, Stanford, California, United States of America; 3 Graduate School of Business, Stanford University, Stanford, California, United States of America; University of Catania, Italy

## Abstract

Motivated by India’s nationwide biometric program for social inclusion, we analyze verification (i.e., one-to-one matching) in the case where we possess similarity scores for 10 fingerprints and two irises between a resident’s biometric images at enrollment and his biometric images during his first verification. At subsequent verifications, we allow individualized strategies based on these 12 scores: we acquire a subset of the 12 images, get new scores for this subset that quantify the similarity to the corresponding enrollment images, and use the likelihood ratio (i.e., the likelihood of observing these scores if the resident is genuine divided by the corresponding likelihood if the resident is an imposter) to decide whether a resident is genuine or an imposter. We also consider two-stage policies, where additional images are acquired in a second stage if the first-stage results are inconclusive. Using performance data from India’s program, we develop a new probabilistic model for the joint distribution of the 12 similarity scores and find near-optimal individualized strategies that minimize the false reject rate (FRR) subject to constraints on the false accept rate (FAR) and mean verification delay for each resident. Our individualized policies achieve the same FRR as a policy that acquires (and optimally fuses) 12 biometrics for each resident, which represents a five (four, respectively) log reduction in FRR relative to fingerprint (iris, respectively) policies previously proposed for India’s biometric program. The mean delay is 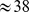 sec for our proposed policy, compared to 30 sec for a policy that acquires one fingerprint and 107 sec for a policy that acquires all 12 biometrics. This policy acquires iris scans from 32–41% of residents (depending on the FAR) and acquires an average of 1.3 fingerprints per resident.

## Introduction

In India, one of the biggest barriers for poor people to access government services is the inability to prove one’s identity [1]. To improve social inclusion [2], the government of India has undertaken the largest biometric program in human history, called the Unique Identification Authority of India (UIDAI), with the aim of creating a unique biometric identity for each of its 1.2 B residents [1]; other countries, such as Indonesia, are developing similar programs [3]. This program requires two main biometric matching activities. During enrollment, it captures 10 fingerprint images and 2 iris images from every resident (as of August 2012, 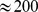
M residents have been enrolled [4]) and performs identification (i.e., 1-to-n matching) to make sure that people do not create multiple identities. Once the system is operational, residents will undergo verification (i.e., 1-to-1 matching) every time they access services, to ensure that they are who they claim to be; this is achieved by capturing new biometrics and comparing them to their corresponding biometrics from enrollment. UIDAI predicts that it will perform up to 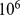 verifications/hr after the system is operational, and that most of these verifications will be online, i.e., performed while the resident waits.

Details of the verification approach (e.g., whether to use fingerprints and/or irises) may be left to local or regional governments and may depend on the nature of the application; e.g., receiving money may require a more stringent process than receiving other services. UIDAI has carried out extensive verification experiments with fingerprints [5] and irises [4], and (as of October 2013) have implemented a policy that is a variant of a policy considered in [5]. Hence, there is a pressing need to identify policies that are more accurate than those in [4] and [5], but do not cause too much delay for residents. A key complicating feature of the verification problem is that different hardware and different procedures are used during enrollment and verification. More specifically, enrollment uses more sophisticated hardware and a more standardized procedure (e.g., with human guidance to guarantee the best possible images) than verification. Similarity scores when both images are generated by the same equipment can differ considerably from scores when the images are generated by different equipment. Moreover, information gathered at enrollment, such as fingerprint image quality, may be of limited value in predicting the similarity scores during verification (in contrast, when the same equipment is used at enrollment and at subsequent identification, i.e. one-to-many matching, or verification, then image quality can be helpful, e.g., Wein and Baveja [6]). As a result, UIDAI developed the idea of Best Finger Detection (BFD), which would occur during a resident’s first verification (delaying BFD until the first verification has the added benefit of reducing the possibility of an accidental error or a successful intrusion). During BFD, all 10 fingers are recaptured and the similarity score between the new images and the enrollment images are used as a basis for determining the best finger. They found that using everyone’s best finger performs much better than using everyone’s right thumb [5], which was the policy that was under initial consideration. Moreover, they found that using everyone’s two best fingers performs even better [5], and to reduce delay, they have implemented a two-stage policy, where everyone’s best finger is acquired in stage 1, and for residents whose similarity score falls below a specified threshold, their second-best finger is acquired in stage 2. Another important consequence of UIDAI’s use of different equipment and procedures during enrollment and verification is that the plethora of publicly available biometric data, e.g., on the National Institute of Standard and Technology’s (NIST) web site, which contains similarity scores from images that use the same equipment, are not relevant for our purposes. Hence, because UIDAI has not made their raw data publicly available, we need to resort to performance data published by UIDAI [4], [5].

Here, we take the BFD idea a step further by (i) formulating a new mathematical model for fingerprint and iris matching that captures interperson variability and intraperson interfinger (or inter-iris) variability in similarity scores, as well as measurement noise during image capture at verification, (ii) estimating the parameters of this model using extensive experiments performed by UIDAI, (iii) introducing a Best Iris Detection (BID) process that is analogous to BFD, (iv) finding near-optimal single-stage and two-stage individualized policies that allow for a varying number of fingers or irises to be used, depending upon their similarity scores during the BFD and BID processes, and (v) comparing the performance of these optimized policies to several policies considered in [4]–[5].

## Materials and Methods

### UIDAI System

During enrollment, 10 fingerprint images (using a 4-4-2 slap, where the four non-thumbs from each hand are taken from one hand and then the other hand, followed by the two thumbs) and two iris images are obtained from each resident with the help of a human operator. More specifically, they take up to five attempts of each slap if any finger in the slap has an image quality of 4 or 5 on NIST’s 5-point image quality scale (where quality 1 is best and 5 is worst) [7], and they use the best outcome from each slap.

Because dual-eye cameras are more accurate and cause less delay than single-eye cameras [4], we restrict ourselves to dual-eye cameras. That is, anytime iris scanning occurs, both irises are scanned. The same iris scanning process is used during enrollment and verification: they keep the first image that meets the quality threshold or the best among three images if none meet the threshold.

After the UIDAI system becomes fully operational, residents will undergo verification each time they use the system (e.g., to access government services). The verification process uses different hardware (e.g., a smaller single-finger sensor) than the enrollment process, and a finger-placement procedure that does not require a human operator to be present. Iris capture during verification would still require a human operator. The current pilot projects for verification adopt a fixed-finger approach, which uses every resident’s right thumb (and no irises). However, because the BFD approach performs much better than the fixed-finger approach in experiments [5], we incorporate the BFD process into our model. BFD occurs during a resident’s first verification: the system obtains a new set of 10 fingerprint images (one finger at a time), and makes up to three attempts in total on each finger or until each finger has NIST image quality 1 or 2 [8]. After obtaining these new images, the UIDAI system computes the 10 similarity scores between the images during BFD and the corresponding images during enrollment, and normalizes these scores to be in the range from 0 to 100. They then assign the color green if a score is 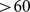, yellow if the score is between 20 and 60, and red if the score is 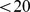. UIDAI has a fixed prioritization of the fingers (from best to worst: right thumb, left thumb, right index, left index, right middle, left middle, right ring, left ring, right little, left little). The final BFD ranking of the 10 fingers depends on the color and the fixed priority: green fingers are ranked higher than yellow fingers, which are ranked higher than red fingers, but the rankings within color are according to their fixed priority. This individualized BFD ranking remains fixed for each resident during subsequent verifications.

### Model Overview

In contrast to the current UIDAI system, our model incorporates a corresponding BID process that occurs during a resident’s first verification, where new iris scans are acquired and similarity scores between the new scans and those from enrollment are computed. We develop a probabilistic model for each resident’s 12 genuine (i.e., a comparison of their new images and those captured during enrollment) similarity scores obtained during the BFD and BID processes, and also each resident’s similarity scores during subsequent verifications. This model captures interperson variability (e.g., some people have more defined fingerprint or iris features than others), intraperson interfinger (or inter-iris) variability (e.g., for any given person, some fingerprints have more defined features than others and some fingers, such as the right thumb, have higher intraperson similarity scores on average than other fingers, such as the left little finger), and measurement noise during the BFD and BID processes and all subsequent verifications.

We also construct several classes of individualized verification policies, which decide on a subset of the 10 fingers and 2 irises to use for verification based on the values of a resident’s 12 similarity scores during BFD and BID. During each verification, new similarity scores are computed between the subset of new images and the corresponding images during enrollment, and then a decision is made based on these new scores. For single-stage policies, there are two options in this decision: either accept (i.e., decide that the resident is indeed who he claims to be) or reject (i.e., decide that the resident is an imposter). In two-stage policies, there is a third option of continuing to a second stage, where additional fingerprint and/or iris images are obtained, followed by an accept/reject decision.

We also develop a probabilistic model for imposter similarity scores, which is the similarity score between fingerprints or irises of different individuals. We optimize over our policy classes to minimize the false reject rate (FRR), which is the probability that we reject a resident who is genuine, subject to constraints on the false accept rate (FAR), which is the probability that we accept a resident who is an imposter, and the average amount of time it takes to verify a resident. We compare the optimized classes of policies to several policies considered in [5].

### Biometric Model

Let 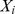 be the true (as opposed to measured) genuine similarity score between the enrollment image and the image during verification (including during BFD and BID), where 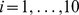 corresponds to the standard indexing scheme for fingerprints (left little, left ring, left middle, left index, left thumb, right thumb, right index, right middle, right ring, right little), 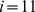 is the left iris and 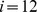 is the right iris. We assume that fingerprint similarity scores are independent of iris similarity scores.

For fingerprints, each person has an overall image quality 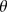, which is not to be confused with NIST’s 5-point image quality. We assume that 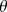 is a normal random variable with mean 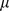 and variance 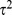 (i.e., 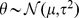). Given a person’s realization of the random variable 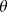, we assume that the true log (tilde’s are used to denote logarithmic quantities) similarity score 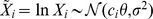, where the 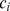
’s are normalized so that 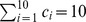. Hence, 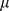 is the overall mean genuine log similarity score, 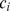 is the finger-dependent correction, 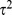 is the interperson variance, 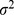 is the intraperson interfinger variance, and a resident’s true similarity score (given 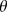) is lognormally distributed, which generates positive similarity scores and provides the flexibility to model a variety of empirical distributions (e.g., [6]).

Because we do not have raw similarity score data, we finesse some of the details in the measurement process described earlier, such as the color-coding scheme and the conditional number of attempts made during BFD. Let 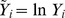 be the log similarity score for finger 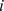 obtained during BFD. We assume that 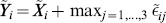 for 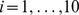, where 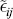 is the measurement noise associated with the 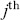 attempt for finger 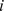 during BFD. We assume that 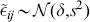 and independent and identically distributed (iid) for all 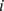 and 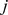; we anticipate that 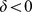 to capture the fact that measurement noise typically acts to reduce genuine similarity scores because of improper finger placement or dirty fingers. Hence, we assume that three attempts are always made during BFD, and we ignore the color coding scheme. During subsequent verifications (with a single acquisition attempt), we assume that the log similarity score for each attempt of finger 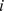 is 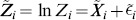, where 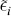 is also 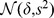 and independent of 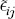.

We assume that the imposter similarity score, which measures the similarity between finger 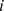 of one person and finger 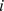 of a different person, has a lognormal distribution (with parameters 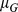 and 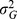) that is independent of the finger type 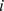 and of image quality. Moreover, due to insufficient data, we ignore measurement noise in the interperson similarity scores, so that repeat measurements generate the same score.

We also develop a probabilistic model for each resident’s two iris similarity scores during each verification. Unlike fingerprints, the same iris capture process is used during the first verification (i.e., the BID process) and in all subsequent verifications. Our model for genuine similarity scores for fingerprints offers a succinct way to capture interperson and intraperson variability for 10 fingers. With only two irises, we can capture both of these issues by simply having correlated similarity scores between left and right irises. Although Hamming distances are often used to compare two irises, similarity scores (roughly on a 0–100 scale) are used in the experiments in [4], which maintains consistency with the fingerprint model. We model the true genuine similarity scores of two irises, denoted by 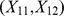, by a symmetric bivariate lognormal distribution, where 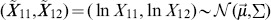, 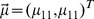 and 
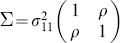
. The genuine similarity score, 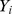, measured during the first verification satisfies 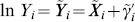, where 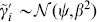 is the log measurement noise. The genuine similarity score, 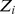, measured during subsequent verifications is given by 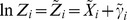, where 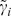 is also 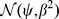 and independent of 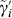.

The imposter Hamming distance is accurately modeled by a distribution that is the maximum (among several rotations) of several binomial random variables [9]. However, for analytical tractability, we assume that the imposter distribution for each iris is iid lognormal with parameters 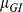 and 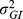. As with fingerprints, we ignore measurement noise in the imposter iris scores.

### Biometric Parameter Estimation

We estimate the fingerprint parameters 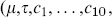 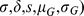 from 61 probabilities that appear in Figs. 8, 10 and 11 in [5]; see 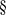
1 in File S1 for details. These studies use 3500 residents based on Wayman’s “Rule of 30” [10], so as to obtain true FRRs that are within 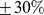 of the observed error rates, and use a large number of imposter scores from the field to obtain statistically significant FAR results in the range from 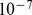 to 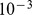 [5]. We use a two-stage estimation process because the experimental set-up in Fig. 8 of [5] differs from that in Figs. 10–11 of [5]: the former uses one very good sensor and includes the 1.87% of people that were unlikely to be verified successfully because they had red rank-1 and rank-2 fingers in UIDAI’s color-coding scheme, while the latter uses the average of 14 good sensors and excludes the 1.87% of people with insufficient image quality. Fig. 8 of [5] contains the probabilities that each of the 10 fingers is the rank-1 finger and the rank-2 finger during the BFD process. We calculate mathematical expressions for these 20 probabilities in terms of the model parameters and choose 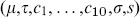 to minimize the sum of squared deviations between the observed and predicted probabilities. We retain only 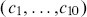 from this solution.

In the second stage, we first use four known threshold values that generate four FAR values in the one-finger setting [5] to estimate the imposter parameters 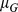 and 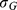. We then use three FRR vs. FAR curves – each consisting of seven points – from Figs. 10–11 in [5] to estimate the remaining parameters. More specifically, we use the blue curve in Fig. 10 of [5], which performs verification using a single attempt of the rank-1 finger during BFD, the red curve in Fig. 10 of [5], which uses up to three attempts of the rank-1 finger, and the green curve in Fig. 11 of [5], which uses the sum of the rank-1 and rank-2 fingers during BFD with up to three attempts. After deriving mathematical expressions for FRR and FAR in these three cases, we choose 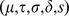 to minimize the sum of squared deviations between the observed and predicted FRR values subject to constraints that the predicted FAR values coincide with the observed values.

Recall that 98.13% of people in the fingerprint studies were likely to be verified successfully using 1 or 2 fingers, and the remaining 1.87% were excluded from the fingerprint verification studies (pg 23–24 of [5]). Because we are allowing up to 10 fingers to be used for verification and because UIDAI’s failure-to-acquire (FTA) rate due to poor biometrics is only 0.14% [11], we estimate the parameter values in two scenarios. In the exclusion scenario, we assume that the 1.87% of people are omitted from the study and use the 21 FRR and FAR values directly. In the inclusion scenario, we assume that the failure-to-acquire (FTA) rate is 0.0187 and that the 21 FRR and FAR values from Figs. 10–11 in [5] are false non-match rates (FNMR) and false match rates (FMR), respectively. We then recalculate the 21 FRR and FAR values via the formulas FRR = FTA+FNMR(1-FTA) and FAR = FMR(1-FTA) (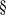
8.3.2.2 and 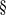
8.3.3.2 of [12]). While the exclusion scenario requires less extrapolation of the data, the inclusion scenario allows us to obtain a rough idea of the potential of our proposed policy when applied to the entire population.

We estimate most of the iris parameters from the dual-eye experiments reported in Fig. 13 and Table 8 in [4], which include four points on a FRR vs. FAR curve for two attempts of both irises, and one (FRR,FAR) point for one attempt of both irises. For multiple attempts, the maximum similarity score is used. In these experiments, the similarity scores for left and right irises are fused using the maximum of the two scores. Of the seven parameters to estimate, 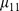 and 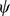 appear in the mathematical expressions for FRR only via their sum 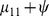. Hence, we cannot determine their individual values and arbitrarily assume that 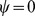, leaving us with six parameters: 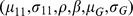. We estimate the imposter parameters 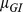 and 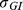 using Hamming distance data in [9] and the assumption that the similarity scores equal 100 times 1 minus the Hamming distance. We estimate the correlation 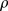 from Fig. 6 of [13], and then choose 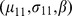 to minimize the sum of five squared deviations between the observed and predicted FRR values subject to constraints that the predicted FAR equals the observed values. The iris FTA rate is 0.0033 [4], and we estimate the iris parameter values in the inclusion and exclusion scenarios exactly as in the fingerprint case: ignoring 0.0033 in the exclusion scenario and using the formulas FRR = FTA+FNMR(1-FTA) and FAR = FMR(1-FTA) in the inclusion scenario.

### Policies

For the purposes of comparison, we consider three benchmark policies that are tested in [4]–[5]. The first benchmark policy is the simplest individualized fingerprint policy, which uses one attempt of the rank-1 finger (as measured by BFD). We also test one attempt of the sum of the rank-1 and rank-2 fingers, which is also considered in [5]. We do not consider the versions of these two policies that use up to three attempts, because there are not ample data to measure the average delay incurred by residents for these policies (i.e., we do not know the mean number of attempts that were actually made); however, results in [5] show that acquiring new biometrics results in better performance than re-acquiring biometrics. The final benchmark policy uses one attempt of the maximum of the left and right iris score [12]. These three benchmark policies have a single parameter, which is the accept/reject threshold (i.e., accept the resident if the similarity score is greater than the threshold).

**Figure 1:**
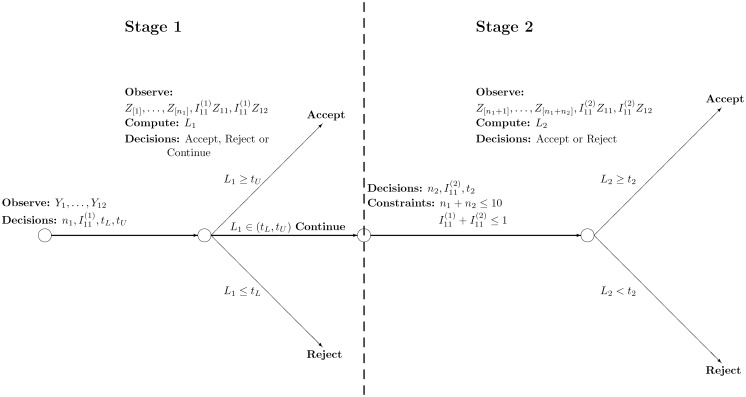
The general two-stage class of policies. In stage 1, for each resident we choose the number of fingers (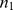) to acquire and whether 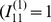) or not (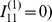 to acquire the irises, based on the BFD and BID scores 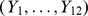. We then observe the new similarity scores 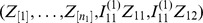 of the acquired biometrics, where the fingerprint scores 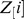 are ranked according to the index 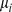. We compute the likelihood ratio 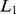 and accept the resident as genuine if 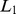 is greater than the upper threshold 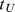, reject the resident if 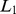 is smaller than the lower threshold 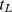, and otherwise continue to stage 2, where both irises (if 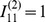) and 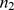 additional fingerprints are acquired. Finally, we compute the likelihood ratio 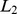 based on the biometrics acquired in stage 2 and then accept or reject the resident using the second-stage threshold 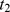.

We optimize six classes of policies that are special cases of the general two-stage policy pictured in Fig. 1. Our approach uses likelihood ratios (Fig. 1), which is known to be optimal (in the Neyman-Pearson sense) for a single-stage problem in the absence of a delay constraint [14]. We show in 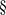
3.1 of File S1 that it is optimal to rank the fingers of each resident by the index 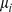, which is defined in terms of the model parameters 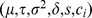 and the similarity scores 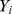 observed during BFD via equations (8), (9), (63) and (65) in File S1. This ranking greatly simplifies the computation of an optimal policy: e.g., in stage 1 we simply need to determine the number of fingers to acquire, 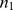, rather than evaluating all 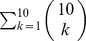 possibilities. For the general two-stage policy in Fig. 1, in stage 1 we decide on which biometrics to acquire, and after observing the acquired similarity scores, we calculate the likelihood ratio, which is the probability of observing the acquired similarity scores if the resident is genuine divided by the probability of observing the acquired similarity scores if the resident is an imposter, and decide (via two thresholds that are chosen prior to observing the acquired similarity scores) whether to accept the resident, reject the resident or continue to stage 2, where additional biometrics are acquired. After observing the similarity scores acquired in stage 2, we compute the new likelihood ratio, which is based on the cumulative biometrics acquired during both stages, and decide whether to accept or reject the resident.

The six special cases – three single-stage policies and three two-stage policies – of the general two-stage policy in Fig. 1 are described in Table 1. Because the general two-stage policy is difficult to optimize, we impose two restrictions in our three two-stage policies. First, all three two-stage policies use a different mode of biometric (i.e., fingerprints or irises) in the two different stages for each resident. The two-stage iris-finger and finger-iris policies requires every resident to provide irises and fingerprints, respectively, in the first stage and fingerprints and irises, respectively, in the second stage. The two-stage either-other policy allows either fingers or irises to be acquired in the first stage (i.e., it can vary for each resident), and the other biometric mode to be acquired in the second stage. Although the policy in Fig. 1 allows the second-stage threshold 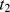 to be a function of the biometric measurements observed in the first stage, our second restriction in the three two-stage policies in Table 1 is to force the stage-two FAR to be independent of the outcome of stage one, but optimized for each resident. This restriction leads to a threshold 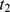 that is independent of the stage-one biometric measurements (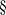
3.2 in File S1).

**Table 1:** The six classes of policies.

Policy	Additional Constraints
Single-stage finger	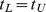 , 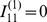
Single-stage iris	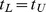 , 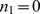
General single-stage	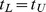
Two-stage iris-finger	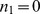 , 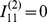
Two-stage finger-iris	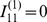 , 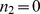
Two-stage either-other	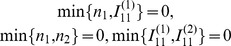

The notation used here is introduced in Fig. 1. Note that when 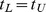, no one proceeds to the second stage.

### Delays

In addition to FRR and FAR, delays experienced by residents also play an important role in system performance. The total verification delay includes the initial pre-biometric time, where basic information such as a person’s name is collected, the image acquisition time, the operator review time, the processing time and the network delay. We perform a least squares fit of a lognormal distribution to 3 points (the probability that the total verification delay 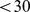 sec is 0.24, 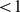 min is 0.844, and 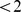 min is 0.98) for the dual-eye camera in Fig. 14 of [4], which gives an estimate of 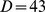 sec for the mean verification delay for both irises. Similar information for fingerprints is not reported in [5], and so we loosely estimate the difference between fingerprint delay and iris delay. The pre-biometric time is the same for fingerprints and iris and is 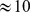 sec. The image acquisition time is 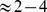 sec/finger and is 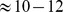 sec less than the acquisition time for irises. The operator review time (which is several seconds) and the network delay time (which is 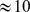 sec) are each a few seconds shorter for fingerprints than irises. While pre-biometric time is only incurred once, the network delay time is incurred twice for residents who undergo two stages of acquisition. Based on these assumptions, we use the delay times in Table 2.

**Table 2:** Delay times for both stages.

Biometrics Acquired	Delay in Stage 1 (sec)	Delay in Stage 2 (sec)
Fingers only	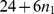	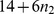
Irises only	43	33
Fingers and irises	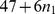	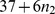

The number of fingers acquired in stage 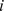 is 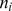 for 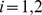.

### Optimization Problem

To optimize our proposed class of policies, we choose the parameters to minimize the FRR subject to constraints on the FAR and the average verification delay 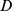, and also the additional constraints in Table 1. Mathematical derivations of the likelihood ratios, the FRR and the FAR appear in 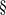
3 in File S1. For the sake of tractability, we require that each resident’s FAR be equal to the specified value. While this may be suboptimal (e.g., it may be optimal to allow a higher FAR for a person with lower similarity scores), this simplifying assumption does prevent residents from gaming the system and can be viewed as the problem of minimizing the maximum FAR over all residents.

By moving the delay constraint to the objective function via a Lagrange multiplier and solving the optimization problem for many values of the Lagrange multiplier, we can sweep out FRR vs. 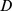 curves for a fixed FAR. We perform this procedure for FAR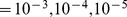 and 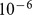. The three benchmark policies and the single-stage iris policy have fixed values of 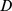 (30, 36, 43 and 43 sec, respectively because they always acquire one finger, two fingers, two irises and two irises, respectively) and are represented by points on the FRR vs. 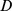 graphs. For 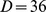 and 43 sec, we also generate FRR vs FAR graphs.

**Figure 2:**
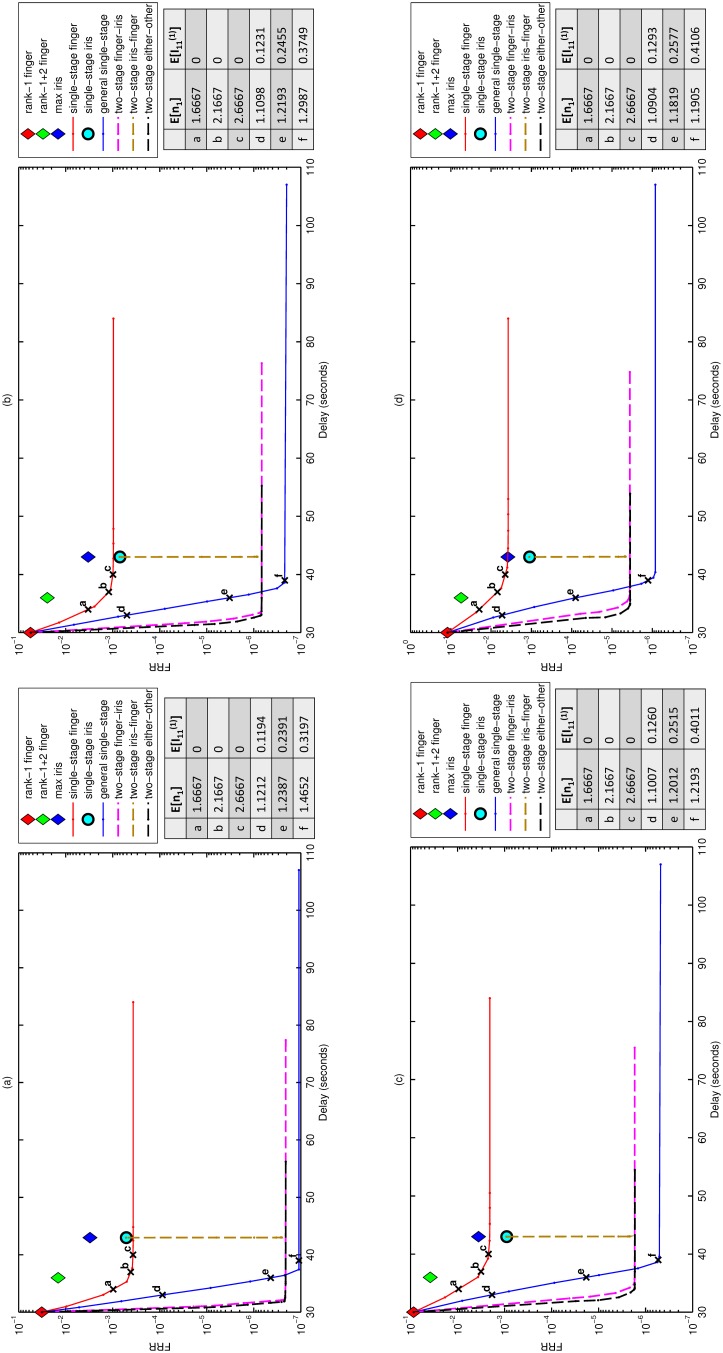
Results for the three benchmark policies and the six policies in **Table 1** in the exclusion scenario. FRR vs. verification delay tradeoff curves for FRR equals (**a**) 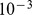, (**b**) 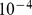, (**c**) 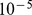 and (**d**) 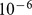. The mean number of fingers acquired per resident (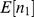) and the fraction of residents who have their irises acquired 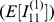 are reported for points, a,b,c,x,y,z along two of the tradeoff curves.

In our computational runs, we simulate 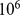 residents, each of whom are characterized by their similarity scores 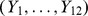 during their first verification, and then derive optimal first-stage decisions 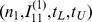 for each resident (Fig. 1). We then put each resident through the verification process 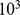 times (i.e., observing 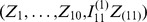 and carrying out the remainder of the process depicted in Fig. 1). With 95% confidence, the mean delays are within 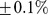, the FAR values within 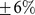, and the FRR values are within 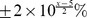 when the estimated value is 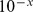; because the lowest FRR value is 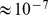, the maximum error is within 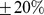, or 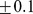 on the logarithmic scale in Fig. 2, which does not affect our qualitative insights. It took approximately one day of computing time on a quad-core 3.7 Ghz machine to generate the results for all single-stage policies in Fig. 2, while the two-stage policies took four days on a cluster of 320 cores. Therefore, using a single core, it takes 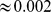 seconds to determine the optimal single-stage policy for a resident, and between 0.2–0.4 seconds to determine the optimal two-stage policy, which enables online verification.

## Results

### Parameter Estimates

The first stage of the parameter estimation procedure for fingerprints leads to reasonably accurate estimates of the rank-1 and rank-2 probabilities in both the exclusion and inclusion scenarios (Figs. 2e and 3e in File S1), with an average relative error of 19.9% over the 40 FRR probabilities in both scenarios (although it drops to 7.9% when omitting the first and tenth fingers, which have very small probabilities). In the second stage of the procedure, the lognormal imposter distribution provides an excellent fit to the known threshold-FAR pairs (Fig. 1 in File S1), predicted FRR values (for a given FAR) were nearly always within 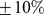 of the observed values, and the average relative error is 2.4% (Figs. 3a–c in File S1) for the inclusion scenario, whereas in the exclusion scenario five of the 21 probabilities fell outside of 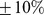 of the observed FRRs and the average relative error is 5.2% (Figs. 2a–c in File S1). In both scenarios, an out-of-sample point (a single attempt of the fusion of 2 fingers) does not predict the observed FRR to within 10% (Figs. 2d, 3d in File S1).

The fingerprint parameter values (Table 3) reveal that the finger-dependent population-wide averages 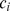 range from 0.552 (left little) to 1.313 (right index). Most of the parameter values make intuitive sense: the overall median genuine similarity score in the exclusion case is 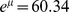, and in both scenarios the measurement error 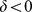, the coefficients of variation (mean divided by the standard deviation) of the interperson parameter 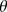 (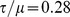 and 0.14) and the log measurement noise (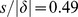 and 0.63) are modest.

**Table 3:** Parameter values for the fingerprint model.

Notation	Description	Exclusion Scenario	Inclusion Scenario
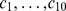	finger-dependent normalization	0.676, 0.818, 0.975, 1.179	0.552, 0.813, 1.013, 1.214
		1.193 1.282, 1.280	1.232, 1.313, 1.313
		0.998, 0.879, 0.719	1.036, 0.894, 0.620
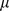	mean log genuine score	4.104	6.142
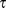	interperson standard deviation	0.579	1.700
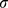	intraperson, interfinger std. dev.	1.026	0.120
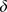	mean log measurement error	–0.796	–0.854
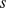	std. dev. log measurement error	0.391	0.541
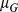	mean log imposter score	2.124	2.124
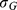	std. dev. log imposter score	0.417	0.417

The inclusion scenario incorporates the FTA rate of 0.0187.

Relative to the exclusion scenario, the inclusion scenario has slightly larger measurement errors, higher average fingerprint quality, more variable fingerprint quality across residents, and much less intraperson interfinger variability. Hence, the inclusion scenario has more residents with significantly bad quality and these residents tend to have all fingerprints of bad quality, making them difficult to correctly verify. Although the median genuine score 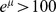 in the inclusion scenario, this is not a concern because system performance depends only on the left tail of the genuine similarity score distribution.

In the iris parameter estimation procedure, the average relative error over the five FRR probabilities is 2.7% and 1.2% in the exclusion and inclusion scenarios (Figs. 4–5 in File S1). The standard deviation of the log measurement noise is much less than the standard deviation of the log genuine scores, and the correlation between the genuine scores of the left and right iris is 0.6. As in the fingerprint case, the inclusion scenario for irises has slightly larger measurement errors, and has genuine scores with a higher mean and higher standard deviation relative to the exclusion scenario (Table 4), resulting in a fatter left tail that leads to a higher FRR.

**Table 4:** Parameter values for the iris model.

Notation	Description	Exclusion Scenario	Inclusion Scenario
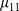	mean log genuine score	6.14	8.02
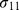	std. dev. of log genuine score	0.92	2.00
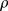	correlation of left and right genuine scores	0.6	0.6
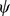	mean log measurement error	0	0
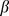	std. dev. of log measurement error	0.18	0.21
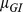	mean log imposter score	4.00	4.00
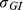	std. dev. of log imposter score	0.039	0.039

The inclusion scenario incorporates the FTA rate of 0.0033.

### Computational Results

We begin with the exclusion scenario and initially focus on the three policies that use only fingerprints. In the single-stage finger policy, the FRR, which is measured on a log scale in Fig. 2 due to the wide range of outcomes, falls by 1.5–1.7 logs when the delay is increased from 30 to 40 sec, where the reduction decreases with smaller FAR values. The FRR reduction relative to the benchmark BFD policy is 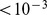 at the theoretical minimum of 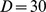 sec (where all policies are forced to use exactly one fingerprint), implying that the use of the likelihood ratio with the ranking based on 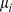 offers no significant improvement over the use of the raw similarity score with the ranking based on 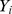. However, the single-stage finger policy achieves a 0.8–1.7 log reduction in FRR relative to the fusion policy that sums the two best fingers, where the reduction is smaller for lower FAR values. No further improvements are achieved by the single-stage finger policy by increasing the delay beyond 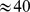 sec, which corresponds to an average of 2.7 fingers acquired per person (Fig. 2), where one finger is acquired from 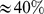 of residents, two from 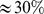, three from 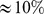, four from 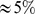, and five or more fingers from 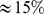 of residents.

The 0.6–0.7 log discrepancy between the two policies that use only irises is due to the fact that the benchmark policy is based on the similarity score of the maximum iris whereas the single-stage iris policy is based on the likelihood ratio of both iris scores conditioned on the iris scores during the first verification. As expected, the performance of both of these policies relative to the three fingerprint policies improves as FAR is decreased, due to the light right tail of the iris imposter distribution [9]. Indeed, the single-stage iris policy has a slightly lower FRR than the single-stage finger policy for FAR 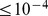, but incurs 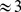 sec of additional delay.

The general single-stage policy offers 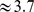 orders-of-magnitude reduction of FRR compared to the single-stage finger policy for any delay beyond 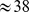 sec. At 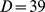 sec, this policy uses irises from 32% of residents when FAR 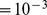, and this percentage increases to 41% when FAR drops to 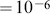. To get a sense of how our probabilistic model generates the log similarity scores during BFD and BID, 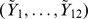, and how the individualized policy behaves, we present 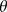 values and log similarity score vectors for 25 randomly simulated residents, along with the optimal subset of biometrics acquired under the general single-stage policy and the optimal threshold (recall that 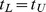) when FAR 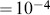 (Table 5). Of these 25 random residents, only irises are acquired from 11 residents, and only resident 6, who has lower iris scores than these eleven residents, has irises and one fingerprint (without a particularly high score) acquired. Of the remaining 13 residents, three fingerprints are acquired from two residents, two fingerprints are acquired from three residents and one fingerprint is acquired from eight residents. Some residents (e.g., residents 9 and 21) have large iris scores but do not have their irises acquired because they possess one very high fingerprint score that can be acquired more quickly. The subtlety of the optimal solution is revealed by comparing residents 4 and 14 in Table 5: resident 4 has higher iris scores and both residents have similar maximum finger scores, and yet the optimal policy acquires irises from resident 14 but not from resident 4. This is because resident 4′s second- and particularly third-best fingerprint score are higher than resident 14′s, leading to the acquisition of three fingerprints from resident 4 in lieu of irises. Finally, as expected, lower thresholds are chosen for higher BFD and BID scores of the acquired subset.

**Table 5:** Illustrative example–25 randomly simulated residents.

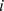	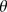	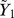	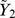	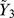	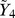	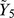	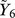	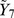	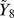	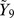	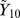	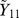	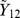	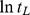
1	3.53	3.67	3.99	4.37	3.98	4.08	3.28	3.84	5.51	2.96	3.19	**5.42**	**6.25**	–42.7
2	4.21	2.28	3.89	4.07	**7.08**	4.69	5.71	4.03	4.45	1.68	3.86	6.62	6.63	−11.6
3	5.20	3.97	3.88	4.51	5.51	5.75	4.65	5.78	**6.87**	3.17	1.62	6.25	6.34	−8.7
4	3.82	3.47	1.40	2.90	**5.85**	**4.43**	**4.78**	1.29	3.00	2.40	1.07	6.55	7.45	−11.1
5	3.30	2.20	1.26	2.37	4.75	5.07	4.34	3.55	2.87	1.98	0.71	**7.55**	**7.05**	−149.3
6	3.20	2.55	1.46	2.91	4.24	0.87	2.61	**5.39**	3.86	1.11	2.53	**5.25**	**4.46**	−11.1
7	4.22	2.08	2.46	3.85	4.43	3.77	4.98	3.49	3.47	4.22	2.16	**6.02**	**5.59**	−39.5
8	3.88	1.67	3.92	4.73	1.44	4.57	3.02	4.02	1.85	4.27	2.85	**5.18**	**5.38**	−16.0
9	4.84	3.98	4.07	4.06	6.93	6.23	**7.51**	5.44	3.78	1.89	1.46	6.28	8.21	−18.0
10	3.18	3.20	2.83	3.22	4.50	2.59	3.31	1.57	2.76	3.64	2.58	**6.74**	**6.79**	−101.8
11	4.72	1.89	1.97	3.67	5.19	4.49	**5.87**	**6.49**	3.25	5.12	2.71	6.83	6.69	−22.0
12	4.67	2.73	2.99	3.80	**6.18**	**6.55**	5.44	3.22	5.62	2.41	3.26	8.14	6.45	−25.2
13	3.86	1.56	2.37	3.65	4.68	4.11	4.21	**7.13**	2.97	3.11	1.94	7.09	6.31	−12.2
14	3.49	−0.14	1.32	0.74	5.89	2.90	4.30	3.27	3.25	1.63	1.73	**6.64**	**6.09**	−73.5
15	4.14	2.31	1.63	3.31	5.64	2.91	3.43	**7.83**	1.78	3.70	1.52	7.34	6.46	−21.3
16	2.66	1.58	2.66	2.17	3.20	1.61	3.70	2.77	2.49	0.79	0.11	**6.81**	**5.93**	−75.1
17	4.56	3.29	3.60	4.69	5.48	5.10	**5.55**	**6.46**	3.74	2.53	3.20	6.92	5.96	−19.1
18	3.81	3.42	3.48	4.53	3.17	3.10	5.01	3.37	4.52	2.16	2.17	**7.11**	**6.92**	−122.6
19	4.05	2.32	0.92	3.63	3.65	3.49	**7.17**	4.34	3.96	1.13	3.92	6.77	5.85	−12.5
20	3.97	2.16	3.81	4.22	4.09	2.99	5.28	3.65	4.83	2.30	1.94	**6.78**	**6.34**	−86.5
21	4.98	3.11	5.70	4.98	5.63	7.90	**8.69**	5.04	4.10	3.29	3.67	7.99	6.03	−37.3
22	3.70	1.55	1.60	2.47	**4.69**	**5.48**	4.25	2.47	3.47	**5.43**	2.13	5.45	4.83	−13.4
23	4.16	3.08	2.01	4.67	5.20	**6.70**	5.32	5.59	4.74	2.64	3.01	7.04	6.59	−7.6
24	3.94	4.40	2.61	4.69	4.34	3.08	4.55	4.15	2.10	−0.47	0.82	**6.24**	**6.90**	−88.1
25	3.71	1.82	3.37	2.76	3.00	5.20	4.51	1.93	2.87	2.62	2.96	**6.80**	**6.14**	−80.7

For 25 randomly simulated residents indexed by 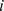, their value of 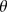, their log similarity scores during BFD and BID, 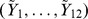, and their optimal threshold 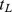 under the general single-stage policy (where 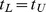) when FAR 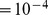. The similarity scores in boldface correspond to the optimal subset of biometrics acquired.

Turning to the three two-stage policies, the performance curve of the iris-finger policy starts at the single-stage iris policy and drops nearly vertically (Fig. 2), and achieves its improvements by using second-stage fingerprints for a very small fraction (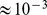) of residents with poor BID scores. However, the two-stage iris-finger policy is dominated by the general single-stage policy. In contrast, the other two two-stage policies dominate the general single-stage for small delays (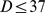 sec), but plateau at a FRR level that is higher than that of the general single-stage policy due to the restriction that the second-stage threshold is independent of the first-stage biometric observations. The more traditional FRR vs. FAR curves (Fig. 6 in File S1) reinforce some of the points above.

The results for the inclusion scenario (Figs. 7–8 in File S1) are qualitatively very similar to those in the exclusion scenario. As expected, the performance in the inclusion scenario is worse than in the exclusion scenario for all policies that use only fingers or only irises. However, for the general single-stage policy and the two-stage policies, the FRR vs. delay tradeoff curves in the inclusion scenario dominate (although just barely) the tradeoff curves in the exclusion scenario for FAR 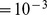. We attribute this counterintuitive result to the fact that, even though the iris and finger genuine distributions each have fatter left tails in the exclusion scenario, they also have higher means, and the general single-stage policy exploits these higher means by typically choosing to acquire either fingerprints or irises, whichever is better.

To test the accuracy of our analytical approximation, we compare the actual FARs in the simulation runs to the target FARs in the exclusion scenario (Table 1 in File S1). For policies that use only fingers, the accuracy of the FAR approximation is very high, although decreases to 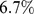 relative error when the target FAR is 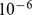. The FAR approximations are somewhat less accurate (e.g., 15% relative error for FAR 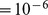) for irises, but still accurate for general single-stage policies because they primarily use fingerprints. Our analytical approximation degrades for the two-stage policies when FAR decreases to 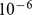 due to the difficulty in accurately estimating the denominator in equation (127) in File S1. Nonetheless, we find that when the analytical approximation errs, it overestimates the true FAR, and so is conservative with respect to satisfying the FAR constraint.

## Discussion

Our goal is to develop a fast and accurate individualized verification policy that optimizes the tradeoff between FRR, FAR and delay. A fast and accurate policy is derived by using several analytical approximations and by discovering that the fingers can be ranked according to the index 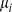, which greatly simplifies the search for a near-optimal solution. From a theoretical perspective, 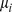 can be viewed as a more rigorous version of UIDAI’s color-coded approach to BFD, which also combines 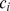 and 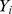 information. Substituting our parameter values into 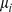, taking expectations, and scaling yields 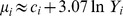 in the exclusion scenario. Because typical values are 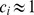 and 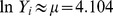, ranking by 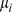 is not very different than ranking by 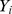; indeed, in all instances in Table 5, the largest 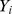 fingerprints are chosen for acquisition. More generally, the weight on 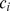 increases with the measurement error and with a resident’s image quality, and the weight on 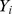 increases with the interfinger variance 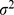.

The proposed policies perform very well. By acquiring either fingerprints or irises – but not both – from 98–99% of residents on an individualized basis, the general single-stage policy nearly achieves the ideal FRR vs. FAR tradeoff that would be obtained if all 12 biometrics were acquired from every resident, but at only a small increase in delay: compared to the minimum delay of 30 sec incurred by one finger and the maximum delay of 107 sec incurred by all 12 biometrics, the general single-stage policy achieves this performance with a delay of 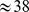 sec. This performance represents a 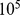-fold reduction in FRR compared to the fingerprint policies tested in [5], a 20,000-fold reduction in FRR relative to the iris policy proposed in [4] when FAR 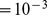 and 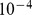, and a 5000-fold reduction in FRR compared to the iris policy proposed in [4] when FAR 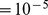 and 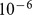. The 3.7 log FRR reduction achieved by the general single-stage policy relative to the single-stage finger policy is greater than the iris FAR of 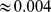 because we acquire both fingers and irises from 1–2% of residents. Among the policies tested (Table 1), the optimal policy class is nearly independent of FAR (and hence does not depend upon the level of security required), and is the two-stage finger-iris policy if the target delay 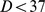 sec and the general single-stage policy if 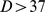 sec (Fig. 2). That is, unless there is a large marginal delay cost in the range of 30–37 sec, the optimal policy among those in Table 1 is the general single-stage policy.

The currently implemented policy (as of October 2011) is a two-stage policy that acquires everyone’s rank-1 finger in stage 1 and acquires the rank-2 finger in stage 2 if the stage-1 similarity score falls below a threshold. The FRR of this policy is at least as large as that of the benchmark policy that uses the sum of the rank-1 and rank-2 fingers, although its average delay will be smaller and will fall in the 30–36 sec range. Hence, relative to the currently implemented policy, we predict that the single-stage finger policy achieves a 0.8–1.7 log reduction in FRR and the general single-stage policy achieves an additional 3.7 log reduction.

Recall that the two-stage policies in Fig. 2 plateau at a higher FRR level than that of the general single-stage policies because we force the second-stage threshold to be independent of the first-stage biometric observations. We conjecture that the optimal general two-stage policy (i.e., the one depicted in Fig. 1 and that allows the second-stage threshold to vary with the first-stage observations) would perform nearly the same as the two-stage either-or policy for very small delays (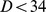 sec) because it should be optimal to use both biometric modalities in the same stage for only a small fraction of residents due to the separate setup cost (in terms of delay) each mode incurs. We also conjecture that the optimal general two-stage policy would achieve the same minimum FRR level as the general single-stage policy for large delays (e.g., 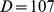 sec). Nonetheless, the general two-stage policy would incur the 10-sec network delay twice for a small fraction of residents who move on to the second stage, and consequently the general two-stage policy may not necessarily strictly dominate the general single-stage policy for all delays.

We should reiterate that our verification delay only refers to the time it takes for a resident to be verified, and does not include any queueing delays, i.e., waiting for residents in front of them in the waiting line. The queueing delays depend on a variety of factors, including the number of verification operators (more specifically, the amount of service capacity in excess of average demand), the time of day, and the statistical nature of the arrival pattern. However, for a fixed service capacity, the queueing delay is an increasing convex function of the verification time [15], and hence care should be taken in determining the mean allowable verification delay (i.e., where to reside on the FRR vs. delay curve).

### Limitations of Analysis

There are several ways to further improve performance. Our approach is essentially a minimax approach, where each resident is forced to satisfy the FAR constraint. If we enforced only an average FAR constraint over all residents, then the average FRR might be reduced by achieving very low FAR rates for residents with high-quality biometrics and allowing a higher FAR rate for residents with poor-quality biometrics; however, the average-FAR approach leads to a much more difficult mathematical problem and is more vulnerable to gaming (e.g., imposters intentionally degrading their biometric quality). Also, we have developed individualized policies based on only one set of BFD/BID measurements (i.e., the information acquired during a resident’s first verification, where considerable care is taken to obtain accurate similarity scores). Jain and Ross [16] propose individualized weights of various biometrics after gathering new data during many visits.

While a parametric approach (i.e., using a probabilistic model with specific distributional forms) is not as accurate as a nonparametric approach (e.g., constructing a simulation model based on actual 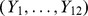 samples), a parametric approach – due to its analytical tractability – enables the development of real-time individualized verification strategies; indeed, it is not clear how one could develop a reliable (i.e., assuring that the FAR and delay constraints are satisfied and the FRR values are accurate) real-time verification strategy using a nonparametric approach.

Nonetheless, the biggest limitation of our analysis is that we estimated the model parameters in Tables 3–4 using aggregate FRR vs. FAR performance data in [4]–[5]. These performance curves cannot be uniquely inverted to derive the model parameters, and it would be more reliable to fit the distributional parameters in Tables 3–4 directly to raw distributional data from UIDAI (as noted earlier, we did not have access to such data); indeed, this would be a required next step towards the implementation of our procedure. If we had raw similarity score data, it would have been possible to have a training set to calibrate the model and a test set to compute the performance of the various policies; using only performance data, this approach was not possible here.

We suspect that our broad qualitative conclusions for the exclusion scenario for FAR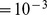 and 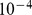 are reasonably robust because this was the FAR range for the fingerprint experiments in [5] and because huge improvements are achieved (i.e., inaccuracies due to using parametric distributions based on aggregate performance curves are likely to be much smaller than the performance gap between the benchmark policies and the proposed policies). However, the sample size of 3500 in [5] was chosen to accurately predict the FRR of the benchmark policies, not the proposed policies. Hence, although our qualitative conclusions still hold, the quantitative accuracy of the proposed policies is low because the FARs are very small (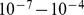) relative to the sample size in [5]. Moreover, the fingerprint portion of our model is being extrapolated to FAR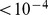 (the iris model is calibrated using FAR values as small as 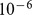), and hence the results for FAR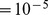 and especially FAR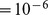 should be viewed with caution, particularly given the difficulty in reliably modeling the tails of similarity score distributions with parametric distributions [17].

On a similar note, the exclusion scenario excludes 1.87% of residents with poor fingerprint image quality (even though the fingerprint FTA rate is only 0.14%) and 0.33% of residents with iris image quality so poor that their images could not be acquired. Hence, assuming statistical independence between fingerprints and irises, less than five residents per million (i.e., 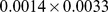) fail to generate any biometric images during acquisition. In our inclusion scenario, we assume that all residents excluded in the exclusion scenario generate fingerprint and iris similarity scores. While our inclusion scenario results for the general single-stage policy and the general two-stage policy should be viewed with skepticism (recall that under several policies, the tradeoff curves in the inclusion scenario actually dominate the tradeoff curves in the exclusion scenario for FAR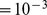), there is reason to believe that our general single-stage policy should perform very well when all residents (except the five per million who fail to acquire) are included. Even under the very conservative assumption that the 1.87% of residents who are excluded from the exclusion scenario due to poor fingerprint image quality must be verified only with irises (an analysis of the US-VISIT Program suggests that detection of poor-quality fingerprint images can be greatly improved by using 10 rather than two fingers [6]) and that the 0.33% of residents with poor iris quality must be verified only with fingerprints, a back-of-the-envelope calculation using these percentages and the FRR values in Fig. 2b suggest that for FAR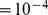, the FRR in the inclusion scenario is no larger than.
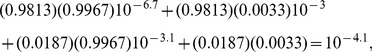
(1)


which is still 1.6 orders of magnitude lower than the FRR of the best benchmark policy in the exclusion scenario. Taken together, given the orders-of-magnitude reduction in FRR achieved by our individualized policies in our computational study, it seems safe to infer that our approach provides significant improvements, regardless of FAR value and of whether residents with poor-quality images are included or excluded.

## Supporting Information

File S1 **Supporting Material.** Explains the mathematics and the implementation of model calibration and near-optimal policies.
(PDF)
