# Supplementary material for: Analyzing Personalized Policies for Online Biometric Verification
Source: PLoS One. 2014 May 1;9(5):e94087. doi: 10.1371/journal.pone.0094087 (PMC4006790; doi:10.1371/journal.pone.0094087)
Supplement: File S1 — Supporting Material. Explains the mathematics and the implementation of model calibration and near-optimal policies. (PDF) [file pone.0094087.s001.pdf]

SUPPORTING MATERIAL

The fingerprint parameters are estimated in §1, the iris parameters are estimated in §2, and the proposed policies are derived in §3. Figs. 1-8 and Table 1 are discussed in the main text.

1 Fingerprint Parameter Estimation

The data are described in §1.1, an overview of the parameter estimation procedure is provided in §1.2, several probabilistic derivations are given in §1.3-1.6, and some computational details are described in §1.7.

1.1 The Data

Most of our data are taken from [1]. So as to have ample data to estimate the parameters of our model, we only consider the experiments in [1] that use templates (as opposed to the direct images) that are extracted from the field. We also restrict ourselves to labeled matchings, where the device provides the label (e.g., right index) of the finger, and fingerprints are only matched against those with the same label.

We use 61 probabilities that appear in Figs. 8, 10 and 11 of [1]. Fig. 8 of [1] contains the probability mass functions (PMFs) for the rank-1 finger and rank-2 finger during the best finger detection (BFD) process. That is, for the ordering convention in Fig. 8 of [1] ($i = 1, \ldots, 10$ correspond to left little, left ring, left middle, left index, left thumb, right thumb, right index, right middle, right ring, right little), we have $\hat{p}_{(1)i}$ and $\hat{p}_{(2)i}$, which is the observed probability that finger i is ranked 1 or 2, respectively.

Figs. 10-11 in [1] provide various curves – each containing seven points – depicting the false reject rate (FRR) vs. the false accept rate (FAR) during verification. We use the blue and red curves in Fig. 10 and the green curve in Fig. 11. The blue curve in Fig. 10 performs verification using the first attempt of the rank-1 finger during BFD, and we refer to the seven points on this curve as (FRR_{1k},FAR_{1k}), $k = 1, \ldots, 7$. The red curve in Fig. 10 of [1] corresponds to using up to three attempts of the rank-1 finger during BFD, and the seven points on this curve are denoted by (FRR_{1mk},FAR_{1mk}), $k = 1, \ldots, 7$. The green curve in Fig. 11 of [1] represents the fusion (i.e., sum) of the rank-1 and rank-2 fingers during BFD with up to three attempts, and these seven points are given by (FRR_{2mk},FAR_{2mk}), $k = 1, \ldots, 7$. In addition, for $l = 3, 4, 5, 6$, we know that the threshold value $\hat{t}_l = 10l$ generates the FAR value $FAR_l = 10^{-l}$ when a single finger is used.

Finally, as noted in the main text, 1.87% of residents were excluded from the verification studies due to poor image quality (pg 23-24 of [1]). Hence, we consider two scenarios. In the exclusion scenario, we assume that the 1.87% of people are omitted from the study and use the 21 FRR values directly. In the inclusion scenario, we assume that the failure-to-acquire (FTA) rate is 0.0187 and that the 21 FRR and FAR values from Figs. 10-11 in [1] are false non-match rates (FNMR) and false match rates (FMR), respectively. We then recalculate the 21 FRR and FAR values via the formulas FRR=FTA+FNMR(1-FTA) and FAR=FMR(1-FTA) (§8.3.2.2 and §8.3.3.2 of [2]).

1.2 Overview of Parameter Estimation Procedure

The experimental set-up that generates the rank-1 and rank-2 PMFs in Fig. 8 of [1] differs from the set-up used to generate the FRR vs. FAR curves in Figs. 10-11 of [1] and the FAR vs. $\hat{t}$ curve provided separately: the experimental set-up for Fig. 8 uses one very good sensor and includes the 1.87% of people that were unlikely to be verified successfully, while the latter uses the average of 14 good sensors and excludes the 1.87% of people with poor image quality. Consequently, we do not jointly estimate all of our parameter values, but rather use a two-stage approach, where we first estimate $(c_1, \ldots, c_{10})$ from the PMFs in Fig. 8 of [1] and then estimate the remaining parameter values using the FRR vs. FAR curves in Figs. 10-11 of [1] and the FAR vs. $\hat{t}$ relationship.

More specifically, let $p_{(1)i}$ and $p_{(2)i}$ for $i = 1, \ldots, 10$ be the predicted rank-1 and rank-2 probabilities corresponding to the PMFs in Fig. 8 of [1]; we derive expressions for these probabilities in terms of the model parameters in §1.3. Because the PMFs in Fig. 8 of [1] clearly provide the best data for estimating $(c_1, \ldots, c_{10})$, in the first stage we solve the least squares problem,

$$\min_{c_1,\ldots,c_{10},\mu,\tau,\sigma,s} \quad \sum_{j=1}^{2} \sum_{i=1}^{10} (p_{(j)i} - \hat{p}_{(j)i})^2 \tag{1}$$

$$\text{subject to} \quad \sum_{i=1}^{10} c_i = 10, \tag{2}$$

and retain the values of $(c_1, \ldots, c_{10})$ from the solution to (1)-(2) and discard the μ, τ, σ and s values from the solution. As we see in §1.3, the parameter δ does not feature in this optimization and the constraint (2) fixes the scaling of $(c_1, \ldots, c_{10})$.

In the second stage, we estimate the remaining parameters using the three FRR vs. FAR curves

in Figs. 10-11 of [1] and the FAR vs. $\hat{t}$ relationship. All of these FAR values were calculated using a single imposter probability distribution (using BFD fingers) that does not vary by finger i or by image quality. Let $G_1(t)$ be the cumulative distribution function (CDF) and $\bar{G}_1(t)$ be the complementary CDF of a lognormal distribution with unknown parameters (μ_G, σ_G^2). Let $G_2(t)$ and $\bar{G}_2(t)$ be the CDF and complementary CDF of the distribution that is the convolution of two lognormal (μ_G, σ_G^2) distributions. These two complementary CDFs are associated with the imposter log similarity score (i.e., of different people, one at enrollment and one at verification) for one finger and for the fusion (i.e., sum) of two fingers, respectively. Because of the difficulties in simultaneously estimating the measurement noise for genuine and imposter distributions, we do not attempt to capture the former, and hence assume that $\bar{G}_1(t)$ and $\bar{G}_2(t)$ hold even if multiple attempts are taken.

To estimate the parameters μ_G and σ_G, we use the data $(FAR_l,\ \hat{t}_l)$ for $l = 3, 4, 5, 6$. We take the FAR values as hard constraints, thereby implicitly fixing the thresholds t_l in terms of μ_G and σ_G, and minimize the sum of squared errors between the logarithm of the predicted and the observed thresholds (the presence of the ln function in (3) is explained in §1.7):

$$\min_{\mu_G, \sigma_G} \sum_{l=3}^{6} (\ln t_l - \ln \hat{t}_l)^2 \tag{3}$$

$$\text{subject to} \quad \bar{G}_1(t_l) = \text{FAR}_l \quad \text{for} \quad l = 3, 4, 5, 6. \tag{4}$$

To estimate the remaining parameters, let t_{1k} and t_{2k} for $k = 1, \ldots, 7$ be the unknown thresholds used to make accept/reject decisions, which generated the points on the FRR vs. FAR curves for the one-finger policies in Fig. 10 of [1] and the fusion policy in Fig. 11 of [1], respectively; because $\bar{G}_1(t)$ is assumed to apply in the one-finger case regardless of the number of attempts, and the FAR values provided in the blue (single-attempt rank-1 finger) and red (rank-1 finger with up to three attempts) curves in Fig 10 of [1] are the same, it follows that the thresholds are the same in the two rank-1 finger cases. Let $F_1(t)$, $F_{1m}(t)$ and $F_{2m}(t)$ be the CDFs corresponding to the similarity scores for the single-attempt rank-1 finger, the rank-1 finger with up to three attempts, and the fusion of rank-1 and rank-2 with up to three attempts, respectively, so that the predicted FRRs in these three cases are $F_1(t_{1k})$, $F_{1m}(t_{1k})$ and $F_{2m}(t_{2k})$. Expressions for these three CDFs in terms of the model parameters are derived in §1.4-1.6. We take the FAR values as hard constraints, thereby fixing the thresholds t_{1k} and t_{2k} using the estimates of μ_G and σ_G, and choose the remaining five parameters to minimize the sum of squared

relative errors (we use relative errors because the various FRR values in Figs 10-11 of [1] are of different orders of magnitude):

$$\min_{\mu,\tau,\sigma,\delta,s} \sum_{k=1}^{7} \left(\frac{F_1(t_{1k}) - \text{FRR}_{1k}}{\text{FRR}_{1k}} \right)^2 + \sum_{k=1}^{7} \left(\frac{F_{1m}(t_{1k}) - \text{FRR}_{1mk}}{\text{FRR}_{1mk}} \right)^2 + \sum_{k=1}^{7} \left(\frac{F_{2m}(t_{2k}) - \text{FRR}_{2mk}}{\text{FRR}_{2mk}} \right)^2 \tag{5}$$

$$\text{subject to} \quad \bar{G}_1(t_{1k}) = \text{FAR}_{1k} \quad \text{for} \quad k = 1, \dots, 7, \tag{6}$$

$$\bar{G}_2(t_{2k}) = \text{FAR}_{2mk} \quad \text{for} \quad k = 1, \dots, 7. \tag{7}$$

In §1.7, we discuss the details of solving (3)-(4) and (5)-(7), which include casting (3)-(4) as a simple linear regression problem, reformulating (5)-(7) into an unconstrained optimization problem by explicitly solving constraints (6)-(7) for the thresholds t_{1k} and t_{2k} using simulation, and constructing an initial solution for the optimization routine.

As explained in the main text, our model finesses some of the details of the BFD and verification processes because we do not have the raw similarity score data from [1]. More specifically, we approximate the color-coded ranking system by simply assuming that fingers are ranked solely by their Y_i values (e.g., the rank-1 finger is $\arg\max_i Y_i$). We also approximate the "up to three attempts" verification process in the derivation of $F_{1m}(t)$ and $F_{2m}(t)$ by simply assuming that three attempts are always made and the maximum score of the three attempts is used (the subscript m is mnemonic for maximum). Because fingerprints with image quality 1 or 2 should be easy to verify, this simplifying assumption should not introduce very much error.

1.3 Derivation of $p_{(1)i}$ and $p_{(2)i}$

In this section, we derive $p_{(1)i}$ and $p_{(2)j}$, which are used in equation (1). Let $\tilde{X}_i = \ln X_i \sim \mathcal{N}(c_i\theta, \sigma^2)$, where X_i is the true similarity score for finger i. Let Y_i be the observed similarity score during BFD for finger i, and let $\tilde{Y}_i = \ln Y_i$. Then $\tilde{Y}_i = \tilde{X}_i + \max_{k=1,2,3} \tilde{\epsilon}_{ik}$, where $\tilde{\epsilon}_{ik} \sim \mathcal{N}(\delta, s^2)$ are independent for $k = 1, 2, 3$.

We use Clark's method [7], which constructs an accurate approximation for the maximum of several normal random variables, to approximate $\max_{k=1,2,3} \tilde{\epsilon}_{ik}$ by a normal random variable $\tilde{\epsilon}_{mi}$ with mean

$\delta + \mu_3$ and variance σ_3^2, where

$$\mu_3 = \frac{s}{\sqrt{\pi}} \left[\Phi\left(\frac{1}{\sqrt{2\pi - 1}} \right) + \sqrt{2\pi - 1}\phi\left(\frac{1}{\sqrt{2\pi - 1}} \right) \right], \tag{8}$$

$$\sigma_3^2 = s^2 \left[1 + \frac{\sqrt{2\pi - 1}}{\pi}\phi\left(\frac{1}{\sqrt{2\pi - 1}} \right) \right] - \mu_3^2. \tag{9}$$

Thus $\tilde{Y}_i = \tilde{X}_i + \tilde{\epsilon}_{mi}$, and using the independence of $\tilde{X}_i$ and $\tilde{\epsilon}_{mi}$, we get that $\tilde{Y}_i \sim \mathcal{N}(c_i\theta + \delta + \mu_3, \alpha^2)$, where $\theta \sim \mathcal{N}(\mu, \tau^2)$ and $\alpha^2 \triangleq \sigma^2 + \sigma_3^2$. Let $\tilde{Y}_{mi} = \max_{j \neq i} \tilde{Y}_j$. Then the probability that finger i is chosen as the best finger equals $P(\tilde{Y}_i > \tilde{Y}_{mi})$. There are different ways to derive this quantity, and we use an approach that is amenable to numerical integration:

$$\begin{aligned}
p_{(1)i} &= P(\tilde{Y}_i > \tilde{Y}_{mi}), \\
&= E\left[P(\tilde{Y}_i > \tilde{Y}_{mi} | \theta, \tilde{Y}_i) \right], \\
&= E\left[\prod_{j \neq i}^{10} P(\tilde{Y}_i > \tilde{Y}_j | \theta, \tilde{Y}_i) \right], \\
&= \int_\theta \int_{\tilde{Y}_i} \left(\prod_{j \neq i}^{10} \Phi\left(\frac{\tilde{Y}_i - (c_j\theta + \delta + \mu_3)}{\alpha} \right) \right) \frac{e^{-\frac{(\tilde{Y}_i - (c_i\theta + \delta + \mu_3))^2}{2\alpha^2}}}{\sqrt{2\pi}\alpha} \frac{e^{-\frac{(\theta - \mu)^2}{2\tau^2}}}{\sqrt{2\pi}\tau} \, d\tilde{Y}_i \, d\theta, \qquad (10) \\
&= \int_{\hat{\theta}} \int_{y_i} h_1(\hat{\theta}, y_i) \frac{e^{-\hat{\theta}^2} e^{-y_i^2}}{\pi} \, dy_i \, d\hat{\theta}, \qquad (11)
\end{aligned}$$

where

$$h_1(\hat{\theta}, y_i) \triangleq \prod_{j \neq i}^{10} \Phi\left(\sqrt{2}y_i + (c_i - c_j)\frac{\mu + \sqrt{2}\tau\hat{\theta}}{\alpha} \right). \tag{12}$$

The change of variable in (11) converts the integral in (10) into a form where we can apply Gauss-Hermite quadrature, which is well-suited for functions that require integration of the normal density ([4], pg 129). Gauss-Hermite quadrature approximates $\int_{-\infty}^{\infty} f(v)e^{-v^2} dv$ by $\sum_{i=1}^{n} w_i f(v_i)$, where N is the number of sample points for the approximation, and the points v_i and the associated weights w_i are fixed once n is chosen. This procedure yields

$$p_{(1)i} \triangleq \frac{1}{\pi} \sum_{k=1}^{N} \sum_{l=1}^{N} h_1(v_k, v_l) w_k w_l. \tag{13}$$

We perform similar calculations to derive $p_{(2)i}$. If we define $\tilde{Y}_{mij} = \max_{k \neq i,j} \tilde{Y}_k$, then

$$
\begin{aligned}
p_{(2)i} &= \sum_{j \neq i}^{10} P(\tilde{Y}_{mij} < \tilde{Y}_i < \tilde{Y}_j), \\
&= \sum_{j \neq i}^{10} E[P(\tilde{Y}_{mij} < \tilde{Y}_i < \tilde{Y}_j | \theta, \tilde{Y}_i)], \\
&= \sum_{j \neq i}^{10} E[P(\tilde{Y}_{mij} < \tilde{Y}_i | \theta, \tilde{Y}_i)\ P(\tilde{Y}_i < \tilde{Y}_j | \theta, \tilde{Y}_i)], \\
&= \sum_{j \neq i}^{10} E\left[\left(\prod_{k \neq i,j}^{10} P(\tilde{Y}_k < \tilde{Y}_i | \theta, \tilde{Y}_i)\right)\ P(\tilde{Y}_i < \tilde{Y}_j | \theta, \tilde{Y}_i)\right], \\
&= \sum_{j \neq i}^{10} E\left[\left(\prod_{k \neq i}^{10} P(\tilde{Y}_k < \tilde{Y}_i | \theta, \tilde{Y}_i)\right)\ \frac{P(\tilde{Y}_i < \tilde{Y}_j | \theta, \tilde{Y}_i)}{P(\tilde{Y}_j < \tilde{Y}_i | \theta, \tilde{Y}_i)}\right], \\
&= \sum_{j \neq i}^{10} E\left[\left(\prod_{k \neq i}^{10} P(\tilde{Y}_k < \tilde{Y}_i | \theta, \tilde{Y}_i)\right)\ \left(\frac{1}{P(\tilde{Y}_j < \tilde{Y}_i | \theta, \tilde{Y}_i)} - 1\right)\right], \\
&= E\left[\sum_{j \neq i}^{10}\left(\prod_{k \neq i}^{10} P(\tilde{Y}_k < \tilde{Y}_i | \theta, \tilde{Y}_i)\right)\ \left(\frac{1}{P(\tilde{Y}_j < \tilde{Y}_i | \theta, \tilde{Y}_i)} - 1\right)\right], \\
&= E\left[\left(\prod_{k \neq i}^{10} P(\tilde{Y}_k < \tilde{Y}_i | \theta, \tilde{Y}_i)\right)\ \sum_{j \neq i}^{10}\left(\frac{1}{P(\tilde{Y}_j < \tilde{Y}_i | \theta, \tilde{Y}_i)} - 1\right)\right], \\
&= E\left[\left(\prod_{j \neq i}^{10} P(\tilde{Y}_j < \tilde{Y}_i | \theta, \tilde{Y}_i)\right)\ \left(\sum_{j \neq i}^{10}\frac{1}{P(\tilde{Y}_j < \tilde{Y}_i | \theta, \tilde{Y}_i)} - (m-1)\right)\right], \\
&= E\left[\left(\prod_{j \neq i}^{10} \Phi\left(\frac{\tilde{Y}_i - (c_j\theta + \mu_3 + \delta)}{\alpha}\right)\right)\ \left(\sum_{j \neq i}^{10}\frac{1}{\Phi\left(\frac{\tilde{Y}_i - (c_j\theta + \mu_3 + \delta)}{\alpha}\right)} - (m-1)\right)\right], \\
&= \int_\theta \int_{\tilde{Y}_i} \left(\prod_{j \neq i}^{10} \Phi\left(\frac{\tilde{Y}_i - (c_j\theta + \mu_3 + \delta)}{\alpha}\right)\right)\ \left(\sum_{j \neq i}^{10}\frac{1}{\Phi\left(\frac{\tilde{Y}_i - (c_j\theta + \mu_3 + \delta)}{\alpha}\right)} - (m-1)\right) \\
&\qquad \cdot \frac{e^{-\frac{(\tilde{Y}_i - (c_i\theta + \mu_3 + \delta))^2}{2\alpha^2}}}{\sqrt{2\pi}\alpha}\ \frac{e^{-\frac{(\theta-\mu)^2}{2\tau^2}}}{\sqrt{2\pi}\tau}\ d\tilde{Y}_i\ d\theta. \qquad (14)
\end{aligned}
$$

Approximating the double integral in (14) with Gauss-Hermite quadrature with N sample points v_i and associated weights w_i, we get

$$p_{(2)i} \triangleq \frac{1}{\pi} \sum_{k=1}^{N} \sum_{l=1}^{N} h_2(v_k, v_l) w_k w_l, \qquad (15)$$

where

$$h_2(\hat{\theta}, y_i) \triangleq h_1(\hat{\theta}, y_i) \left[-(m-1) + \sum_{j \neq i}^{10} \frac{1}{\Phi\left(\sqrt{2}y_i + (c_i - c_j)\frac{\mu + \sqrt{2}\tau\hat{\theta}}{\alpha}\right)} \right]. \tag{16}$$

Note from (12) and (16) that neither $h_1(\hat{\theta}, y_i)$ nor $h_2(\hat{\theta}, y_i)$ depend on δ, and hence $p_{1(i)}$ and $p_{2(i)}$ do not depend on δ for i= $1, \ldots, 10$. Further, we can always scale the parameters $(c_1, \ldots, c_{10}), \sigma$ and s to obtain scaled values of $(\tilde{Y}_1, \ldots, \tilde{Y}_{10})$, which nevertheless preserve the probabilities $p_{1(i)}$ and $p_{2(i)}$ for i= $1, \ldots, 10$. This justifies constraint (2), which fixes the scaling.

1.4 Derivation of $F_1(t_{1k})$

As explained in §1.3, $\tilde{X}_i$ is the true log genuine similarity score for finger i and $\tilde{Y}_i = \tilde{X}_i + \tilde{\epsilon}_{mi}$ is the log genuine similarity score observed during the BFD process, so that $\tilde{Y}_i = \ln Y_i \sim \mathcal{N}(c_i\theta + \delta + \mu_3, \sigma^2 + \sigma_3^2)$. Let the subscripts (i) be defined by $Y_{(1)} \geq \cdots \geq Y_{(10)}$, regardless of which random variables the subscripts appear on. Let $\tilde{Z}_i = \tilde{X}_i + \tilde{\delta}_i$ be the log genuine similarity score during verification, where $\tilde{\delta}_i \sim \mathcal{N}(\delta, s^2)$ and is independent of $\tilde{\epsilon}_{mi}$. It follows that $\tilde{Z}_i = \ln Z_i \sim \mathcal{N}(c_i\theta + \delta, \sigma^2 + s^2)$. Recalling that $F_1(t)$ is the CDF of the genuine similarity score in the single-attempt rank-1 finger scenario, we have that the FRR for this case is

$$F_1(t_{1k}) = P(\tilde{Z}_{(1)} \leq \ln t_{1k}). \tag{17}$$

Our goal in this section is to derive the CDF of $\tilde{Z}_{(1)}$.

Recalling that $\tilde{Y}_{mi} = \max_{j \neq i} \tilde{Y}_j$, we have that

$$P(\tilde{Z}_{(1)} < t) = \sum_{i=1}^{10} P(\tilde{Z}_i < t, \tilde{Y}_i > \tilde{Y}_{mi}). \tag{18}$$

We use the tower property in conjunction with conditioning on variables that – to ease the computational complexity – make the events within the probability conditionally independent. In particular, we condition on θ and $\tilde{Y}_i$ because

$$\begin{aligned} P(\tilde{Z}_i < t, \tilde{Y}_i > \tilde{Y}_{mi}) =& E\left[P(\tilde{Z}_i < t, \tilde{Y}_i > \tilde{Y}_{mi} | \theta, \tilde{Y}_i)\right], \\ =& E\left[P(\tilde{Z}_i < t | \theta, \tilde{Y}_i) P(\tilde{Y}_i > \tilde{Y}_{mi} | \theta, \tilde{Y}_i)\right], \\ =& E\left[P(\tilde{X}_i + \tilde{\delta}_i < t | \theta, \tilde{Y}_i) P(\tilde{Y}_i > \tilde{Y}_{mi} | \theta, \tilde{Y}_i)\right]. \end{aligned} \tag{19}$$

To evaluate $P(\tilde{X}_i + \tilde{\delta}_i < t|\theta, \tilde{Y}_i)$ in (19), we apply Prop. 3.13 on page 116 of [5], which gives the distribution of one normal random vector conditioned on another (possibly correlated) random vector. This result implies that given $(\theta, \tilde{Y}_i)$, $\tilde{X}_i \sim \mathcal{N}(\mu_{i|\tilde{Y}_i}, \hat{\sigma}^2)$, where

$$\mu_{i|\tilde{Y}_i} = \frac{c_i\theta/\sigma^2 + (\tilde{Y}_i - \delta - \mu_3)/\sigma_3^2}{1/\sigma^2 + 1/\sigma_3^2}, \tag{20}$$

$$\hat{\sigma}^2 = \frac{1}{1/\sigma^2 + 1/\sigma_3^2}. \tag{21}$$

It follows that

$$\begin{aligned}
P(\tilde{Z}_{(1)} < t) &= \sum_{i=1}^{10} E\left[P(\tilde{X}_i + \tilde{\delta}_i < t|\theta, \tilde{Y}_i) P(\tilde{Y}_i > \tilde{Y}_{mi}|\theta, \tilde{Y}_i)\right] \quad \text{by} \ \ (18) - (19), \\
&= \sum_{i=1}^{10} E\left[\Phi\left(\frac{t - \mu_{i|\tilde{Y}_i} - \delta}{\sqrt{\hat{\sigma}^2 + s^2}}\right) \prod_{j\neq i}^{10} P(\tilde{Y}_i > \tilde{Y}_j|\theta, \tilde{Y}_i)\right] \quad \text{by} \ \ (20) - (21), \\
&= \sum_{i=1}^{10} E\left[\Phi\left(\frac{t - \mu_{i|\tilde{Y}_i} - \delta}{\sqrt{\hat{\sigma}^2 + s^2}}\right) \prod_{j\neq i}^{10} \Phi\left(\frac{\tilde{Y}_i - (c_j\theta + \mu_3 + \delta)}{\alpha}\right)\right]. \qquad (22)
\end{aligned}$$

Finally, we employ a change of measure in conjunction with numerical integration to reduce the number of $\Phi(\cdot)$ evaluations needed at each approximation point in the double integral version of (22). Given θ, the random variables $(\tilde{Y}_1, \ldots, \tilde{Y}_{10})$ inside the expectation in (22) are normally distributed with the same variance. We use Girsanov's theorem ([6], §3.5) to change the means of $(\tilde{Y}_1, \ldots, \tilde{Y}_{10})$ so that given θ, they all have the same distribution, although this will require a compensating multiplicative term in the expectation.

Recalling that $\tilde{Y}_i \sim \mathcal{N}(c_i\theta + \delta + \mu_3, \alpha^2)$, we define $\tilde{Y} \sim \mathcal{N}(\bar{c}\theta + \delta + \mu_3, \alpha^2)$ for a constant $\bar{c}$ that may be chosen arbitrarily, and let

$$a_{iY} \triangleq \exp\left\{-\frac{(c_i - \bar{c})\theta\left[(c_i - \bar{c})\theta - 2\left(\tilde{Y} - (\bar{c}\theta + \delta + \mu_3)\right)\right]}{2\alpha^2}\right\}. \tag{23}$$

Then, given θ, Girsanov's theorem implies that for any function $h(\cdot)$,

$$E[h(\tilde{Y}_i)] = E[h(\tilde{Y})a_{iY}] \quad \text{for} \quad i = 1, \ldots, 10. \tag{24}$$

Applying (24) to (22) yields

$$\begin{aligned}
P(\tilde{Z}_{(1)} < t) &= \sum_{i=1}^{10} E\left[\Phi\left(\frac{t-\mu_{i|\tilde{Y}_i}-\delta}{\sqrt{\hat{\sigma}^2+s^2}}\right)\prod_{j\neq i}^{10}\Phi\left(\frac{\tilde{Y}_i-(c_j\theta+\mu_3+\delta)}{\alpha}\right)\right], \\
&= \sum_{i=1}^{10} E\left[a_{iY}\Phi\left(\frac{t-\mu_{i|\tilde{Y}}-\delta}{\sqrt{\hat{\sigma}^2+s^2}}\right)\prod_{j\neq i}^{10}\Phi\left(\frac{\tilde{Y}-(c_j\theta+\mu_3+\delta)}{\alpha}\right)\right] \quad \text{by} \quad (24), \\
&= \sum_{i=1}^{10} E\left[a_{iY}\Phi\left(\frac{t-\mu_{i|\tilde{Y}}-\delta}{\sqrt{\hat{\sigma}^2+s^2}}\right)\frac{\prod_{j=1}^{10}\Phi\left(\frac{\tilde{Y}-(c_j\theta+\mu_3+\delta)}{\alpha}\right)}{\Phi\left(\frac{\tilde{Y}-(c_i\theta+\mu_3+\delta)}{\alpha}\right)}\right], \\
&= E\left\{\left[\prod_{j=1}^{10}\Phi\left(\frac{\tilde{Y}-(c_j\theta+\mu_3+\delta)}{\alpha}\right)\right]\sum_{i=1}^{10}\frac{a_{iY}\Phi\left(\frac{t-\mu_{i|\tilde{Y}}-\delta}{\sqrt{\hat{\sigma}^2+s^2}}\right)}{\Phi\left(\frac{\tilde{Y}-(c_i\theta+\mu_3+\delta)}{\alpha}\right)}\right\}. \qquad (25)
\end{aligned}$$

1.5 Derivation of $F_{1m}(t_{1k})$

We now consider the maximum of three verification attempts with the best finger. Let $\tilde{Z}_{ij} = \tilde{X}_i + \tilde{\delta}_{ij}$, where $\tilde{\delta}_{ij} \sim \mathcal{N}(\delta, s^2)$ are independent for $j = 1, 2, 3$, and define the best observed log measurement as $\tilde{Z}_{mi} = \tilde{X}_i + \max_{1\leq j\leq 3}\tilde{\delta}_{ij}$. Repeating the steps in §1.3, we use Clark's method [7] to approximate $\max_{1\leq j\leq 3}\tilde{\delta}_{ij}$ by a normal random variable $\tilde{\delta}_{mi}$ with mean $\delta+\mu_3$ and variance σ_3^2 given in (8)-(9). Thus, we have $\tilde{Z}_{mi} = \tilde{X}_i + \tilde{\delta}_{mi}$ where $\tilde{X}_i$ and $\tilde{\delta}_{mi}$ are independent. The FRR in this case is

$$F_{1m}(t_{1k}) = P(\tilde{Z}_{m(1)} \leq \ln t_{1k}), \qquad (26)$$

and our goal is to derive the CDF of $\tilde{Z}_{m(1)}$.

Noting that $P(\tilde{Z}_{m(1)} < t) = P(\tilde{X}_{(1)} + \tilde{\delta}_{m(1)} < t) = P(\tilde{X}_{(1)} + \tilde{\delta}_{m(1)} - \mu_3 < t - \mu_3)$ and comparing this expression to $P(\tilde{Z}_{(1)} < t) = P(\tilde{X}_{(1)} + \tilde{\delta}_{(1)} < t)$ from §1.4, we infer that equation (25) can be generalized to three attempts by substituting $t-\mu_3$ for t and σ_3^2 for s^2, which yields

$$P(\tilde{Z}_{m(1)} < t) = E\left\{\left[\prod_{j=1}^{10}\Phi\left(\frac{\tilde{Y}-(c_j\theta+\mu_3+\delta)}{\alpha}\right)\right]\sum_{i=1}^{10}\frac{a_{iY}\Phi\left(\frac{t-\mu_3-\mu_{i|\tilde{Y}}-\delta}{\sqrt{\hat{\sigma}^2+\sigma_3^2}}\right)}{\Phi\left(\frac{\tilde{Y}-(c_i\theta+\mu_3+\delta)}{\alpha}\right)}\right\}. \qquad (27)$$

1.6 Derivation of $F_{2m}(t_{2k})$

This section considers the most complicated case: the fusion (i.e., sum) of the best of the three attempts for the best two fingers. The FRR in this case is

$$F_{2m}(t_{2k}) = P(e^{\tilde{Z}_{m(1)}} + e^{\tilde{Z}_{(2)m}} \leq \ln t_{2k}), \tag{28}$$

Recall that $\tilde{Y}_{mij} = \max_{k\neq i,j} \tilde{Y}_k$, which is the maximum of eight random variables. Our goal is to derive the CDF for $e^{\tilde{Z}_{m(1)}} + e^{\tilde{Z}_{m(2)}}$, which can be expressed as

$$P(e^{\tilde{Z}_{m(1)}} + e^{\tilde{Z}_{m(2)}} < t) = \sum_{\substack{i,j \\ i\neq j}}^{10} P(e^{\tilde{Z}_{mi}} + e^{\tilde{Z}_{mj}} < t, \tilde{Y}_i > \tilde{Y}_j > \tilde{Y}_{mij}). \tag{29}$$

We begin by repeating the steps in §1.4: condition on certain quantities and use the tower property, use Prop. 3.13 in [5] to calculate the conditional expectations, and use Girsanov's theorem to reduce the computational complexity. Although there are many possible combinations of variables to condition on, we find it computationally convenient to condition on $(\theta, \tilde{Y}_j, \tilde{Z}_{mi})$, which yields

$$\begin{aligned}
&P(e^{\tilde{Z}_{mi}} + e^{\tilde{Z}_{mj}} < t, \tilde{Y}_i > \tilde{Y}_j > \tilde{Y}_{mij}) \\
&= E\left[P(e^{\tilde{Z}_{mi}} + e^{\tilde{Z}_{mj}} < t, \tilde{Y}_i > \tilde{Y}_j > \tilde{Y}_{mij}|\theta, \tilde{Y}_j, \tilde{Z}_{mi})\right], \\
&= E\left[P(e^{\tilde{Z}_{mi}} + e^{\tilde{Z}_{mj}} < t|\theta, \tilde{Y}_j, \tilde{Z}_{mi})P(\tilde{Y}_i > \tilde{Y}_j|\theta, \tilde{Y}_j, \tilde{Z}_{mi})P(\tilde{Y}_j > \tilde{Y}_{mij}|\theta, \tilde{Y}_j)\right], \\
&= E\left[P(e^{\tilde{X}_j+\tilde{\delta}_{mj}} < t - e^{\tilde{Z}_{mi}}|\theta, \tilde{Y}_j, \tilde{Z}_{mi})P(\tilde{X}_i + \tilde{\epsilon}_{mi} > \tilde{Y}_j|\theta, \tilde{Y}_j, \tilde{Z}_{mi})P(\tilde{Y}_j > \tilde{Y}_{mij}|\theta, \tilde{Y}_j)\right], \\
&= E\left[P(\tilde{X}_j + \tilde{\delta}_{mj} < \ln(t - e^{\tilde{Z}_{mi}})^+|\theta, \tilde{Y}_j, \tilde{Z}_{mi})P(\tilde{X}_i + \tilde{\epsilon}_{mi} > \tilde{Y}_j|\theta, \tilde{Y}_j, \tilde{Z}_{mi})P(\tilde{Y}_j > \tilde{Y}_{mij}|\theta, \tilde{Y}_j)\right],
\end{aligned} \tag{30}$$

where $x^+ = \max\{x, 0\}$.

As in §1.5, we approximate $\tilde{\delta}_{mj}$ in (30) by a normal random variable with mean $\delta + \mu_3$ and variance σ_3^2 given in (8)-(9). Thus, given θ, $\tilde{Z}_{mj}$ is approximately distributed as $\mathcal{N}(c_j\theta + \delta + \mu_3, \sigma^2 + \sigma_3^2)$, where we use the independence of $\tilde{X}_j$ and $\tilde{\delta}_{mj}$ in calculating the variance. In (30), we are interested in calculating the conditional distribution of $\tilde{X}_i$ given $\tilde{Z}_{mi}$ and θ. Noting that $\tilde{Z}_{mi} = \tilde{X}_i + \tilde{\delta}_{mi}$, and using Prop. 3.13 in [5], we find that

$$\tilde{X}_{i|\tilde{Z}_{mi}} \sim \mathcal{N}(\mu_{i|\tilde{Z}_{mi}}, \hat{\sigma}^2), \tag{31}$$

where $\hat{\sigma}^2$ is given in (21) and

$$\mu_{i|\tilde{Z}_{mi}} = \frac{c_i\theta/\sigma^2 + (\tilde{Z}_{mi} - \delta - \mu_3)/\sigma_3^2}{1/\sigma^2 + 1/\sigma_3^2}. \tag{32}$$

Referring again to (30), we already know $\tilde{X}_{j|\tilde{Y}_j} \sim \mathcal{N}(\mu_{j|\tilde{Y}_j}, \hat{\sigma}^2)$ from Section §1.4. Based on this fact and (29)-(32), we calculate the first two probabilities inside the expectation in (30) to get

$$\begin{aligned}
&P(e^{\tilde{Z}_{m(1)}} + e^{\tilde{Z}_{m(2)}} < t) \\
=&\sum_{\substack{i,j\\ i\neq j}}^{10} E\left[\Phi\left(\frac{\ln(t - e^{\tilde{Z}_{mi}})^+ - \mu_{j|\tilde{Y}_j} - \delta - \mu_3}{\sqrt{\hat{\sigma}^2 + \sigma_3^2}}\right)\bar{\Phi}\left(\frac{\tilde{Y}_j - \mu_{i|\tilde{Z}_{mi}} - \delta - \mu_3}{\sqrt{\hat{\sigma}^2 + \sigma_3^2}}\right) P(\tilde{Y}_j > \tilde{Y}_{mij}|\theta, \tilde{Y}_j)\right], \\
=&\sum_{\substack{i,j\\ i\neq j}}^{10} E\left[\Phi\left(\frac{\ln(t - e^{\tilde{Z}_{mi}})^+ - \mu_{j|\tilde{Y}_j} - \delta - \mu_3}{\sqrt{\hat{\sigma}^2 + \sigma_3^2}}\right)\bar{\Phi}\left(\frac{\tilde{Y}_j - \mu_{i|\tilde{Z}_{mi}} - \delta - \mu_3}{\sqrt{\hat{\sigma}^2 + \sigma_3^2}}\right) \prod_{n\neq i,j}^{10} P(\tilde{Y}_j > \tilde{Y}_n|\theta, \tilde{Y}_j)\right], \\
=&\sum_{\substack{i,j\\ i\neq j}}^{10} E\left[\Phi\left(\frac{\ln(t - e^{\tilde{Z}_{mi}})^+ - \mu_{j|\tilde{Y}_j} - \delta - \mu_3}{\sqrt{\hat{\sigma}^2 + \sigma_3^2}}\right)\bar{\Phi}\left(\frac{\tilde{Y}_j - \mu_{i|\tilde{Z}_{mi}} - \delta - \mu_3}{\sqrt{\hat{\sigma}^2 + \sigma_3^2}}\right) \prod_{n\neq i,j}^{10} \Phi\left(\frac{\tilde{Y}_j - (c_n\theta + \delta + \mu_3)}{\alpha}\right)\right].
\end{aligned} \tag{33}$$

Turning to the change of measure, we take advantage of the fact that, given θ, $\tilde{Y}_j$ and $\tilde{Z}_{mi}$ are independent for $i \neq j$. Given θ, we change the measure of $\tilde{Y}_j, \tilde{Z}_{mi}$ in (33) to $\tilde{Y} \sim \mathcal{N}(\bar{c}\theta+\delta+\mu_3, \alpha^2), \tilde{Z}_m \sim \mathcal{N}(\bar{c}\theta + \mu_3, \alpha^2)$, and compensate by multiplying by the corresponding terms

$$a_{jY} \triangleq \exp\left\{-\frac{(c_j - \bar{c})\theta\left[(c_j - \bar{c})\theta - 2\left(\tilde{Y} - (\bar{c}\theta + \delta + \mu_3)\right)\right]}{2\alpha^2}\right\}, \tag{34}$$

$$a_{iZ_m} \triangleq \exp\left\{-\frac{(c_i - \bar{c})\theta\left[(c_i - \bar{c})\theta - 2\left(\tilde{Z}_m - (\bar{c}\theta + \delta + \mu_3)\right)\right]}{2\alpha^2}\right\}, \tag{35}$$

to get

$$
\begin{aligned}
&P(e^{\tilde{Z}_{m(1)}} + e^{\tilde{Z}_{m(2)}} < t) \\
=&\sum_{\substack{i,j\\ i\neq j}}^{10} E\left[\Phi\left(\frac{\ln(t-e^{\tilde{Z}_{mi}})^+ - \mu_{j|\tilde{Y}_j} - \delta - \mu_3}{\sqrt{\hat{\sigma}^2+\sigma_3^2}}\right)\bar{\Phi}\left(\frac{\tilde{Y}_j - \mu_{i|\tilde{Z}_{mi}} - \delta - \mu_3}{\sqrt{\hat{\sigma}^2+\sigma_3^2}}\right)\prod_{n\neq i,j}^{10}\Phi\left(\frac{\tilde{Y}_j - (c_n\theta+\delta+\mu_3)}{\alpha}\right)\right] \\
=&\sum_{\substack{i,j\\ i\neq j}}^{10} E\left[\Phi\left(\frac{\ln(t-e^{\tilde{Z}_{m}})^+ - \mu_{j|\tilde{Y}} - \delta - \mu_3}{\sqrt{\hat{\sigma}^2+\sigma_3^2}}\right)\bar{\Phi}\left(\frac{\tilde{Y} - \mu_{i|\tilde{Z}_{m}} - \delta - \mu_3}{\sqrt{\hat{\sigma}^2+\sigma_3^2}}\right)\right. \\
&\qquad\qquad\left.\cdot\prod_{n\neq i,j}^{10}\Phi\left(\frac{\tilde{Y} - (c_n\theta+\delta+\mu_3)}{\alpha}\right)a_{iZ_m}a_{jY}\right], \\
=&E\left\{\left[\prod_{n=1}^{10}\Phi\left(\frac{\tilde{Y}-(c_n\theta+\delta+\mu_3)}{\alpha}\right)\right]\sum_{\substack{i,j\\ i\neq j}}^{10}\frac{\Phi\left(\frac{\ln(t-e^{\tilde{Z}_m})^+ - \mu_{j|\tilde{Y}} - \delta-\mu_3}{\sqrt{\hat{\sigma}^2+\sigma_3^2}}\right)\bar{\Phi}\left(\frac{\tilde{Y}-\mu_{i|\tilde{Z}_m}-\delta-\mu_3}{\sqrt{\hat{\sigma}^2+\sigma_3^2}}\right)a_{iZ_m}a_{jY}}{\Phi\left(\frac{\tilde{Y}-(c_i\theta+\delta+\mu_3)}{\alpha}\right)\Phi\left(\frac{\tilde{Y}-(c_j\theta+\delta+\mu_3)}{\alpha}\right)}\right\}.
\end{aligned}
\tag{36}
$$

We note that this change of measure is more efficient than applying Clark's approximation to the last probability in (33) and then invoking a change of measure.

Because the expression in (36) is difficult to compute, we perform one final step, which uses a monomial-based integrating scheme called cubature [3] combined with the Gauss-Laguerre scheme [4]. Suppose we need to calculate for any function $f(\cdot)$ on $\mathbb{R}^3$,

$$
E\left[f(\theta,\tilde{Y},\tilde{Z}_m)\right] = \int_{-\infty}^{\infty}\int_{-\infty}^{\infty}\int_{-\infty}^{\infty} f(\theta,y,z)\frac{e^{-(\theta-\mu)^2/2\tau^2}}{\sqrt{2\pi}\tau}\frac{e^{-(y-\bar{c}\theta-\delta-\mu_3)^2/2\alpha^2}}{\sqrt{2\pi}\alpha}\frac{e^{-(z-(\bar{c}\theta+\delta+\mu_3))^2/2\alpha^2}}{\sqrt{2\pi}\alpha}\,d\theta\,dy\,dz. \tag{37}
$$

Consider the substitution

$$
\hat{\theta} = \frac{\theta-\mu}{\sqrt{2}\tau} \Rightarrow d\hat{\theta} = \frac{d\theta}{\sqrt{2}\tau}, \qquad \theta = \mu + \sqrt{2}\tau\hat{\theta}, \tag{38}
$$

$$
\hat{y} = \frac{y-(\bar{c}\theta+\delta+\mu_3)}{\sqrt{2}\alpha} \Rightarrow d\hat{y} = \frac{dy}{\sqrt{2}\alpha}, \qquad y = \bar{c}\theta+\delta+\mu_3+\sqrt{2}\alpha\hat{y}, \tag{39}
$$

$$
\hat{z} = \frac{z-(\bar{c}\theta+\delta+\mu_3)}{\sqrt{2}\alpha} \Rightarrow d\hat{z} = \frac{dz}{\sqrt{2}\alpha}, \qquad z = \bar{c}\theta+\delta+\mu_3+\sqrt{2}\alpha\hat{z}. \tag{40}
$$

In shorthand, we denote the relations on the right side of (38)-(40) by $(\theta,y,z) = H_1(\hat{\theta},\hat{y},\hat{z})$. Making this

substitution into (37) yields

$$E\left[f(\theta,\tilde{Y},\tilde{Z}_m)\right] = \int_{-\infty}^{\infty}\int_{-\infty}^{\infty}\int_{-\infty}^{\infty} f(H_1(\hat{\theta},\hat{y},\hat{z}))\frac{e^{-(\hat{\theta}^2+\hat{y}^2+\hat{z}^2)}}{\pi^{3/2}}\,d\hat{\theta}\;d\hat{y}\;d\hat{z}. \tag{41}$$

We now convert to spherical coordinates as follows: let $(\hat{\theta},\hat{y},\hat{z}) = H_2(r,\mathbf{u}) \triangleq (ru_1, ru_2, ru_3)$, and let U_3 represent the surface of a unit sphere in three dimensions with $dA(\mathbf{u})$ representing its infinitesimal area element. Substituting into (41), we get

$$\begin{aligned} E\left[f(\theta,\tilde{Y},\tilde{Z}_m)\right] &= \int_{r=0}^{\infty}\int_{U_3} f(H_1(H_2(r,\mathbf{u})))\frac{e^{-r^2}}{\pi^{3/2}}r^2\;dA(\mathbf{u})\,dr, \\ &= \int_{r=0}^{\infty}\frac{e^{-r^2}}{\pi^{3/2}}r^2\underbrace{\int_{U_3} f(H_1(H_2(r,\mathbf{u})))\;dA(\mathbf{u})}_{S(r)}\;dr, \qquad (42) \\ &= \int_{r=0}^{\infty}\frac{e^{-r^2}}{\pi^{3/2}}r^2 S(r)\,dr. \qquad (43) \end{aligned}$$

Applying to (43) the change of variable,

$$R = r^2 \Rightarrow dR = 2r\,dr,\; r = \sqrt{R},$$

we get

$$\begin{aligned} E\left[f(\theta,\tilde{Y},\tilde{Z}_m)\right] &= \int_{R=0}^{\infty}\frac{e^{-R}}{\pi^{3/2}}RS(\sqrt{R})\,\frac{dR}{2\sqrt{R}} \\ &= \frac{1}{2\pi^{3/2}}\int_{R=0}^{\infty} S(\sqrt{R})\,e^{-R}\sqrt{R}\,dR \\ &\approx \frac{1}{2\pi^{3/2}}\sum_{k=1}^{n_{GL}} w_{GL,k}\;S(\sqrt{x_{GL,k}}), \qquad (44) \end{aligned}$$

where (44) holds with weights and sample points obtained using the Gauss-Laguerre scheme, which can approximate integrals of the form $\int_0^{\infty} f(x)\sqrt{x}e^{-x}\;dx$ by $\sum_{i=1}^{N} w_i f(x_i)$ ([4], pg 130).

The function $S(\cdot)$ in (42), which is a surface integral, is approximated by choosing points (and corresponding weights) on the surface of the unit sphere. This technique of evaluating multi-dimensional surface integrals as a weighted sum of the function evaluated at points on the surface is called cubature, and has been applied for functions integrated against a Gaussian density [3]. That is, we approximate

$\int_{\mathbf{S}} f(\mathbf{x})dA(\mathbf{x})$ as $\sum_{i=1}^{N} w_i f(\mathbf{x_i})$, where for a d-dimensional surface $\mathbf{S}$, $\forall i, \mathbf{x_i} \in \mathbb{R}^d$. This scheme broadly implies

$$S(r) = \int_{U_3} f(H_1(H_2(r,\mathbf{u})))\; dA(\mathbf{u}) \approx \sum_{l=1}^{N_{Cub}} w_{Cub,l}\; f(H_1(H_2(r,\mathbf{x}_{Cub,l}))), \tag{45}$$

and substituting (45) into (43) yields

$$E\left[f(\theta,\tilde{Y},\tilde{Z}_m)\right] \approx \frac{1}{2\pi^{3/2}} \sum_{k=1}^{N_{GL}} w_{GL,k} \sum_{l=1}^{N_{Cub}} w_{Cub,l}\; (f \circ H_1 \circ H_2)(\sqrt{x_{GL,k}}, \mathbf{x}_{Cub,l}). \tag{46}$$

Equation (46) provides a numerical integration scheme for any function $f(\cdot)$ on $\mathbb{R}^3$ introduced in (37); we need to apply (46) to the function in (36). Before that, we write the CDF of $e^{\tilde{Z}_{m(1)}} + e^{\tilde{Z}_{m(2)}}$ in a format that is amenable to numerical computation (more specifically, we take the exponent of the logarithms in obtaining (48) from (47) because the a_{iZ_m} and a_{jY} terms can be very large), and – for ease of reference – we define all quantities in one place that are required to compute this CDF:

$$P(e^{\tilde{Z}_{m(1)}} + e^{\tilde{Z}_{m(2)}} < t)$$
$$= E\left\{\left[\prod_{n=1}^{10} \Phi\left(\frac{\tilde{Y} - (c_n\theta + \delta + \mu_3)}{\alpha}\right)\right] \sum_{\substack{i,j \\ i\neq j}}^{10} \frac{\Phi\left(\frac{\ln(t-e^{\tilde{Z}_m})^+ - \mu_{j|\tilde{Y}} - \delta - \mu_3}{\sqrt{\hat{\sigma}^2+\sigma_3^2}}\right) \bar{\Phi}\left(\frac{\tilde{Y} - \mu_{i|\tilde{Z}_m} - \delta - \mu_3}{\sqrt{\hat{\sigma}^2+\sigma_3^2}}\right) a_{iZ_m} a_{jY}}{\Phi\left(\frac{\tilde{Y}-(c_i\theta+\delta+\mu_3)}{\alpha}\right) \Phi\left(\frac{\tilde{Y}-(c_j\theta+\delta+\mu_3)}{\alpha}\right)}\right\}, \tag{47}$$

$$=E\left\{\sum_{\substack{i,j \\ i\neq j}}^{10} \eta_j \xi_i \exp\left[\left(\sum_{n=1}^{10} \ln \Phi_n\right) + \ln a_{iZ_m} + \ln a_{jY} - \ln \Phi_i - \ln \Phi_j\right]\right\}, \tag{48}$$

where

$$
\begin{aligned}
&\xi_i \triangleq \bar{\Phi}\left(\frac{\tilde{Y}-\mu_{i|\tilde{Z}_m}-\delta-\mu_3}{\sqrt{\hat{\sigma}^2+\sigma_3^2}}\right), && \eta_j \triangleq \Phi\left(\frac{\ln(t-e^{\tilde{Z}_m})^+ - \mu_{j|\tilde{Y}}-\delta-\mu_3}{\sqrt{\hat{\sigma}^2+\sigma_3^2}}\right),\\
&\Phi_n \triangleq \Phi\left(\frac{\tilde{Y}-(c_n\theta+\delta+\mu_3)}{\sqrt{\sigma^2+\sigma_3^2}}\right), && \mu_{j|\tilde{Y}} \triangleq \frac{c_j\theta/\sigma^2+(\tilde{Y}-\delta-\mu_3)/\sigma_3^2}{1/\sigma^2+1/\sigma_3^2},\\
&\hat{\sigma}^2 \triangleq \frac{1}{1/\sigma^2+1/\sigma_3^2}, && \mu_{i|\tilde{Z}_m} \triangleq \frac{c_i\theta/\sigma^2+(\tilde{Z}_m-\delta-\mu_3)/\sigma_3^2}{1/\sigma^2+1/\sigma_3^2},\\
&\alpha^2 \triangleq \sigma^2+\sigma_3^2,\\
&\tilde{Y}\sim\mathcal{N}(\bar{c}\theta+\delta+\mu_3,\sigma^2+\sigma_3^2), && \ln a_{jY} \triangleq -\frac{(c_j-\bar{c})\theta\left[(c_j-\bar{c})\theta-2\left(\tilde{Y}-(\bar{c}\theta+\delta+\mu_3)\right)\right]}{2(\sigma^2+\sigma_3^2)},\\
&\tilde{Z}_m\sim\mathcal{N}(\bar{c}\theta+\delta+\mu_3,\sigma^2+\sigma_3^2), && \ln a_{iZ_m} \triangleq -\frac{(c_i-\bar{c})\theta\left[(c_i-\bar{c})\theta-2\left(\tilde{Z}_m-(\bar{c}\theta+\delta+\mu_3)\right)\right]}{2(\sigma^2+\sigma_3^2)}.
\end{aligned}
\tag{49}
$$

Applying (46) to (48), we get

$$
\begin{aligned}
&P(e^{\tilde{Z}_{m(1)}}+e^{\tilde{Z}_{m(2)}}<t)\\
\approx&\frac{1}{2\pi^{3/2}}\sum_{k=1}^{N_{GL}} w_{GL,k}\sum_{l=1}^{N_{Cub}} w_{Cub,l}\sum_{\substack{i,j\\ i\neq j}}^{10}\eta_j^{kl}\xi_i^{kl}\exp\left[\left(\sum_{n=1}^{10}\ln\Phi_n^{kl}\right)+\ln a_{iZ_m}^{kl}+\ln a_{jY}^{kl}-\ln\Phi_i^{kl}-\ln\Phi_j^{kl}\right],
\end{aligned}
\tag{50}
$$

where the superscript kl is used to emphasize that the quantity depends on k and l through $(\theta^{kl}, \tilde{Y}^{kl}, \tilde{Z}_m^{kl})$. Recall that $(\theta^{kl}, \tilde{Y}^{kl}, \tilde{Z}_m^{kl}) = (H_1 \circ H_2)(\sqrt{x_{GL,k}}, \mathbf{x}_{Cub,l})$, which upon expanding gives

$$
\begin{aligned}
\theta^{kl} &= \mu+\sqrt{2}\tau\sqrt{x_{GL,k}}\,x_{Cub,l1},\\
\tilde{Y}^{kl} &= \bar{c}\theta^{kl}+\delta+\mu_3+\sqrt{2}\alpha\,\sqrt{x_{GL,k}}\,x_{Cub,l2},\\
\tilde{Z}_m^{kl} &= \bar{c}\theta^{kl}+\delta+\mu_3+\sqrt{2}\alpha\,\sqrt{x_{GL,k}}\,x_{Cub,l3}.
\end{aligned}
\tag{51}
$$

In summary, we calculate the fusion probability by using (50), with all its variables evaluated using (49) at the values of triplet $(\theta^{kl}, \tilde{Y}^{kl}, \tilde{Z}_m^{kl})$ described by (51).

1.7 Solving Problems (3)-(4) and (5)-(7)

In this section, we discuss the computational details of solving (3)-(4) and (5)-(7), which include casting (3)-(4) as a simple linear regression problem, reducing (5)-(7) to an unconstrained optimization

problem by solving constraints (6)-(7) for the thresholds t_{1k} and t_{2k}, and constructing an initial solution for the optimization routine in (5)-(7).

Recalling that $\bar{G}_1(t)$ is the complementary CDF of a lognormal with parameters (μ_G, σ_G^2), we see that the left side of (4) is equal to $\bar{G}_1(t_l) = \bar{\Phi}\Big(\frac{\ln t_l - \mu_G}{\sigma_G}\Big)$. Equating this expression to FAR_l yields

$$\ln t_l = \mu_G + \sigma_G \bar{\Phi}^{-1}(\mathrm{FAR}_l) \quad \text{for} \quad l = 3, 4, 5, 6, \tag{52}$$

where the values of parameters μ_G and σ_G need to be estimated. Thus (52) provides theoretical estimates of the log thresholds, and because we already know the observed log thresholds $\ln \hat{t}_l$, we use linear regression to solve (3) and obtain estimates of μ_G and σ_G.

Given μ_G and σ_G, we can transform problem (5)-(7) into an unconstrained optimization problem. Constraint (6) can be rearranged as

$$t_{1k} = e^{\mu_G + \sigma_G \bar{\Phi}^{-1}(\mathrm{FAR}_{1k})} \;\; \text{for} \;\; k = 1, \ldots, 7. \tag{53}$$

However, because there is no simple expression for the sum of two iid lognormals, it is hard to directly solve for t_{2k} in constraint (7). The approximation in [8] is not sufficiently accurate for our purposes, and so we use simulation to generate 10^6 imposter similarity scores $\{G_i^{(1)}, G_i^{(2)}\}_{i=1}^{10^6}$, and for each k let t_{2k} be the 1-FAR_{2k} quantile of the empirical distribution of $\{G_i^{(1)} + G_i^{(2)}\}_{i=1}^{10^6}$. This procedure yields $t_{2k} = (54.60, 53.68, 50.80, 48.37, 46.01, 43.83, 42.42)$ for $k = 1, \ldots, 7$. Plugging these estimates of t_{1k} and t_{2k} into the objective function, we solve (5) as an unconstrained optimization problem

$$\min_{\mu,\tau,\sigma,\delta,s} \sum_{k=1}^{7} \left(\frac{F_1(t_{1k}) - \mathrm{FRR}_{1k}}{\mathrm{FRR}_{1k}} \right)^2 + \sum_{k=1}^{7} \left(\frac{F_{1m}(t_{1k}) - \mathrm{FRR}_{1mk}}{\mathrm{FRR}_{1mk}} \right)^2 + \sum_{k=1}^{7} \left(\frac{F_{2m}(t_{2k}) - \mathrm{FRR}_{2mk}}{\mathrm{FRR}_{2mk}} \right)^2. \tag{54}$$

Finally, note that solving the optimization problem requires an initial solution $(\mu_0, \tau_0, \sigma_0, \delta_0, s_0)$. As an initial guess, because the similarity scores are normalized to a 100-point scale, we set the lognormal median, e^{μ_0}, equal to 50 to obtain $\mu_0 = \ln 50$. For (τ_0, σ_0, s_0), we use the estimates of (τ, σ, s) from the solution of (1)-(2). As noted before, although the experimental setup of (1)-(2) used to estimate $(c_1, \ldots, c_{10})$ differs from that used to estimate all other parameters, we expect these values to provide a good starting point. Also, recall from §1.3 that the solution of (1)-(2) provides no information about δ. We expect δ to be negative but close to zero because it should be much smaller than μ. Therefore, we

set δ_0 equal to 0. Finally, the entire stage 1 and stage 2 procedure is repeated multiple times, with each run (except the first) using the optimized values from the previous run as the inital solution.

2 Iris Parameter Estimation

The data are described in §2.1, mathematical expressions for FRR and FAR are derived in §2.2 and the iris parameters are estimated in §2.3.

2.1 Data

The data for our iris parameters are taken from [9]. Although single- and dual-eye cameras are tested in [9], we restrict ourselves to the dual-eye experiments because they achieved better performance and smaller delays than the single-eye experiments. The relevant data are in Table 8 and Fig. 13 of [9]: Fig. 13 gives four points on the FRR vs. FAR curve, denoted by (FRR_{2j},FAR_{2j}), $j = 1, \ldots, 4$, which consider two attempts of two (left and right) irises, and Table 8 fixes $FAR_1 = 10^{-6}$ and provides the value FRR_1 for one attempt of both irises. When multiple attempts are taken of an iris, the maximum similarity score across attempts is used. In addition, the fusion of two irises is the maximum of the two similarity scores; i.e., although fingers are fused with the sum, irises are fused with the maximum in the UIDAI experiments. Each attempt in [9] corresponds to using the first image that meets the quality threshold or the best among three images, whichever happens first. However, because this process is used during enrollment and during each verification, we do not attempt to model the details within an attempt. These experiments use four types of dual-eye cameras, and then discard one type as not being good, and hence report results on the average of three types of good cameras. For two attempts of two fingers (i.e., Fig. 13 of [9]), we also know that for $l = 4, 5, 6$, the threshold values $\hat{t}_4 = 27$, $\hat{t}_5 = 36$ and $\hat{t}_6 = 46$ generate the FAR values $FAR_l = 10^{-l}$.

These experiments exclude the 0.33% of people whose iris images could not be acquired, defined as having the image not be usable for at least four of the eight single-eye and dual-eye cameras tested [9]. As in the fingerprint case, we consider two scenarios: the 0.33% are omitted and the five FRR values are used directly in the exclusion case, and in the inclusion scenario we assume a failure-to-acquire rate of 0.0033 and use the relationships FRR=FTA+FNMR(1-FTA) and FAR=FMR(1-FTA).

2.2 Analysis

We now derive expressions for FRR_1, FAR_1, FRR_{2j} and FAR_{2j}. By construction, we know that for genuine scores,

$$\begin{pmatrix} \tilde{Z}_{11} \\ \tilde{Z}_{12} \end{pmatrix} = \begin{pmatrix} \tilde{X}_{11} + \tilde{\gamma}_{11} \\ \tilde{X}_{12} + \tilde{\gamma}_{12} \end{pmatrix} \sim N\left(\begin{pmatrix} \mu_{11} + \psi \\ \mu_{11} + \psi \end{pmatrix}, \begin{pmatrix} \sigma_{11}^2 + \beta^2 & \rho\sigma_{11}^2 \\ \rho\sigma_{11}^2 & \sigma_{11}^2 + \beta^2 \end{pmatrix}\right). \tag{55}$$

Because iris fusion is performed via the maximum, it follows that for the unknown threshold t_1 used to produce (FRR_1,FAR_1), FRR_1 is

$$F_{1I}(t_1) = P(Z_{11} < t_1, Z_{12} < t_1) = \Phi_2\left(\frac{\ln t_1 - \mu_{11} - \psi}{\sqrt{\sigma_{11}^2 + \beta^2}}, \frac{\ln t_1 - \mu_{11} - \psi}{\sqrt{\sigma_{11}^2 + \beta^2}}; \frac{\sigma_{11}^2\rho}{\sigma_{11}^2 + \beta^2}\right), \tag{56}$$

where $\Phi_2\,(x, y; \rho)$ is the bivariate CDF of two standard normal variables with correlation coefficient ρ. The independence of the imposter similarity scores for the left and right irises implies that

$$\text{FAR}_1 = 1 - \Phi^2\left(\frac{\ln t_1 - \mu_{GI}}{\sigma_{GI}}\right). \tag{57}$$

When there are two attempts of each iris, we let $\tilde{Z}_{im} = \tilde{X}_i + \max_{k=1,2}\tilde{\gamma}_{ik}$ and let t_{2j} be the unknown threshold corresponding to (FRR_{2j},FAR_{2j}) for $j = 1, \ldots, 4$. We use Clark's method to approximate $\max_{k=1,2}\tilde{\gamma}_{ik}$ as a normal random variable with mean $\psi_2 \triangleq \psi + \frac{1}{\sqrt{\pi}}\beta$ and variance $\tilde{\beta}^2 \triangleq \frac{\pi-1}{\pi}\beta^2$, which implies that FRR_{2j} is

$$F_{2I}(t_{2j}) = P(Z_{11,m} < t_{2j}, Z_{12,m} < t_{2j}) = \Phi_2\left(\frac{\ln t_{2j} - \mu_{11} - \psi_2}{\sqrt{\sigma_{11}^2 + \tilde{\beta}^2}}, \frac{\ln t_{2j} - \mu_{11} - \psi_2}{\sqrt{\sigma_{11}^2 + \tilde{\beta}^2}}; \frac{\sigma_{11}^2\rho}{\sigma_{11}^2 + \tilde{\beta}^2}\right). \tag{58}$$

Because we ignore measurement noise, the FAR_{2j} values are also given by (57):

$$\text{FAR}_{2j} = 1 - \Phi^2\left(\frac{\ln t_{2j} - \mu_{GI}}{\sigma_{GI}}\right) \quad \text{for} \quad j = 1, \ldots, 4. \tag{59}$$

2.3 Parameter Estimation

We have seven parameters to estimate: $\mu_{11}, \sigma_{11}, \rho, \psi, \beta, \mu_{GI}, \sigma_{GI}$. The parameters μ_{11} and ψ appear in equations (56) and (58) as $\mu_{11} + \psi$, and hence cannot be individually determined. We arbitrarily assume

$\psi = 0$, leaving us with six parameters. We initially estimate the imposter parameters μ_{GI} and σ_{GI} using the three threshold values for three specific FAR values, which yields a negative mean score ($\mu_{GI} = -1.23$, $\sigma_{GI} = 0.53$), which in turn generate extremely large estimates of μ_{11}, σ_{11} and b. Consequently, we also estimate μ_{GI} and σ_{GI} from Hamming distance data in [10], where the best camera has a Hamming distance distribution with mean 0.456 and standard deviation 0.0214. Assuming that the similarity score equals 100 times 1 minus the Hamming distance, we obtain $\mu_{GI} = 4.00$ and $\sigma_{GI} = 0.039$. Although both pairs of estimates lead to comparable fits to the FRR vs. FAR curves, and we are only interested in the right tail of the imposter distribution (and the left tail of the genuine distribution), we nonetheless use the latter approach so that the parameter values make more intuitive sense.

Solving equations (57) and (59) for the thresholds $t_1 = \exp(\mu_{GI} + \sigma_{GI}\Phi^{-1}(\sqrt{1 - FAR_1}))$ and $t_{2j} = \exp(\mu_{GI} + \sigma_{GI}\Phi^{-1}(\sqrt{1 - FAR_{2j}}))$, and substituting these thresholds into (56) and (58) yield our least squares problem for the remaining four parameters:

$$\min_{\mu_{11},\sigma_{11},\rho,\beta} \left(\frac{F_{1I}(\mu_{GI} + \sigma_{GI}\Phi^{-1}(\sqrt{1 - FAR_1})) - FRR_1}{FRR_1} \right)^2 + \sum_{j=1}^{4} \left(\frac{F_{2I}(\mu_{GI} + \sigma_{GI}\Phi^{-1}(\sqrt{1 - FAR_{2j}})) - FRR_{2j}}{FRR_{2j}} \right)^2 . \tag{60}$$

However, the solution to (60) turns out to be indeterminable: many combinations of σ_{11}, β, ρ can give the same result. Fig. 6 of [11] allows us to roughly estimate ρ to be 0.6, and solving (60) for the three remaining parameters leads to our final estimates (the optimal solution is very insensitive to 1000 randomly-generated starting points).

3 Proposed Policies

We consider single-stage policies in §3.1 and two-stage policies in §3.2.

3.1 Single-stage Policies

A single-stage policy takes as input a resident's observed log similarity scores during BFD and BID, $\tilde{Y} = (\tilde{Y}_1, \ldots, \tilde{Y}_{12})$, and chooses to acquire a subset S of the 10 fingers, along with either neither or both irises (due to the use of a dual-eye camera). We then observe the new similarity scores $\tilde{Z}$ for the acquired subset and make an accept/reject decision (i.e., deem that the resident is genuine or an imposter). Our

overall goal is to minimize the FRR subject to constraints on the FAR and average delay.

Because this problem is difficult to solve, we make several simplifying assumptions. First, we solve this problem for each individual resident because we cannot easily compute the expectation over the distribution of $(\tilde{Y}, \tilde{Z})$. In particular, we require that each resident's FAR is equal to a specified value p. Second, it is much easier to consider a delay penalty than a delay constraint, and so we add the delay penalty $\tilde{\lambda}(c_F|S| + c_I I_{11})$, where $\tilde{\lambda}$ is the Lagrange multiplier associated with the delay constraint, $|S|$ is the number of fingers acquired, I_{11} is the indicator function for acquiring the irises, c_I is the variable delay for the irises and c_F is the variable delay per finger. With these simplifications, our problem can be formulated as

$$\min_{S\subseteq\{1,\dots,10\},I_{11},t} \quad \text{FRR} + \tilde{\lambda}(c_F|S| + c_I I_{11}) \tag{61}$$

$$\text{subject to} \quad \text{FAR} = p. \tag{62}$$

Overview. We describe our approach to analyzing (61)-(62) in five steps. Let H_0 denote the null hypothesis that the resident is an imposter and H_1 be the alternative hypothesis that the resident is genuine. In step 1, we follow [13] and construct the likelihood ratio $L = \frac{f_{H_1}(\tilde{Z})}{f_{H_0}(\tilde{Z})}$, where $f_{H_1}(\tilde{Z})$ and $f_{H_0}(\tilde{Z})$ are the PDFs of the observed log similarity scores $\tilde{Z}$ under the respective hypotheses. The Neyman-Pearson lemma states that the form of the optimal policy is to accept the resident as genuine (i.e., reject H_0) if $L > t$ for some threshold t and reject the resident (i.e., accept H_0) if $L < t$. In step 2, we solve for the threshold t by equating the FAR, which is $P(L > t|H_0)$, to the pre-specified value p. In step 3, we calculate the FRR, which is $P(L < t|H_1)$. In step 4, we observe that the 10 fingers can be ranked according to a simple rule, which greatly simplifies the analysis. In step 5, we solve (61)-(62).

The Likelihood Ratio. Until step 4, we assume that the $|S|$ fingers acquired are indexed by $i = 1, \dots, |S|$, so that $\tilde{Z} = (\tilde{Z}_1, \dots, \tilde{Z}_{|S|})$ if $I_{11} = 0$ and $\tilde{Z} = (\tilde{Z}_1, \dots, \tilde{Z}_{|S|}, \tilde{Z}_{11}, \tilde{Z}_{12})$ if $I_{11} = 1$. Due to the independence of fingerprints and iris, we perform four analyses to obtain the likelihood ratio: under hypotheses H_0 and H_1, we find the conditional distribution of $(\tilde{Z}_1, \dots, \tilde{Z}_{|S|})$ given $(\tilde{Y}_1, \dots, \tilde{Y}_{10})$ and the conditional distribution of $(\tilde{Z}_{11}, \tilde{Z}_{12})$ given $(\tilde{Y}_{11}, \tilde{Y}_{12})$.

Under hypothesis H_1 that the resident is genuine, Prop. 3.13 in [5] implies that the distribution of θ

conditioned on observing the BFD scores $(\tilde{Y}_1, \ldots, \tilde{Y}_{10})$ is $\theta_{|\tilde{Y}} \sim \mathcal{N}(\mu_\theta, \sigma_\theta^2)$, where

$$\mu_\theta = \frac{\mu/\tau^2 + \sum_{i=1}^{10} (\tilde{Y}_i - \delta - \mu_3) c_i/(\sigma^2 + \sigma_3^2)}{1/\tau^2 + \sum_{i=1}^{10} c_i^2/(\sigma^2 + \sigma_3^2)}, \tag{63}$$

$$\sigma_\theta^2 \triangleq \frac{1}{1/\tau^2 + \sum_{i=1}^{10} c_i^2/(\sigma^2 + \sigma_3^2)}. \tag{64}$$

Because $\sigma_\theta \ll \mu_\theta$ for our parameter values (e.g., for the exclusion scenario, $\mu_\theta \approx 4.1$ depending on the $\tilde{Y}_i$ values, and $\sigma_\theta = 0.36$), we approximate the distribution $\theta_{|\tilde{Y}}$ by its mean μ_θ. Under H_1, this approximation along with (20)-(21) imply that $\tilde{Z}_i \sim \mathcal{N}(\mu_i + \delta, \hat{\sigma}^2 + s^2)$ are conditionally independent for $i = 1, \ldots, |S|$, where

$$\mu_i = \frac{c_i \mu_\theta/\sigma^2 + (\tilde{Y}_i - \delta - \mu_3)/\sigma_3^2}{1/\sigma^2 + 1/\sigma_3^2}. \tag{65}$$

To find the conditional distribution of $(\tilde{Z}_{11}, \tilde{Z}_{12})$ given $(\tilde{Y}_{11}, \tilde{Y}_{12})$ under hypothesis H_1, we first require the joint distribution of $(\tilde{X}_{11}, \tilde{X}_{12}, \tilde{Y}_{11}, \tilde{Y}_{12})$. Let us define

$$\mu_X \triangleq \begin{pmatrix} \mu_{11} \\ \mu_{11} \end{pmatrix}, \quad \Sigma_{XX} \triangleq \sigma_{11}^2 \begin{pmatrix} 1 & \rho \\ \rho & 1 \end{pmatrix}, \tag{66}$$

$$\mu_Y \triangleq \begin{pmatrix} \mu_{11} + \psi \\ \mu_{11} + \psi \end{pmatrix}, \quad \Sigma_{YY} \triangleq \begin{pmatrix} \sigma_{11}^2 + \beta^2 & \rho\sigma_{11}^2 \\ \rho\sigma_{11}^2 & \sigma_{11}^2 + \beta^2 \end{pmatrix}. \tag{67}$$

Recalling that $(\tilde{Y}_{11}, \tilde{Y}_{12})$ and $(\tilde{Z}_{11}, \tilde{Z}_{12})$ are identically distributed, we find that (55) implies

$$\begin{pmatrix} \tilde{X}_{11} \\ \tilde{X}_{12} \\ \tilde{Y}_{11} \\ \tilde{Y}_{12} \end{pmatrix} \sim \mathcal{N}\left(\begin{pmatrix} \mu_X \\ \mu_Y \end{pmatrix}, \begin{pmatrix} \Sigma_{XX} & \Sigma_{XX} \\ \Sigma_{XX} & \Sigma_{YY} \end{pmatrix} \right), \tag{68}$$

after noting that $\text{cov}(\tilde{X}_{12}, \tilde{Y}_{12})$=$\text{cov}(\tilde{X}_{11}, \tilde{Y}_{11})$=$\text{var}(\tilde{X}_{11}) = \sigma_{11}^2$ and $\text{cov}(\tilde{X}_{12}, \tilde{Y}_{11})$=$\text{cov}(\tilde{X}_{11}, \tilde{Y}_{12})$ =$\text{cov}(\tilde{X}_{11}, \tilde{X}_{12}) = \rho\sigma_{11}^2$. Using (68) and Prop. 3.13 in [5], we find that $(\tilde{X}_{11}, \tilde{X}_{12})$ given $(\tilde{Y}_{11}, \tilde{Y}_{12})$ is distributed as

$\mathcal{N}\left(\mu_{X|Y}, \Sigma_{X|Y}\right)$, where

$$\begin{aligned}
\mu_{X|Y} &\triangleq \mu_X + \Sigma_{XX}\Sigma_{YY}^{-1}\left(\begin{pmatrix}\tilde{Y}_{11}\\ \tilde{Y}_{12}\end{pmatrix} - \mu_Y\right),\\
&= \mu_X + \sigma_{11}^2\begin{pmatrix}1 & \rho\\ \rho & 1\end{pmatrix}\frac{1}{det(\Sigma_{YY})}\begin{pmatrix}\sigma_{11}^2+\beta^2 & -\rho\sigma_{11}^2\\ -\rho\sigma_{11}^2 & \sigma_{11}^2+\beta^2\end{pmatrix}\begin{pmatrix}\tilde{Y}_{11}-(\mu_{11}+\psi)\\ \tilde{Y}_{12}-(\mu_{11}+\psi)\end{pmatrix},\\
&= \mu_X + \frac{\sigma_{11}^2}{(\sigma_{11}^2+\beta^2)^2-\rho^2\sigma_{11}^4}\begin{pmatrix}1 & \rho\\ \rho & 1\end{pmatrix}\begin{pmatrix}\sigma_{11}^2+\beta^2 & -\rho\sigma_{11}^2\\ -\rho\sigma_{11}^2 & \sigma_{11}^2+\beta^2\end{pmatrix}\begin{pmatrix}\tilde{Y}_{11}-(\mu_{11}+\psi)\\ \tilde{Y}_{12}-(\mu_{11}+\psi),\end{pmatrix}\\
&= \begin{pmatrix}\mu_{11}\\ \mu_{11}\end{pmatrix} + \frac{\sigma_{11}^2}{(\sigma_{11}^2+\beta^2)^2-\rho^2\sigma_{11}^4}\begin{pmatrix}(1-\rho^2)\sigma_{11}^2+\beta^2 & \rho\beta^2\\ \rho\beta^2 & (1-\rho^2)\sigma_{11}^2+\beta^2\end{pmatrix}\begin{pmatrix}\tilde{Y}_{11}-(\mu_{11}+\psi)\\ \tilde{Y}_{12}-(\mu_{11}+\psi)\end{pmatrix},
\end{aligned} \tag{69}$$

and

$$\begin{aligned}
\Sigma_{X|Y} &\triangleq \Sigma_{XX} - \Sigma_{XX}\Sigma_{YY}^{-1}\Sigma_{XX},\\
&= \Sigma_{XX}(I - \Sigma_{YY}^{-1}\Sigma_{XX}),\\
&= \Sigma_{XX}\left[I - \frac{\sigma_{11}^2}{(\sigma_{11}^2+\beta^2)^2-\rho^2\sigma_{11}^4}\begin{pmatrix}\sigma_{11}^2+\beta^2 & -\rho\sigma_{11}^2\\ -\rho\sigma_{11}^2 & \sigma_{11}^2+\beta^2\end{pmatrix}\begin{pmatrix}1 & \rho\\ \rho & 1\end{pmatrix}\right],\\
&= \Sigma_{XX}\left[I - \frac{\sigma_{11}^2}{(\sigma_{11}^2+\beta^2)^2-\rho^2\sigma_{11}^4}\begin{pmatrix}(1-\rho^2)\sigma_{11}^2+\beta^2 & \rho\beta^2\\ \rho\beta^2 & (1-\rho^2)\sigma_{11}^2+\beta^2\end{pmatrix}\right],\\
&= \sigma_{11}^2\begin{pmatrix}1 & \rho\\ \rho & 1\end{pmatrix} - \frac{\sigma_{11}^4}{(\sigma_{11}^2+\beta^2)^2-\rho^2\sigma_{11}^4}\begin{pmatrix}(1-\rho^2)\sigma_{11}^2+(1+\rho^2)\beta^2 & \rho[(1-\rho^2)\sigma_{11}^2+2\beta^2]\\ \rho[(1-\rho^2)\sigma_{11}^2+2\beta^2] & (1-\rho^2)\sigma_{11}^2+(1+\rho^2)\beta^2\end{pmatrix}.
\end{aligned} \tag{70}$$

It follows by (55) and (69) that $(\tilde{Z}_{11}, \tilde{Z}_{12})$ given $(\tilde{Y}_{11}, \tilde{Y}_{12})$ is distributed as $\mathcal{N}(\mu_I, \Sigma_I)$, where

$$\mu_I \triangleq \mu_{X|Y} + \begin{pmatrix}\psi\\ \psi\end{pmatrix}, \quad \Sigma_I \triangleq \Sigma_{X|Y} + \beta^2 I. \tag{71}$$

Substituting from (69) and simplifying, we get

$$\mu_I = \begin{pmatrix} w_1\tilde{Y}_{11} + w_2\tilde{Y}_{12} + (1 - w_1 - w_2)(\mu_{11} + \psi) \\ w_1\tilde{Y}_{12} + w_2\tilde{Y}_{11} + (1 - w_1 - w_2)(\mu_{11} + \psi) \end{pmatrix}, \tag{72}$$

where

$$w_1 \triangleq \frac{(1-\rho^2)\sigma_{11}^4 + \sigma_{11}^2\beta^2}{(\sigma_{11}^2 + \beta^2)^2 - \rho^2\sigma_{11}^4}, \quad w_2 \triangleq \frac{\rho\sigma_{11}^2\beta^2}{\sigma_Z^4 - \rho^2\sigma_{11}^4}. \tag{73}$$

Similarly, substituting from (70) and simplifying, we get

$$\Sigma_I = \sigma_I^2 \begin{pmatrix} 1 & \rho_I \\ \rho_I & 1 \end{pmatrix}, \tag{74}$$

where

$$\sigma_I^2 \triangleq \frac{\beta^2[\beta^4 + 2(1-\rho^2)\sigma_{11}^4 + 3\sigma_{11}^2\beta^2]}{(\sigma_{11}^2 + \beta^2)^2 - \rho^2\sigma_{11}^4}, \quad \rho_I \triangleq \frac{\rho\sigma_{11}^2\beta^2}{\beta^4 + 2(1-\rho^2)\sigma_{11}^4 + 3\sigma_{11}^2\beta^2}. \tag{75}$$

Let $\tilde{Z}_I = (\tilde{Z}_{11}, \tilde{Z}_{12})^T$ and define the centered variables $\tilde{Z}_I^c = \tilde{Z}_I - \mu_I$. It follows that the numerator of the likelihood ratio L is given by

$$f_{H_1}(\tilde{Z}) = \left(\prod_{i=1}^{|S|} \frac{1}{\sqrt{2\pi(\hat{\sigma}^2 + s^2)}} e^{-(\tilde{Z}_i - \mu_i - \delta)^2/2(\hat{\sigma}^2+s^2)} \right) \left(\prod_{j=1}^{I_{11}} \frac{1}{2\pi\sigma_I^2\sqrt{1-\rho_I^2}} \exp\left\{ -\frac{(\tilde{Z}_I^c)^T\Sigma_I^{-1}\tilde{Z}_I^c}{2} \right\} \right), \tag{76}$$

where (here and elsewhere) we use the convention that the empty product equals 1.

Let $e^T = (1\ 1)$ and I be the 2×2 identity matrix. Under hypothesis H_0 that the resident is an imposter, we have that $\tilde{Z}_i$ are iid $\mathcal{N}(\mu_G, \sigma_G^2)$ for $i = 1, \ldots, |S|$ and $\tilde{Z}_I \sim \mathcal{N}(e\mu_{GI}, \Sigma_{GI})$, where $\Sigma_{GI} \triangleq \sigma_{GI}^2 I$. Define the centered variables $\tilde{Z}_{GI}^c = \tilde{Z}_I - e\mu_{GI}$. It follows that the denominator of the likelihood ratio is

$$f_{H_0}(\tilde{Z}) = \left(\prod_{i=1}^{|S|} \frac{1}{\sqrt{2\pi}\sigma_G} e^{-(\tilde{Z}_i - \mu_G)^2/2\sigma_G^2} \right) \left(\prod_{j=1}^{I_{11}} \frac{1}{2\pi\sigma_{GI}^2} \exp\left\{ -\frac{(\tilde{Z}_{GI}^c)^T\Sigma_{GI}^{-1}\tilde{Z}_{GI}^c}{2} \right\} \right). \tag{77}$$

Define the vector $\bar{\mu} \triangleq \mu_I - \mu_{GI}$, so that $\tilde{Z}_{GI}^c = \tilde{Z}_I^c + \bar{\mu}$. By (76)-(77), the log likelihood ratio (i.e.,

$\ln L$) is given by

$$|S|\ln\omega_1+\sum_{i=1}^{|S|}\left[\frac{(\tilde{Z}_i-\mu_G)^2}{2\sigma_G^2}-\frac{(\tilde{Z}_i-\mu_i-\delta)^2}{2(\hat{\sigma}^2+s^2)}\right]+I_{11}\left(\omega_2-\frac{(\tilde{Z}_I^c)^T\Sigma_I^{-1}\tilde{Z}_I^c}{2}+\frac{(\tilde{Z}_I^c+\bar{\mu})^T\Sigma_{GI}^{-1}(\tilde{Z}_I^c+\bar{\mu})}{2}\right), \tag{78}$$

where

$$\omega_1 \triangleq \frac{\sigma_G}{\sqrt{\hat{\sigma}^2+s^2}}, \tag{79}$$

$$\omega_2 \triangleq \ln\left[\frac{\sigma_{GI}^2}{\sigma_I^2\sqrt{1-\rho_I^2}}\right]. \tag{80}$$

Choosing the Threshold t. The optimal decision rule dictates that we declare the resident to be genuine if and only if $L > t$. We need the threshold t to satisfy FAR $= P(L > t|H_0) = p$, which by (78) can be written as

$$\begin{aligned}p =P\Bigg(&|S|\ln\omega_1+\sum_{i=1}^{|S|}\left[\frac{(\tilde{Z}_i-\mu_G)^2}{2\sigma_G^2}-\frac{(\tilde{Z}_i-\mu_i-\delta)^2}{2(\hat{\sigma}^2+s^2)}\right]\\ &+I_{11}\left(\omega_2-\frac{(\tilde{Z}_I^c)^T\Sigma_I^{-1}\tilde{Z}_I^c}{2}+\frac{(\tilde{Z}_I^c+\bar{\mu})^T\Sigma_{GI}^{-1}(\tilde{Z}_I^c+\bar{\mu})}{2}\right)>\ln t \mid H_0\Bigg).\end{aligned} \tag{81}$$

Recall from (77) that under H_0, $\tilde{Z}_i \sim \mathcal{N}(\mu_G, \sigma_G^2)$ and iid for $i = 1, \ldots, |S|$. Letting $N_i \triangleq (\tilde{Z}_i - \mu_G)/\sigma_G \sim \mathcal{N}(0, 1)$ and simplifying the ith term inside the summation in (81), we have

$$\frac{(\tilde{Z}_i-\mu_G)^2}{2\sigma_G^2}-\frac{(\tilde{Z}_i-\mu_i-\delta)^2}{2(\hat{\sigma}^2+s^2)}=\frac{1-\omega_1^2}{2}\left(N_i+\frac{\omega_1^2}{1-\omega_1^2}\beta_i\right)^2-\frac{\omega_1^2\beta_i^2}{2(1-\omega_1^2)}, \tag{82}$$

where

$$\beta_i \triangleq \frac{\mu_i+\delta-\mu_G}{\sigma_G} \quad \text{for} \quad i=1,\ldots,|S|. \tag{83}$$

Turning to the irises part of (81), we use the fact that the distribution of a definite quadratic form of Gaussian variables, x^TQx where $Q \succ 0$ and $x \sim \mathcal{N}_r(\mu, \Sigma)$, can be expressed as a positive linear combination of independent non-central chi square random variables [14]. More specifically,

$$x^TQx \overset{d}{=} \sum_{i=1}^{r}\lambda_i y_i^2, \tag{84}$$

where $y \sim \mathcal{N}_r(P^TA^{-1}\mu, I)$ are independent, A is defined by Cholesky's decomposition of $\Sigma = AA^T$, and

$\lambda = (\lambda_1, \ldots, \lambda_r) > 0$ and the orthogonal matrix P are the eigenvalues and eigenvectors of $A^T QA$. To be able to apply this technique to the irises part of (81), we must satisfy

$$(\tilde{Z}_I^c + \bar{\mu})^T \Sigma_{GI}^{-1} (\tilde{Z}_I^c + \bar{\mu}) - (\tilde{Z}_I^c)^T \Sigma_I^{-1} \tilde{Z}_I^c = (\tilde{Z}_I^c + \gamma_1)^T Q_1 (\tilde{Z}_I^c + \gamma_1) + K_1 \quad \text{under} \quad H_1, \tag{85}$$

$$= (\tilde{Z}_{GI}^c + \gamma_0)^T Q_0 (\tilde{Z}_{GI}^c + \gamma_0) + K_0 \quad \text{under} \quad H_0, \tag{86}$$

for some vectors γ_1, γ_0, constants K_1, K_0, and definite matrices Q_1, Q_0. Note that if we can satisfy (85) for some γ_1, K_1, and Q_1, then (86) is easily satisfied for the choice of $\gamma_0 = \gamma_1 - \bar{\mu}, K_0 = K_1$, and $Q_0 = Q_1$. Thus, we concentrate on satisfying (85).

Expanding both sides of (85), we have

$$(\tilde{Z}_I^c)^T \left(\Sigma_{GI}^{-1} - \Sigma_I^{-1} \right) \tilde{Z}_I^c + 2\bar{\mu} e^T \Sigma_{GI}^{-1} \tilde{Z}_I^c + \bar{\mu}^2 e^T \Sigma_{GI}^{-1} e = (\tilde{Z}_I^c)^T Q_1 \tilde{Z}_I^c + 2\gamma_1 e^T Q_1 \tilde{Z}_I^c + \gamma_1^2 e^T Q_1 e + K_1,$$

and matching coefficients gives

$$Q_1 = \Sigma_{GI}^{-1} - \Sigma_I^{-1}, \tag{87}$$

$$\gamma_1 = [I - \Sigma_{GI} \Sigma_I^{-1}]^{-1} \bar{\mu}, \tag{88}$$

$$K_1 = \bar{\mu}^T [I - (I - \Sigma_I^{-1} \Sigma_{GI})^{-1}] \Sigma_{GI}^{-1} \bar{\mu}. \tag{89}$$

To explicitly express these quantities in terms of our parameters, we first note that $\Sigma_{GI}^{-1} = I/\sigma_{GI}^2$ and (74) implies

$$\Sigma_I^{-1} = \frac{1}{\sigma_I^2 (1 - \rho_I^2)} \begin{pmatrix} 1 & -\rho_I \\ -\rho_I & 1 \end{pmatrix}, \tag{90}$$

and hence

$$\Sigma_{GI}^{-1} - \Sigma_I^{-1} = \begin{pmatrix} \frac{1}{\sigma_{GI}^2} - \frac{1}{\sigma_I^2(1-\rho_I^2)} & \frac{\rho_I}{\sigma_I^2(1-\rho_I^2)} \\ \frac{\rho_I}{\sigma_I^2(1-\rho_I^2)} & \frac{1}{\sigma_{GI}^2} - \frac{1}{\sigma_I^2(1-\rho_I^2)} \end{pmatrix}, \tag{91}$$

$$e^T \Sigma_{GI}^{-1} e = \frac{2}{\sigma_{GI}^2}, \text{ and } e^T \Sigma_I^{-1} e = \frac{2}{\sigma_I^2 (1 + \rho_I)}. \tag{92}$$

Substituting (91)-(92) into (87), (88) and (89), and from our discussion below (86), we get

$$Q_1 = \begin{pmatrix} \frac{1}{\sigma_{GI}^2} - \frac{1}{\sigma_I^2(1-\rho_I^2)} & \frac{\rho_I}{\sigma_I^2(1-\rho_I^2)} \\ \frac{\rho_I}{\sigma_I^2(1-\rho_I^2)} & \frac{1}{\sigma_{GI}^2} - \frac{1}{\sigma_I^2(1-\rho_I^2)} \end{pmatrix} = Q_0,$$
$$\gamma_1 = \frac{\bar{\mu}}{1 - \frac{\sigma_{GI}^2}{\sigma_I^2(1-\rho_I^2)}}, \quad \gamma_0 = \frac{\bar{\mu}}{\frac{\sigma_I^2(1-\rho_I^2)}{\sigma_{GI}^2} - 1}, \tag{93}$$
$$K_1 = \frac{-2\bar{\mu}^2}{\sigma_Z^2(1-\rho_I^2) - \sigma_{GI}^2} = K_0.$$

Before applying this result, we check the conditions for Q_1 to be definite. Because its off-diagonal entries are positive, $-Q_1 \succ 0$ is ruled out. We can only have $Q_1 \succ 0$, which from (93) requires that

$$\frac{1}{\sigma_{GI}^2} > \frac{1}{\sigma_I^2(1-\rho_I^2)}, \text{ and} \tag{94}$$
$$\frac{1}{\sigma_{GI}^2} - \frac{1}{\sigma_I^2(1-\rho_I^2)} > \frac{\rho_I}{\sigma_I^2(1-\rho_I^2)} \iff \frac{1}{\sigma_{GI}^2} > \frac{1}{\sigma_I^2(1-\rho_I)}. \tag{95}$$

Because $0 < \rho_I < 1 \Rightarrow \frac{1}{1-\rho_I} > \frac{1}{1-\rho_I^2}$, (95) implies (94). Thus, the only condition we require for Q_1 to be (positive) definite is that

$$\sigma_I^2(1-\rho_I) > \sigma_{GI}^2, \tag{96}$$

which holds in our case.

Recall that under H_0, $\tilde{Z}_I \sim \mathcal{N}(e\mu_{GI}, \Sigma_{GI})$. We are now in a position to use (86) to express the irises part of (81) by

$$I_{11}\left(\omega_2 + \frac{(\tilde{Z}_{GI}^c + \gamma_0)^T Q_0 (\tilde{Z}_{GI}^c + \gamma_0) + K_0}{2}\right), \tag{97}$$

which by (84) equals

$$I_{11}\left(\omega_2 + \frac{K_0}{2} + \frac{\sum_{i=1}^2 \hat{\lambda}_i \hat{y}_i^2}{2}\right), \tag{98}$$

where, from the discussion below (84), we have that $\hat{y} = (\hat{y}_1 \;\; \hat{y}_2)^T \sim \mathcal{N}(\hat{\mu}_y, I)$ (where $\hat{\mu}_y$ is defined below), $\hat{A}$ is obtained using Cholesky's decomposition of $\Sigma_{GI} = \hat{A}\hat{A}^T$, and $\hat{\lambda} = (\hat{\lambda}_1 \;\; \hat{\lambda}_2)^T$ and $\hat{P}$ are the eigenvalues and eigenvectors of $\hat{A}^T Q_0 \hat{A}$. Explicitly evaluating these quantities, we have $\hat{A} = \sigma_{GI} I$, $\hat{P} = \frac{1}{\sqrt{2}}\begin{pmatrix} 1 & 1 \\ 1 & -1 \end{pmatrix}$,

and the final parameters of interest

$$\hat{\lambda}_1 = 1 - \frac{\sigma_{GI}^2}{\sigma_I^2(1+\rho_I)}, \tag{99}$$

$$\hat{\lambda}_2 = 1 - \frac{\sigma_{GI}^2}{\sigma_I^2(1-\rho_I)}, \tag{100}$$

$$\hat{\mu}_y = \begin{pmatrix} \hat{\mu}_{y1} \\ \hat{\mu}_{y2} \end{pmatrix} \triangleq \frac{1}{\sqrt{2}\sigma_{GI}} \begin{pmatrix} 1 & 1 \\ 1 & -1 \end{pmatrix} [(I - \Sigma_{GI}\Sigma_I^{-1})^{-1} - I]\bar{\mu}. \tag{101}$$

By (101) we can re-express (98) as

$$I_{11}\left(\omega_2 + \frac{K_0}{2} + \frac{\hat{\lambda}_1(N_{11}+\hat{\mu}_{y1})^2 + \hat{\lambda}_2(N_{12}+\hat{\mu}_{y2})^2}{2}\right), \tag{102}$$

where N_{11} and N_{12} are iid $\mathcal{N}(0,1)$.

Substituting (82) and (102) into (81) and rearranging yields

$$p = P\left(\frac{1-\omega_1^2}{2}\sum_{i=1}^{|S|}\left(N_i + \frac{\omega_1^2}{1-\omega_1^2}\beta_i\right)^2 + I_{11}\left[\frac{\hat{\lambda}_1}{2}(N_{11}+\hat{\mu}_{y1})^2 + \frac{\hat{\lambda}_2}{2}(N_{12}+\hat{\mu}_{y2})^2\right] > \hat{t}\right), \tag{103}$$

where

$$\hat{t} \triangleq \ln t - |S|\ln\omega_1 + \frac{\omega_1^2}{2(1-\omega_1^2)}\sum_{i=1}^{|S|}\beta_i^2 - I_{11}\Big(\omega_2 + \frac{K_0}{2}\Big). \tag{104}$$

The quantity on the left side of the inequality in (103) is a positive linear combination of independent non-central chi-squared random variables. We analyze this quantity using a fast and accurate approximation in [15], which is a generalization of a result for a non-central chi-squared random variable in [16]. The simpler result in [16] can be used directly if $I_{11} = 0$; here we apply the result in [15] to the general case.

Let $Q_k = \sum_{j=1}^k c_j(x_j + a_j)^2$ where i.i.d. $x_j \sim \mathcal{N}(0,1)$, $1 \le j \le k$, and $c_j > 0$. Further, let $\theta_s \triangleq \sum_{j=1}^k c_j^s(1 + s a_j^2)$, $s \ge 0$ and $h \triangleq 1 - 2\theta_1\theta_3/3\theta_2^2$. By [15], we have $(Q_k/\theta_1)^h \overset{d}{\approx} \mathcal{N}\left(\mu_Q, \sigma_Q^2\right)$, where $\mu_Q \triangleq 1 + \frac{\theta_2 h(h-1)}{\theta_1^2}$ and $\sigma_Q^2 \triangleq \frac{2\theta_2 h^2}{\theta_1^2}$. We did not include the next higher order terms of the approximation in [16] because they did not improve the accuracy in the right tail in our numerical calculations. Applying

this approximation to (103) yields

$$P\left\{Q_k > \hat{t}\right\} = p, \tag{105}$$

$$\Rightarrow\; P\left\{(Q_k/\theta_1)^h > (\hat{t}/\theta_1)^h\right\} \approx \bar{\Phi}\left(\frac{(\hat{t}/\theta_1)^h - \mu_Q}{\sigma_Q}\right) = p, \tag{106}$$

$$\Rightarrow\; \hat{t} \approx \theta_1\left[\mu_Q + \sigma_Q\bar{\Phi}^{-1}(p)\right]^{1/h}. \tag{107}$$

Calculating the FRR. Our analysis of FRR follows the same sequence of steps as our analysis of FAR. Equation (78) implies that the FRR, which is $P(L < t|H_1)$, can be expressed as

$$\begin{aligned} FRR =& P\left(|S|\ln\omega_1 + \sum_{i=1}^{|S|}\left[\frac{(\tilde{Z}_i - \mu_G)^2}{2\sigma_G^2} - \frac{(\tilde{Z}_i - \mu_i - \delta)^2}{2(\hat{\sigma}^2 + s^2)}\right]\right. \\ &\left. + I_{11}\left(\omega_2 - \frac{(\tilde{Z}_I^c)^T\Sigma_I^{-1}\tilde{Z}_I^c}{2} + \frac{(\tilde{Z}_I^c + \bar{\mu})^T\Sigma_{GI}^{-1}(\tilde{Z}_I^c + \bar{\mu})}{2}\right) < \ln t \mid H_1\right). \end{aligned} \tag{108}$$

Recall that $\tilde{Z}_i \sim \mathcal{N}(\mu_i + \delta, \hat{\sigma}^2 + s^2)$ are conditionally independent for $i = 1, \ldots, |S|$ under hypothesis H_1. If we define $M_i \triangleq (\tilde{Z}_i - \mu_i - \delta)/\sqrt{\hat{\sigma}^2 + s^2}$, then $M_i \sim \mathcal{N}(0,1)$. Simplifying the ith term inside the summation in (108) yields

$$\frac{(\tilde{Z}_i - \mu_G)^2}{2\sigma_G^2} - \frac{(\tilde{Z}_i - \mu_i - \delta)^2}{2(\hat{\sigma}^2 + s^2)} = \frac{1-\omega_1^2}{2\omega_1^2}\left(M_i + \frac{\omega_1\beta_i}{1-\omega_1^2}\right)^2 - \frac{\omega_1^2\beta_i^2}{2(1-\omega_1^2)}. \tag{109}$$

Recall that under hypothesis H_1, $\tilde{Z}_I^c \sim \mathcal{N}(0, \Sigma_I)$. Repeating the steps used to derive the FAR, we use (85) to express the irises part of (108) as

$$I_{11}\left(\omega_2 + \frac{(\tilde{Z}_I^c + \gamma_1)^T Q_1(\tilde{Z}_I^c + \gamma_1) + K_1}{2}\right), \tag{110}$$

which by (84) equals

$$I_{11}\left(\omega_2 + \frac{K_1}{2} + \frac{\sum_{i=1}^2 \bar{\lambda}_i\bar{y}_i^2}{2}\right), \tag{111}$$

where from the discussion below (84), we have that $\bar{y} = (\bar{y}_1 \;\; \bar{y}_2)^T \sim \mathcal{N}(\bar{\mu}_y, I)$, $\bar{A}$ is obtained using Cholesky's decomposition of $\Sigma_I = \bar{A}\bar{A}^T$, and $\bar{\lambda} = (\bar{\lambda}_1 \;\; \bar{\lambda}_2)^T$ and $\bar{P}$ are the eigenvalues and eigenvectors

of $\bar{A}^T Q_1 \bar{A}$. Explicitly evaluating these, we have

$$\bar{A} = \sigma_I \begin{pmatrix} 1 & 0 \\ \rho_I & \sqrt{1-\rho_I^2} \end{pmatrix}, \quad \bar{P} = \frac{1}{\sqrt{2}} \begin{pmatrix} \sqrt{1+\rho_I} & \sqrt{1-\rho_I} \\ \sqrt{1-\rho_I} & -\sqrt{1+\rho_I} \end{pmatrix}, \tag{112}$$

and the final parameters of interest

$$\bar{\lambda}_1 = \frac{\sigma_I^2(1+\rho_I)}{\sigma_{GI}^2} - 1, \tag{113}$$

$$\bar{\lambda}_2 = \frac{\sigma_I^2(1-\rho_I)}{\sigma_{GI}^2} - 1, \tag{114}$$

$$\bar{\mu}_y = \begin{pmatrix} \bar{\mu}_{y1} \\ \bar{\mu}_{y2} \end{pmatrix} \triangleq \frac{1}{\sqrt{2(1-\rho_I^2)}\sigma_I} \begin{pmatrix} \sqrt{1-\rho_I} & \sqrt{1-\rho_I} \\ \sqrt{1+\rho_I} & -\sqrt{1+\rho_I} \end{pmatrix} [I - \Sigma_{GI}\Sigma_I^{-1}]^{-1}\bar{\mu}. \tag{115}$$

By (115), we can re-express (111) as

$$I_{11}\left(\omega_2 + \frac{K_1}{2} + \frac{\bar{\lambda}_1(M_{11}+\bar{\mu}_{y1})^2 + \bar{\lambda}_2(M_{12}+\bar{\mu}_{y2})^2}{2}\right), \tag{116}$$

where M_{11} and M_{12} are iid $\mathcal{N}(0,1)$.

Substituting (109) and (116) into (108) and rearranging yields

$$\text{FRR} = P\left\{ \frac{1-\omega_1^2}{2\omega_1^2} \sum_{i=1}^{|S|} \left(M_i + \frac{\omega_1}{1-\omega_1^2}\beta_i \right)^2 + I_{11}\left[\frac{\bar{\lambda}_1}{2}(M_{11}+\bar{\mu}_{y1})^2 + \frac{\bar{\lambda}_2}{2}(M_{12}+\bar{\mu}_{y2})^2 \right] < \bar{t} \right\}, \tag{117}$$

where

$$\bar{t} \triangleq \ln t - |S|\ln\omega_1 + \frac{\omega_1^2}{2(1-\omega_1^2)} \sum_{i=1}^{|S|} \beta_i^2 - I_{11}\Big(\omega_2 + \frac{K_1}{2}\Big). \tag{118}$$

As in (105)-(106), we use the approximation in [15], but we now include the higher order terms in [16] to improve the accuracy in the left tail. As before, noting that the random variable inside the probability in (117) is of the form $\tilde{Q}_k = \sum_{j=1}^k c_j(x_j+a_j)^2$ where $x_j \sim \mathcal{N}(0,1)$ iid for $1 \le j \le k$ with $c_j > 0$, and defining $\tilde{\theta}_s$ and $\tilde{h}$ analogously, we have $(\tilde{Q}_k/\tilde{\theta}_1)^{\tilde{h}} \overset{d}{\approx} \mathcal{N}\left(\tilde{\mu}_Q, \tilde{\sigma}_Q^2\right)$, where $\tilde{\mu}_Q \triangleq 1 + \frac{\tilde{\theta}_2\tilde{h}(\tilde{h}-1)}{\tilde{\theta}_1^2} - \frac{\tilde{\theta}_2^2\tilde{h}(\tilde{h}-1)(2-\tilde{h})(1-3\tilde{h})}{2\tilde{\theta}_1^4}$ and $\tilde{\sigma}_Q^2 \triangleq \frac{2\tilde{\theta}_2\tilde{h}^2}{\tilde{\theta}_1^2}\left(1 - \frac{\tilde{\theta}_2(1-\tilde{h})(1-3\tilde{h})}{2\tilde{\theta}_1^2}\right)^2$. Applying this approximation directly to compute the probability (117), however, proves inaccurate for our application. The quality of this approximation deteriorates with increasing variation among the c_j's [15], and the c_j's corresponding to the iris terms, $\frac{\bar{\lambda}_1}{2}$ and $\frac{\bar{\lambda}_2}{2}$,

which are both of the same order, are significantly larger than $\frac{1-\omega_1^2}{2\omega_1^2}$, which is the c_j for the finger terms. To circumvent this problem, we split $\tilde{Q}_k$ as $\tilde{Q}_k^F + \tilde{Q}_k^I$, where $\tilde{Q}_k^F$ ($\tilde{Q}_k^I$) consists of the finger (iris) terms exclusively and is thus approximated accurately using the scheme described above. To compute FRR=P($\tilde{Q}_k^F + \tilde{Q}_k^I < \bar{t}$) using the approximated independent marginals of $\tilde{Q}_k^F$ and $\tilde{Q}_k^I$ is analytically intractable, so we instead use the trapezoidal approximation with $\tilde{N}$ terms:

$$\begin{aligned} P(\tilde{Q}_k^F + \tilde{Q}_k^I < \bar{t}) &\approx \sum_{i=1}^{\tilde{N}} P\left(\tilde{Q}_k^F \in \left[(i-1)\bar{t}/\tilde{N}, i\bar{t}/\tilde{N}\right],\ \tilde{Q}_k^I < (1-i/\tilde{N})\bar{t}\right) \\ &= \sum_{i=1}^{\tilde{N}} \left[P\left(\tilde{Q}_k^F < i\bar{t}/\tilde{N}\right) - P\left(\tilde{Q}_k^F < (i-1)\bar{t}/\tilde{N}\right)\right] P\left(\tilde{Q}_k^I < (1-i/\tilde{N})\bar{t}\right), \end{aligned} \tag{119}$$

where the equality follows from the independence of $\tilde{Q}_k^F$ and $\tilde{Q}_k^I$. Each term in (119) may be computed based on our approximation, as was done in (105)-(106). We chose a value of $\tilde{N} = 20$ in our numerical computations.

Ranking the Fingers. Until now, our analysis has ignored the decision of which $|S|$ fingers to acquire. Rather than evaluating all $\binom{10}{|S|}$ possibilities, it turns out that there is an optimal ranking of the fingers, regardless of the value of $|S|$. To derive this ranking, first fix the number of fingers $|S|$ that may be scanned. Next, note that the distribution of the first random variable in (117) is non-central chi squared with $|S|$ degrees of freedom and non-centrality parameter $\left(\frac{\omega_1^2}{1-\omega_1^2}\right)^2 \sum_{i=1}^{|S|} \beta_i^2$; thus, the value of $\hat{t}$ satisfying (104) depends on $(\beta_1, \ldots, \beta_{|S|})$ only via $\sum_{i=1}^{|S|} \beta_i^2$. Similarly, the first non-central chi-squared random variable in (117) has $|S|$ degrees of freedom and noncentrality parameter $\lambda' \triangleq \left(\frac{\omega_1}{1-\omega_1^2}\right)^2 \sum_{i=1}^{|S|} \beta_i^2$; combining with the conclusion for $\hat{t}$, this implies that the FRR depends on $(\beta_1, \ldots, \beta_{|S|})$ only via $\sum_{i=1}^{|S|} \beta_i^2$.

Next, we note that $\sqrt{\sum_{i=1}^{|S|} \beta_i^2}$ is the scaled Euclidean distance between the means of the log similarity scores of fingers under the two hypotheses H_0 and H_1. Therefore, holding all else fixed, a higher $\sum_{i=1}^{|S|} \beta_i^2$ implies a greater difference – and hence a greater ability to distinguish – between the two hypotheses; this should reflect in a lower FRR at the same level of FAR. It is easy to prove that this argument is always true when the standard deviations under the hypotheses H_0 and H_1 are identical, and we are close to this case as the ratio of these standard deviations is 0.87 for the exclusion scenario and 0.75 for the inclusion scenario. Further, even when this ratio is away from 1, the conclusion still holds at levels of FAR that are small enough, such as those in our case.

It follows that if we are to acquire exactly $|S|$ fingers to minimize FRR, it is nearly optimal to choose

those with the $|S|$ highest values of β_i^2, or equivalently $|\beta_i|$. This argument does not depend on the value of $|S|$, and hence the ranking based on $|\beta_i|$ does not depend on $|S|$.

Because the Neyman-Pearson lemma considers a deviation of the log similarity score away from μ_G – whether positive or negative – as a departure from the null hypothesis, it allows for $\beta_i < 0$. However, it will typically be the case that $\beta_i > 0$ because the expected value of the log similarity score should be higher when the match is genuine than when the match is between two different residents, which implies that $\mu_i + \delta > \mu_G$. Hence, for practical purposes, we choose to rank the fingers by β_i rather than $|\beta_i|$, which by (83) is equivalent to ranking by μ_i; by (65), this ranking reduces to $\frac{\mu_\theta \sigma_3^2}{\sigma^2} c_i + \tilde{Y}_i$, which depends on both the population-wide average c_i of finger i and the observed similarity score $\tilde{Y}_i$ of finger i.

Solving (61)-(62). For each resident, we define the subscripts $[i] = 1, \ldots, 10$ such that $\mu_{[1]} \geq \mu_{[2]} \geq \cdots \geq \mu_{[10]}$. This ranking of the fingers reduces the optimization problem in (61)-(62) to

$$\min_{|S|, I_{11}} \quad \text{FRR} + \tilde{\lambda}(c_F|S| + c_I I_{11}), \tag{120}$$

where FRR is given in (117)-(119). This optimization problem can be easily solved (it has only 21 feasible solutions).

3.2 Two-stage Policies

In general, a two-stage policy takes as input a resident's observed log similarity scores during BFD and BID, $\tilde{Y} = (\tilde{Y}_1, \ldots, \tilde{Y}_{12})$, and chooses to acquire in the first stage a subset S_1 of the 10 fingers, along with either neither or both irises (due to the use of a dual-eye camera). We then observe the new similarity scores $\tilde{Z}^{(1)}$ for the acquired subset and either accept the resident, reject the resident or continue to the second stage to acquire more biometrics. If the second stage is reached, it chooses to acquire a subset of the 10 fingers and both irises that have not already been used in the first stage. We then observe the new similarity scores $\tilde{Z}^{(2)}$ for the acquired subset of second stage and make an accept/reject decision.

Because this problem is difficult to solve, we only consider three restrictive classes of policies. In the first class, we consider the iris-finger policy, in which we acquire both irises and no fingers in the first stage, and no irises and a subset of the 10 fingers in the second stage. Next we consider the finger-iris policy, in which, analogous to the iris-finger policy, we may acquire only fingers in the first stage and only both irises in the second stage. Finally, the least restrictive class we consider is the either-other policy,

that permits the use of any one mode of biometrics – either fingers or irises – in the first stage and the use of only the other mode in the second stage.

We also make several simplifying assumptions. As before for the single-stage policies, we impose the same FAR constraint for each resident and we account for the constraint on average delay using an additive delay penalty with Lagrange multiplier $\tilde{\lambda}$. In addition, if the policy proceeds to the second stage, we impose that the second-stage FAR be independent of the observed similarity scores $\tilde{Z}^{(1)}$ from the first stage. Note that the second-stage FAR may still be different across residents. This assumption, while suboptimal, considerably simplifies the analysis of the policy and makes it possible to express the optimal policy in a compact form.

As before, our overall goal is to minimize the sum of FRR and the delay penalty subject to a constraint on the FAR. We note that in contrast to the single-stage policies, the delay in the two-stage case is variable, depending on whether or not the first-stage similarity scores are inconclusive in making an accept/reject decision. Therefore, we now use $\tilde{\lambda}$ times the expected delay as the delay penalty in our objective function, where the expectation is taken under the hypothesis that the resident is genuine.

Overview. In our analysis of two-stage policies, we re-use much of the notation from §3.1 but add a subscript or superscript to denote the stage. Let the biometric acquisition decisions be given by S_i and $I_{11}^{(i)}$ for stage $i = 1, 2$, let $\tilde{Z}^{(i)}$ be the set of similarity scores for the biometrics acquired in stage i, and let L_i be the likelihood ratio based on the similarity scores acquired only in stage i. We change notation for the thresholds (Fig. 1 in the main text) and let t_U and t_L be the upper and lower thresholds in stage 1 and let t_2 be the threshold for stage 2. Thus, if $L_1 < t_L$ we reject the person as imposter, if $L_1 > t_U$ we accept the person as genuine, and we otherwise go to stage 2. In stage 2, we compare L_2 with t_2 to make the accept/reject decision (we prove in our analysis that it is optimal under our assumptions to discard L_1 when making the stage 2 decision). Finally, we define $D_i(S_i, I_{11}^{(i)})$ to be the delay due to the biometrics acquired in stage i, as given in Table 2 of the main text.

As mentioned earlier, we analyze three classes of two-stage policies which differ in the restrictions that they place on the set of feasible biometrics $(S_1, I_{11}^{(1)}, S_2, I_{11}^{(2)})$ that may be acquired. We define these

feasible sets as

$$\mathcal{C}_{iris-finger} = \left\{ \left(S_1, I_{11}^{(1)}, S_2, I_{11}^{(2)} \right) : S_2 \subseteq \{1,\ldots,10\}, I_{11}^{(1)} \in \{0,1\}, |S_1| = 0, I_{11}^{(2)} = 0 \right\},$$
$$\mathcal{C}_{finger-iris} = \left\{ \left(S_1, I_{11}^{(1)}, S_2, I_{11}^{(2)} \right) : S_1 \subseteq \{1,\ldots,10\}, I_{11}^{(2)} \in \{0,1\}, |S_2| = 0, I_{11}^{(1)} = 0 \right\},$$
$$\mathcal{C}_{either-other} = \mathcal{C}_{iris-finger} \cup \mathcal{C}_{finger-iris}. \tag{121}$$

Note that for each of these policies, by construction, the mode of biometrics acquired in stage 2 differs from that in stage 1. By our assumption of independence between iris and finger scores, this implies that $\tilde{Z}^{(1)}$ and $\tilde{Z}^{(2)}$ (and hence, L_1 and L_2) are independent – a fact that will be harnessed in our analysis.

All classes of two-stage policies are now analyzed under a unified framework in which we fix the policy class and refer to the corresponding feasible set in (121) by $\mathcal{C}$. Our strategy to find the optimal policy is divided in two steps: (i) fix the set of biometrics $(S_1, I_{11}^{(1)}, S_2, I_{11}^{(2)})$ acquired in each stage and find the optimal policy parameters (t_L, t_U, t_2) as well as the optimal objective value, and (ii) determine the optimal set of biometrics to acquire by making comparisons across their objective values. By ranking the fingers by β_i as in the single-stage policies, we need to consider only 10, 10 and 20 different feasible sets for the iris-finger, finger-iris and either-other policy, respectively.

We also provide implementation details that facilitated effective computation of these policies.

Optimal Policy for a Fixed Set of Biometrics. In the first step, we fix the set of biometrics $(S_1, I_{11}^{(1)}, S_2, I_{11}^{(2)})$ to be acquired in stage 1 and 2, and note an elementary but important fact: the second-stage decision t_2 will, in general, depend on the realized similarity scores $\tilde{Z}^{(1)}$ from stage 1 as well as on the choice of t_L and t_U. Letting $F = (t_L, t_U, \tilde{Z}^{(1)})$ denote the information at the beginning of stage 2 and $\mathcal{F}$ be the set of all possible values of F, we have that t_2 is a real function on $\mathcal{F}$; i.e., $t_2(F) \in \mathbb{R}, \forall F \in \mathcal{F}$. However, we shall prove shortly that under our restriction that the FAR in stage 2 be constant for all values of $\tilde{Z}^{(1)}$, it follows that $t_2(F)$ is constant for all F with fixed t_L, t_U; this allows for any policy to be expressed in a compact form as a triplet $(t_L, t_U, t_2) \in \mathbb{R}^3$.

We now write the expressions for FAR, FRR and the expected delay $(\bar{D})$ in terms of (t_L, t_U, t_2). Defining $\mathcal{S}(\mathcal{F})$ to be the space of all real functions on $\mathcal{F}$, we place no restrictions on $t_2 \in \mathcal{S}(\mathcal{F})$. Let t_2' be any real-valued function on $\mathcal{F}$ and define the function t_2 such that $t_2(F) = t_2'(F)/L_1, \forall F \in \mathcal{F}$ (note that t_2 is well-defined since L_1 is a function of $\tilde{Z}_1$, which is an element of F). We know from the Neyman-Pearson lemma that it is optimal to make the decisions in stage 1 based on L_1 and those in

stage 2 based on L_1L_2. Thus, we have

$$\bar{D} = D_1(S_1, I_{11}^{(1)}) + P(L_1 \in [t_L, t_U]|H_1)D_2(S_2, I_{11}^{(2)}), \tag{122}$$

$$\begin{aligned}
\text{FRR} &= P(L_1 < t_L|H_1) + P(L_1 \in [t_L, t_U], L_1L_2 < t_2'(F)|H_1), \\
&= P(L_1 < t_L|H_1) + P(L_1 \in [t_L, t_U]|H_1)P(L_1L_2 < t_2'(F)|L_1 \in [t_L, t_U], H_1), \\
&= P(L_1 < t_L|H_1) + P(L_1 \in [t_L, t_U]|H_1)P(L_2 < t_2(F)|L_1 \in [t_L, t_U], H_1), \\
&= P(L_1 < t_L|H_1) + P(L_1 \in [t_L, t_U]|H_1)E\Big[P(L_2 < t_2(F)|F, H_1)\Big|L_1 \in [t_L, t_U], H_1\Big], \qquad (123)\\
\text{FAR} &= P(L_1 > t_U|H_0) + P(L_1 \in [t_L, t_U], L_1L_2 > t_2'(F)|H_0), \\
&= P(L_1 > t_U|H_0) + P(L_1 \in [t_L, t_U]|H_0)P(L_1L_2 > t_2'(F)|L_1 \in [t_L, t_U], H_0), \\
&= P(L_1 > t_U|H_0) + P(L_1 \in [t_L, t_U]|H_0)P(L_2 > t_2(F)|L_1 \in [t_L, t_U], H_0), \\
&= P(L_1 > t_U|H_0) + P(L_1 \in [t_L, t_U]|H_0)E\Big[P(L_2 > t_2(F)|F, H_0)\Big|L_1 \in [t_L, t_U], H_0\Big]. \qquad (124)
\end{aligned}$$

Our goal is to solve

$$\begin{aligned}
&\min_{\substack{t_L, t_U \in \mathbb{R} \\ t_2 \in \mathcal{S}(\mathcal{F})}} \quad \text{FRR} + \tilde{\lambda}\bar{D} \\
&s.t. \quad \text{FAR} = p. \qquad (125)
\end{aligned}$$

Substituting (122)-(124) into (125) yields

$$\begin{aligned}
\min_{\substack{t_L, t_U \in \mathbb{R} \\ t_2 \in \mathcal{S}(\mathcal{F})}} \quad & P(L_1 < t_L|H_1) + \tilde{\lambda}D_1(S_1, I_{11}^{(1)}) \\
&+ P(L_1 \in [t_L, t_U]|H_1)E\Big[P(L_2 < t_2(F)|F, H_1) + \tilde{\lambda}D_2(S_2, I_{11}^{(2)})\Big|L_1 \in [t_L, t_U], H_1\Big] \\
\text{s.t.} \quad & P(L_1 > t_U|H_0) + P(L_1 \in [t_L, t_U]|H_0)E\Big[P(L_2 > t_2(F)|F, H_0)\Big|L_1 \in [t_L, t_U], H_0\Big] = p. \qquad (126)
\end{aligned}$$

Let us define the second-stage FAR by

$$p' \triangleq \frac{p - P(L_1 > t_U|H_0)}{P(L_1 \in [t_L, t_U]|H_0)}, \tag{127}$$

which is only a function of t_L and t_U. Next, in (126), we move the minimization over t_2 inside to

equivalently obtain

$$\min_{t_L, t_U \in \mathbb{R}} \; P(L_1 < t_L|H_1) + \tilde{\lambda} D_1(S_1, I_{11}^{(1)}) \tag{128}$$
$$+ P(L_1 \in [t_L, t_U]|H_1) E\Big[\min_{\substack{t_2(F)\in\mathbb{R} \\ \text{s.t. } E[P(L_2 > t_2(F)|F,H_0)|L_1 \in [t_L,t_U],H_0] = p'}} P(L_2 < t_2(F)|F, H_1) + \tilde{\lambda} D_2(S_2, I_{11}^{(2)}) \Big| L_1 \in [t_L, t_U], H_1\Big].$$

Note that in the inner optimization problem, while the value of t_2 may be optimized path by path for each realized value of F, the constraint only applies in an average sense (i.e., on the expected value over all realizations of $F \in \mathcal{F}$). This makes it hard to solve for the optimal $t_2 \in \mathcal{S}(\mathcal{F})$, and even if one did, it would still be hard to evaluate the expectation in (128) in order to solve for the optimal t_L and t_U in the outer optimization. For this reason, we impose that the constraint in (128) holds path by path, i.e., $P(L_2 > t_2(F)|F, H_0) = p' \; \forall F \in \mathcal{F}$. This is our aforementioned assumption to let the FAR in stage 2 be the same for all realizations of the stage 1 similarity scores $\tilde{Z}^{(1)}$. Further, the optimal threshold $t_2(F)$ now only depends on p' which is only a function of t_L and t_U; as a consequence, the optimal t_2 does not depend on the stage 1 similarity score $\tilde{Z}^{(1)}$ and the optimal policy is characterized by the triplet $(t_L, t_U, t_2) \in \mathbb{R}^3$. Henceforth, we simply assume that $t_2 \in \mathbb{R}$.

We next exploit the independence between L_2 (which is a function of $\tilde{Z}^{(2)}$) and $\tilde{Z}^{(1)}$ as discussed below (121). Using the fact that t_2 is now just a policy constant, we can greatly simplify the expressions for FRR and FAR in (123)-(124) to get

$$\text{FRR} = P(L_1 < t_L|H_1) + P(L_1 \in [t_L, t_U]|H_1) P(L_2 < t_2|H_1), \tag{129}$$

$$\text{FAR} = P(L_1 > t_U|H_0) + P(L_1 \in [t_L, t_U]|H_0) P(L_2 > t_2|H_0). \tag{130}$$

Using (127) and (130), we express the constraint $FAR = p$ as $P(L_2 > t_2|H_0) = p'$. Substituting this and (129) in (125) yields the optimization problem that we finally solve:

$$\min_{t_L, t_U, t_2 \in \mathbb{R}} \; P(L_1 < t_L|H_1) + \tilde{\lambda} D_1(S_1, I_{11}^{(1)}) + P(L_1 \in [t_L, t_U]|H_1)\Big[P(L_2 < t_2|H_1) + \tilde{\lambda} D_2(S_2, I_{11}^{(2)})\Big]$$
$$\text{s.t.} \;\; P(L_2 > t_2|H_0) = p'. \tag{131}$$

To solve (131), we perform a grid search of (t_L, t_U). At any estimate of this pair, the value of p' is implicitly defined, and t_2 may then be obtained using the constraint.

Before closing this discussion, we describe how to compute the probabilities in the objective function of (131), as well as the procedure to invert the probability in the constraint to obtain t_2. Recall that the log likelihood, $\ln L_i$, is just the expression (78) with S, I_{11} and $\tilde{Z}$ substituted by S_i, $I_{11}^{(i)}$ and $\tilde{Z}^{(i)}$, for $i = 1, 2$. This enables us to use the machinery already developed in §3.1 to compute and invert all the probabilities involved. Thus, to invert the constraint in (131) for a given p', we note that it is identical to (81) with $S, I_{11}, \tilde{Z}, t$ and p substituted by $S_2, I_{11}^{(2)}, \tilde{Z}^{(2)}, t_2$ and p', and we follow the same procedure to obtain t_2. Next, by writing $P(L_1 \in [t_L, t_U]|H_1)$ as $P(L_1 < t_U|H_1) - P(L_1 < t_L|H_1)$, we express all probabilities in the objective function of (131) as $P(L_i < t|H_1)$ for some $t \in \mathbb{R}$ and $i = 1, 2$. Any probability of the form $P(L_i < t|H_1)$ is identical to the right hand side of (108) with S, I_{11} and $\tilde{Z}$ substituted by $S_i, I_{11}^{(i)}$ and $\tilde{Z}^{(i)}$, and it can be computed using the procedure to compute FRR laid out there.

Optimal Set of Biometrics. Let $(S_1, I_{11}^{(1)}, S_2, I_{11}^{(2)}) \in \mathcal{C}$ be the set of biometrics we choose to acquire for a fixed two-stage policy. As we know how to compute the optimal parameters (t_L, t_U, t_2) for a fixed set of biometrics, computing the optimal objective value in (131) is straightforward. By choosing the set $(S_1, I_{11}^{(1)}, S_2, I_{11}^{(2)}) \in \mathcal{C}$ with the minimum optimal objective value, we obtain the optimal set of biometrics to acquire. However, for each two-stage policy there are more than 1,000 feasible sets in $\mathcal{C}$, which makes it prohibitively expensive to compute the optimal parameters for each set. Fortunately, a major reduction is possible by following the same line of arguments for ranking fingers as in §3.1.

Turning to the iris-finger policy, in which the fingers may be used in stage 2 only and the similarity scores of fingers appear only in L_2, we consider all sets of fingers S_2, which have $|S_2|$ fingers. Next, we fix the values of t_L, t_U in (131) so that p' is now fixed. As noted earlier, $\ln L_2$ is just the expression (78) with S, I_{11} and $\tilde{Z}$ substituted by S_2, $I_{11}^{(2)}$ and $\tilde{Z}^{(2)}$. We already know from §3.1 that for a fixed FAR$=$ $p' = P(L_2 > t_2|H_0)$, increasing $\sum_{i=1}^{|S_2|} \beta_i^2$ decreases the FRR$=$ $P(L_2 < t_2|H_1)$, which in turn decreases the objective function in (131). Therefore, for this value of t_L and t_U, it is optimal to rank the fingers based on β_i as before and to choose the set S_2 consisting of the top $|S_2|$ fingers. Because the ranking based on β_i does not depend on the values of t_L and t_U, the set S_2 of the top $|S_2|$ fingers provides the lowest objective value in (131) among all sets with $|S_2|$ fingers. Because $1 \leq |S_2| \leq 10$, there are only 10 different sets S_2 that we need to evaluate to arrive at the optimal set of biometrics to acquire.

For the finger-iris policy, we make a more fundamental argument because a direct comparison with the single-stage case (as we did for the iris-finger policy) does not work due to the presence of the confounding

terms $P(L_1 \in [t_L, t_U]|H_i)$ in the FRR and FAR. Instead, we first note that the inference in the second stage (using only irises) is independent of the choice of fingers in the first stage because the similarity scores $\tilde{Z}^{(1)}$ are never used in stage 2. Hence, improving the inference in stage 1 by using the optimal set of fingers can only lower the overall FRR for a given FAR. Next, following the line of arguments for the single-stage case, we know that heuristically a higher $\sum_{i=1}^{|S|} \beta_i^2$ provides a greater difference, and hence a greater ability to discern, between the two hypotheses H_0 and H_1. Therefore, we rank the fingers based on β_i as before and choose the set S_1 consisting of the top $|S_1|$ fingers, for fixed $1 \leq |S_1| \leq 10$, to improve the inference in the first stage. As claimed earlier, among the sets with $|S_1|$ fingers, using S_1 lowers the overall FRR for the given FAR, and this fact allows us to make comparisons across just 10 sets to arrive at the optimal set of biometrics to acquire.

Finally, it follows from the definition of $\mathcal{C}_{either-other}$ in (121) that we only need to compare across 20 sets to obtain the optimal set of biometrics for the either-other policy: the best set S_2 for the iris-finger policy and the best set S_1 for the finger-iris policy containing n fingers, for each $1 \leq n \leq 10$.

Implementation Details. The computation of the optimal two-stage policies consumes significantly more time than the single-stage policies. In contrast to an average computation time of 0.002 seconds for determining the optimal parameters for the single-stage policies, the same step is ≈ 100 times slower for the two-stage policies due to the numerical optimization step over t_L and t_U. Hence, it is important to choose a good initial solution. We do this by first solving for the threshold t_1 under the assumption that it is never optimal to proceed to the second stage (i.e., $t_L = t_U$), which amounts to computing the single-stage optimal policy. We then use as initial solution $t_L = t_1 - \epsilon$ and $t_U = t_1 + \epsilon$ for a small value $\epsilon > 0$, which typically yields a smooth convergence. In addition, for those residents for whom the single-stage policy is already good enough (e.g., guarantees an FRR $< 10^{-9}$), we skip the optimization step altogether and simply treat the single-stage policy as optimal. This significantly reduces the computation time and results in no change in our simulated results.

References

1. Unique Identification Authority of India (2012) Role of biometric technology in Aadhaar Authentication. Technical report, UIDAI, New Delhi, India. Available: http://uidai.gov.in/images/role_of_biometric_technology_in_aadhaar_authentication_020412.pdf.

Accessed August 30, 2012.

2. International Organization for Standardization (2006) Information technology - biometric performance testing and reporting - part 1: principles and framework. ISO/IEC 19795-1. Technical report. Available: http://www.iso.org/iso/home/store/catalogue_tc/catalogue_detail.htm?csnumber=41447. Accessed September 4, 2012.

3. Stroud AH (1971) Approximate calculation of multiple integrals. Prentice-Hall, Englewood Cliffs, NJ.

4. Krylov VI, Stroud AH (2006) Approximate calculation of integrals. Dover Publications, Mineola, NY.

5. Eaton ML (1983) Multivariate statistics: a vector space approach. John Wiley & Sons, New York.

6. Karatzas I, Shreve SE (1988) Brownian motion and stochastic calculus. Spring-Verlag, New York.

7. Clark CE (1961) The greatest of a finite set of random variables. Operations Research 9: 145–162.

8. Asmussen S, Rojas-Nandayapa L (2008) Asymptotics of sums of lognormal random variables with Gaussian copula. Statistics & Probability Letters 78: 2709–2714.

9. UIDAI (2012) Role of biometric technology in Aadhaar Authentication: Iris authentication accuracy - PoC Report. Technical report, UIDAI, New Delhi, India. Available: http://uidai.gov.in/images/role_of_biometric_technology_in_aadhaar_authentication_020412.pdf. Accessed August 30, 2012.

10. Daugman J (2013) Results from 200 billion iris cross-comparisons, UCAM-CL-TR-635. Technical report, University of Cambridge, Cambridge, UK. Available: www.cl.cam.ac.uk/techreports/UCAM-CL-TR-635.pdf. Accessed July 17, 2013.

11. Phillips PJ, Bowyer KW, Flynn PJ, Liu X, Scruggs WT (2008) The Iris Challenge Evaluation 2005. In: 2nd IEEE International Conference on Biometrics: Theory, Applications and Systems (BTAS 08). IEEE, pp. 1–8.

12. Fenton L (1960) The sum of log-normal probability distributions in scatter transmission systems. IRE Transactions on Communications Systems 8: 57–67.

13. Prabhakar S, Jain AK (2002) Decision-level fusion in fingerprint verification. Pattern Recognition 35: 861–874.

14. Provost SB, Rudiuk EM (1996) The exact distribution of indefinite quadratic forms in noncentral normal vectors. Ann Inst Statist Math 48: 381–394.

15. Jensen DR, Solomon HA (1972) Gaussian approximation to the distribution of a definite quadratic form. JASA 67: 898–902.

16. Sankaran, M (1959) On the non-central χ^2 distribution. Biometrika 46: 235–237.

Figures

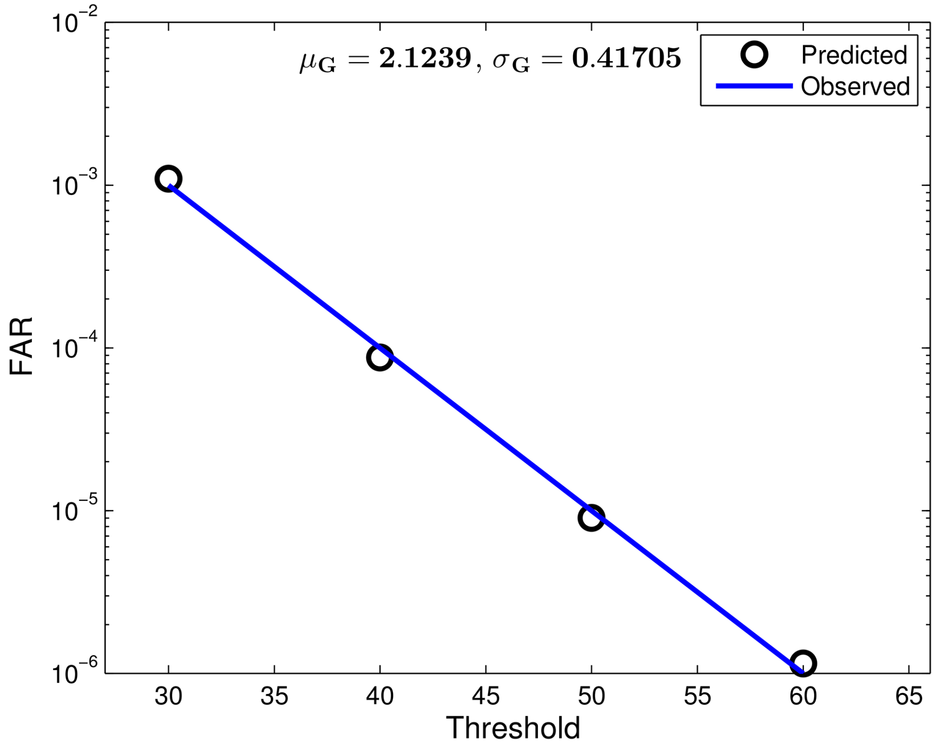

Figure 1. The fit of the lognormal imposter distribution to the observed (FAR,threshold) pairs.

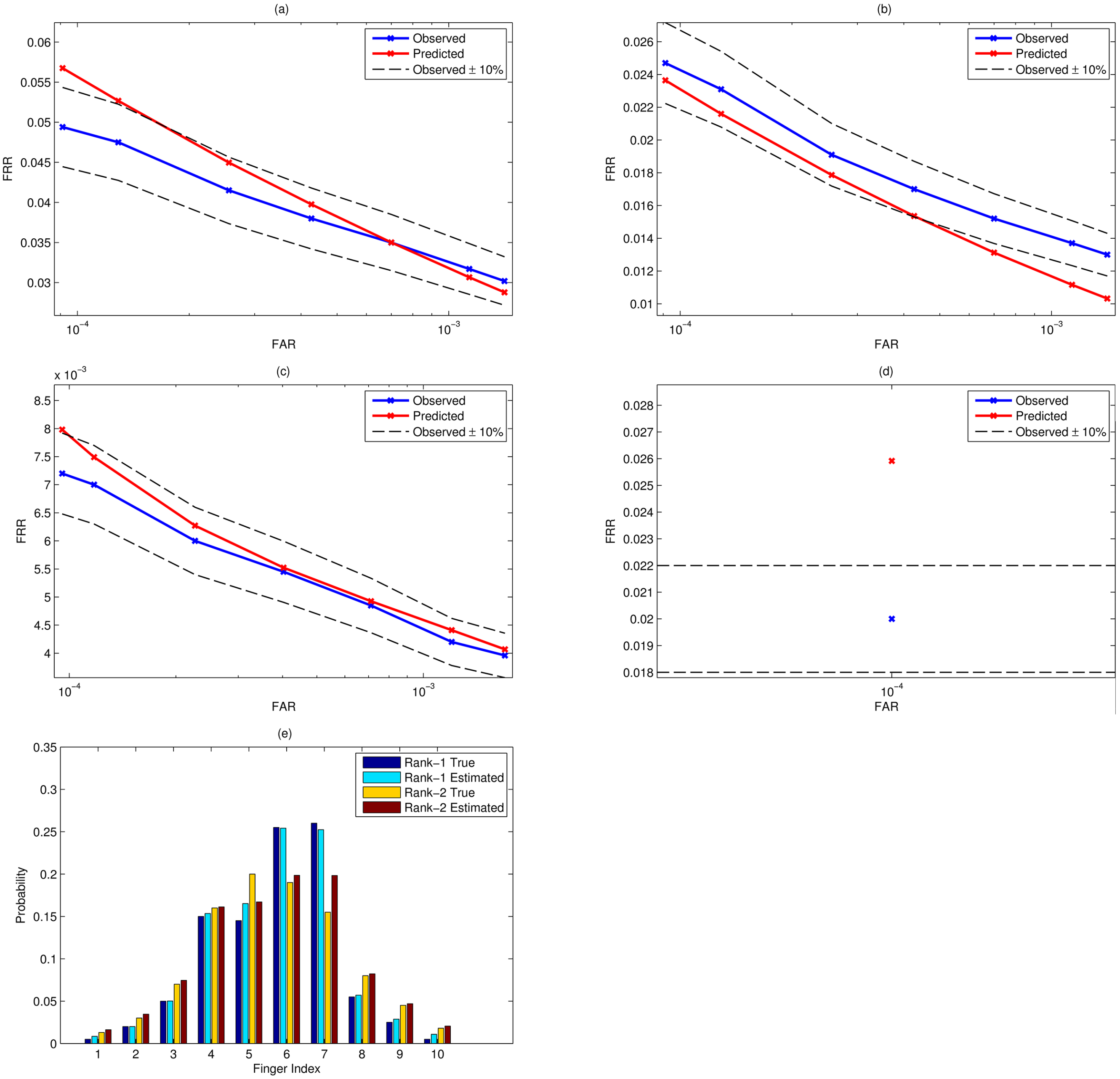

Figure 2. The predicted vs. observed results during the fingerprint parameter estimation procedure for the exclusion scenario. FRR vs. FAR curves for **(a)** the single-finger single-attempt case, **(b)** the single-finger multiple-attempt case, **(c)** the two-finger multi-attempt case, and **(d)** the two-finger single-attempt case, and **(e)** the rank-1 and rank-2 probabilities for the 10 fingers. package.

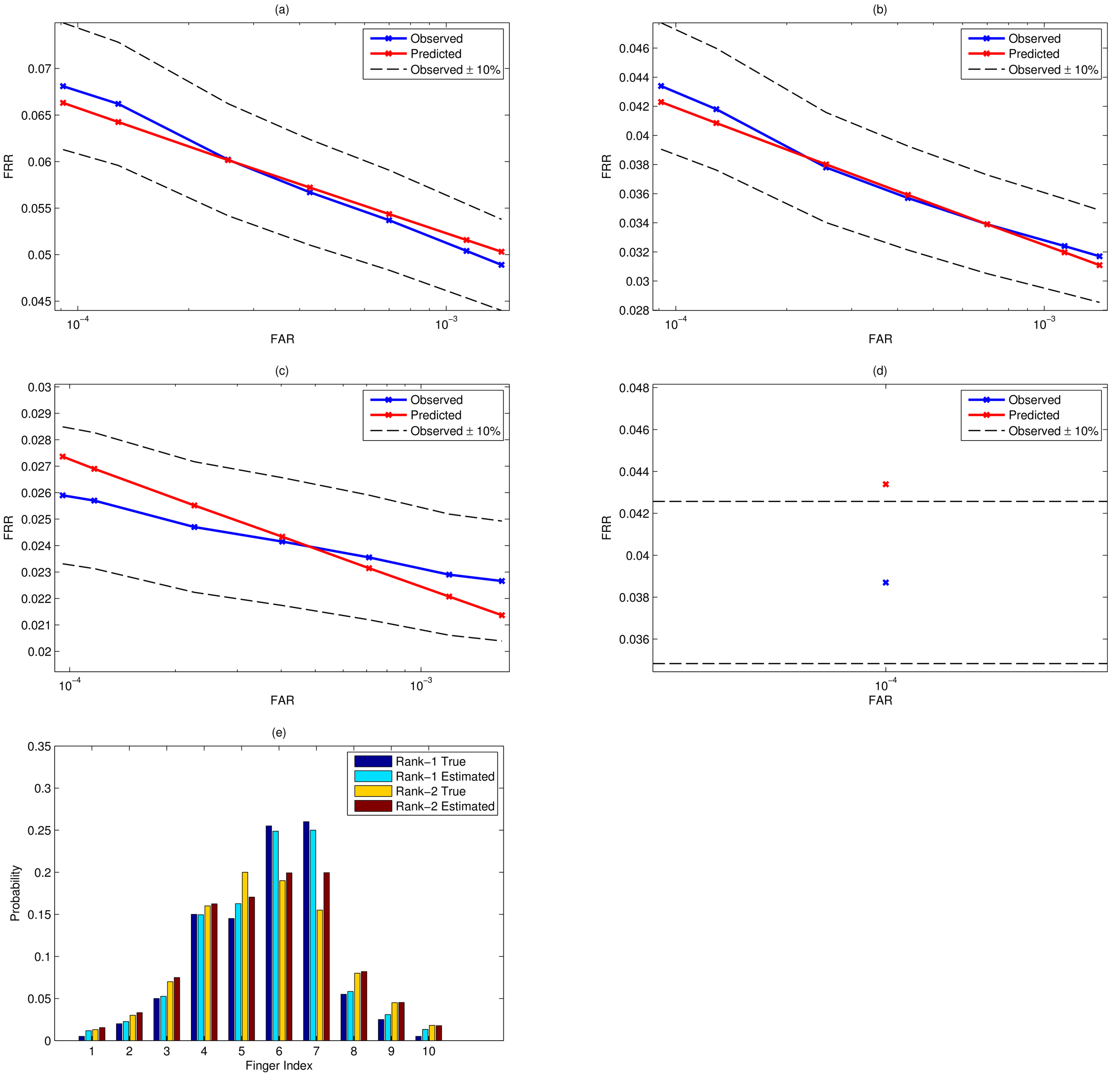

Figure 3. The predicted vs. observed results during the fingerprint parameter estimation procedure for the inclusion scenario. FRR vs. FAR curves for **(a)** the single-finger single-attempt case, **(b)** the single-finger multiple-attempt case, **(c)** the two-finger multi-attempt case, and **(d)** the two-finger single-attempt case, and **(e)** the rank-1 and rank-2 probabilities for the 10 fingers.

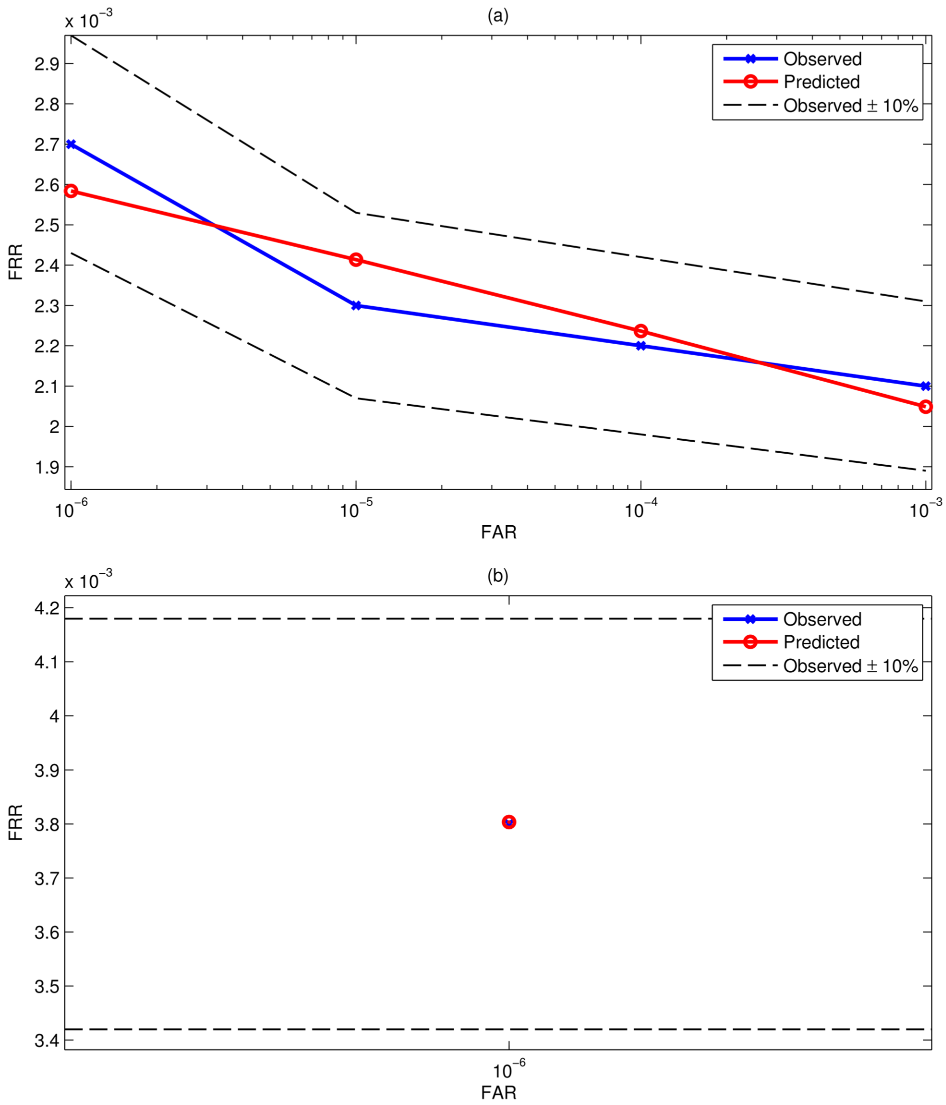

Figure 4. The predicted vs. observed results during the iris parameter estimation procedure for the exclusion scenario. FRR vs. FAR curves for **(a)** the two-iris two-attempt case and **(b)** the two-iris single-attempt case.

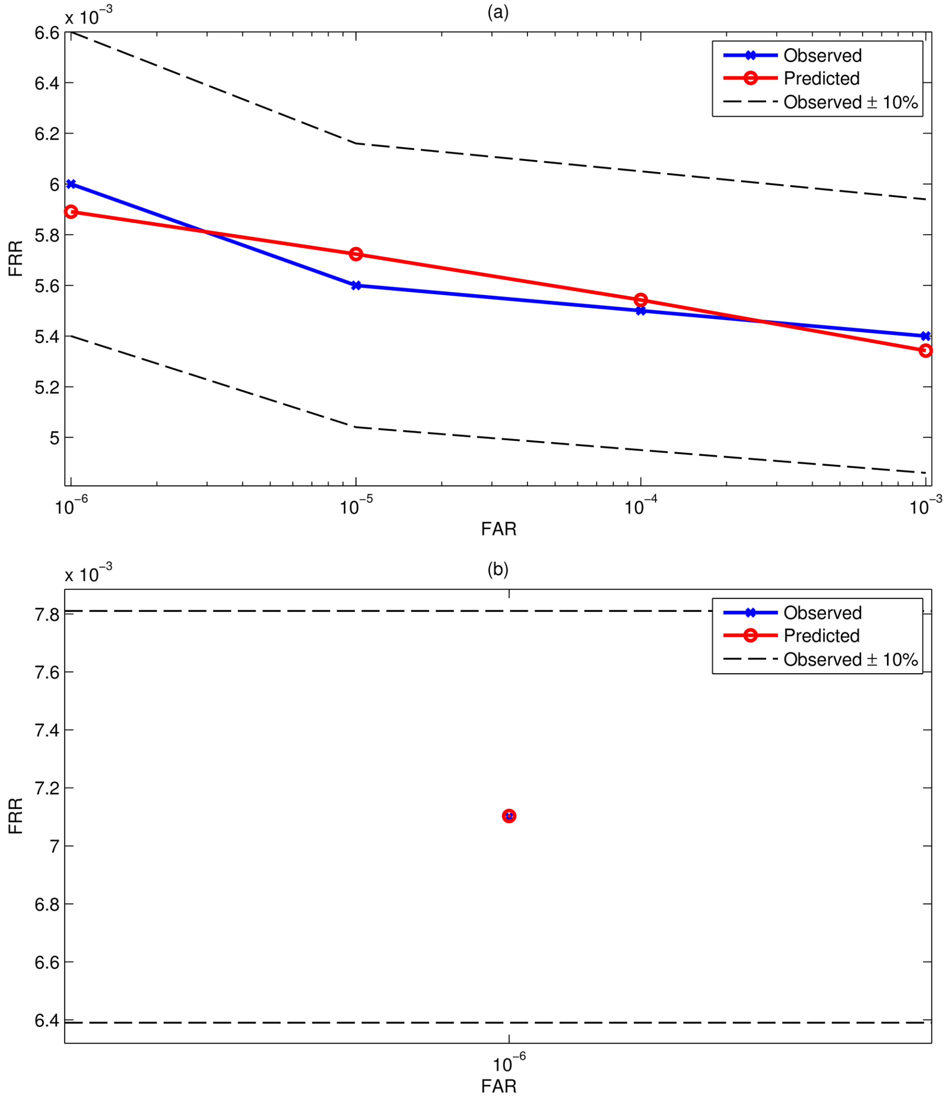

Figure 5. The predicted vs. observed results during the iris parameter estimation procedure for the inclusion scenario. FRR vs. FAR curves for **(a)** the two-iris two-attempt case and **(b)** the two-iris single-attempt case.

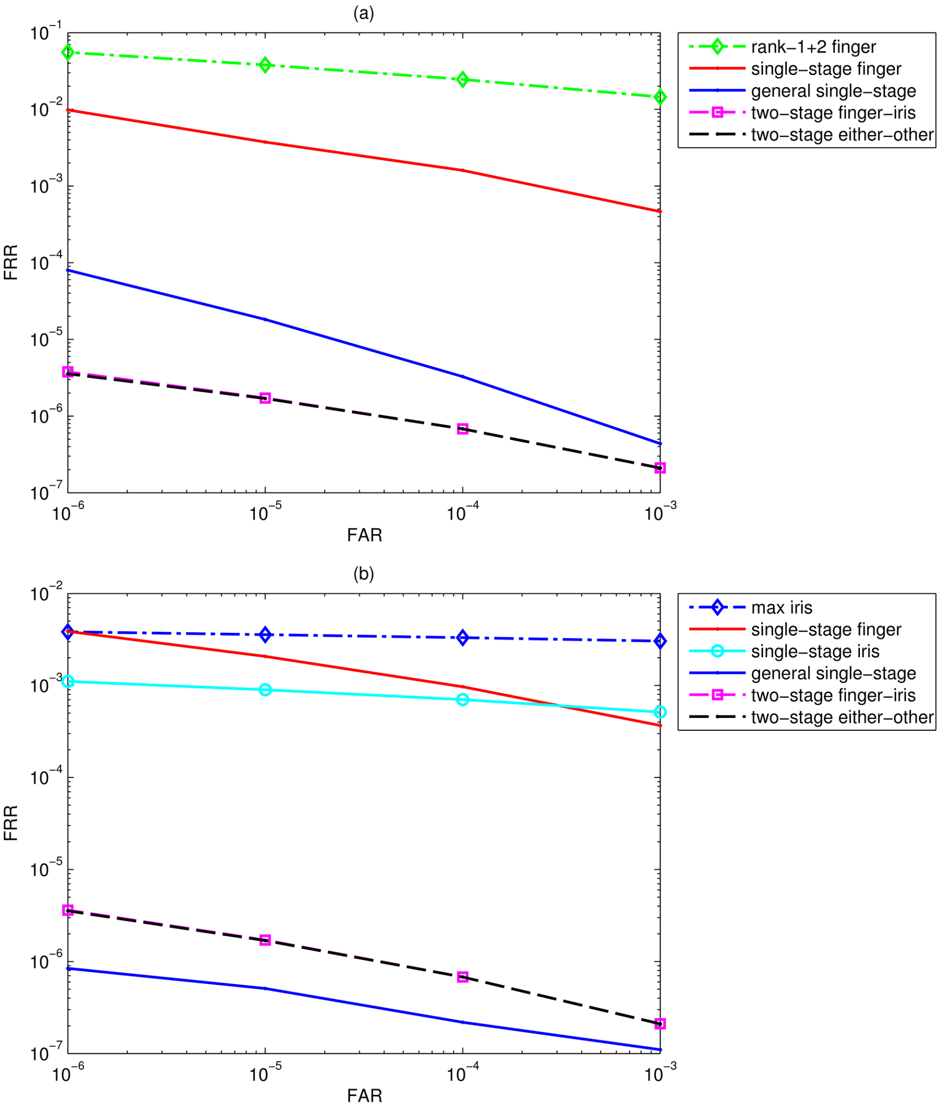

Figure 6. FRR vs. FAR tradeoff curves in the exclusion scenario for mean verification delay values (a) D = 36 sec and (b) D = 43 sec.

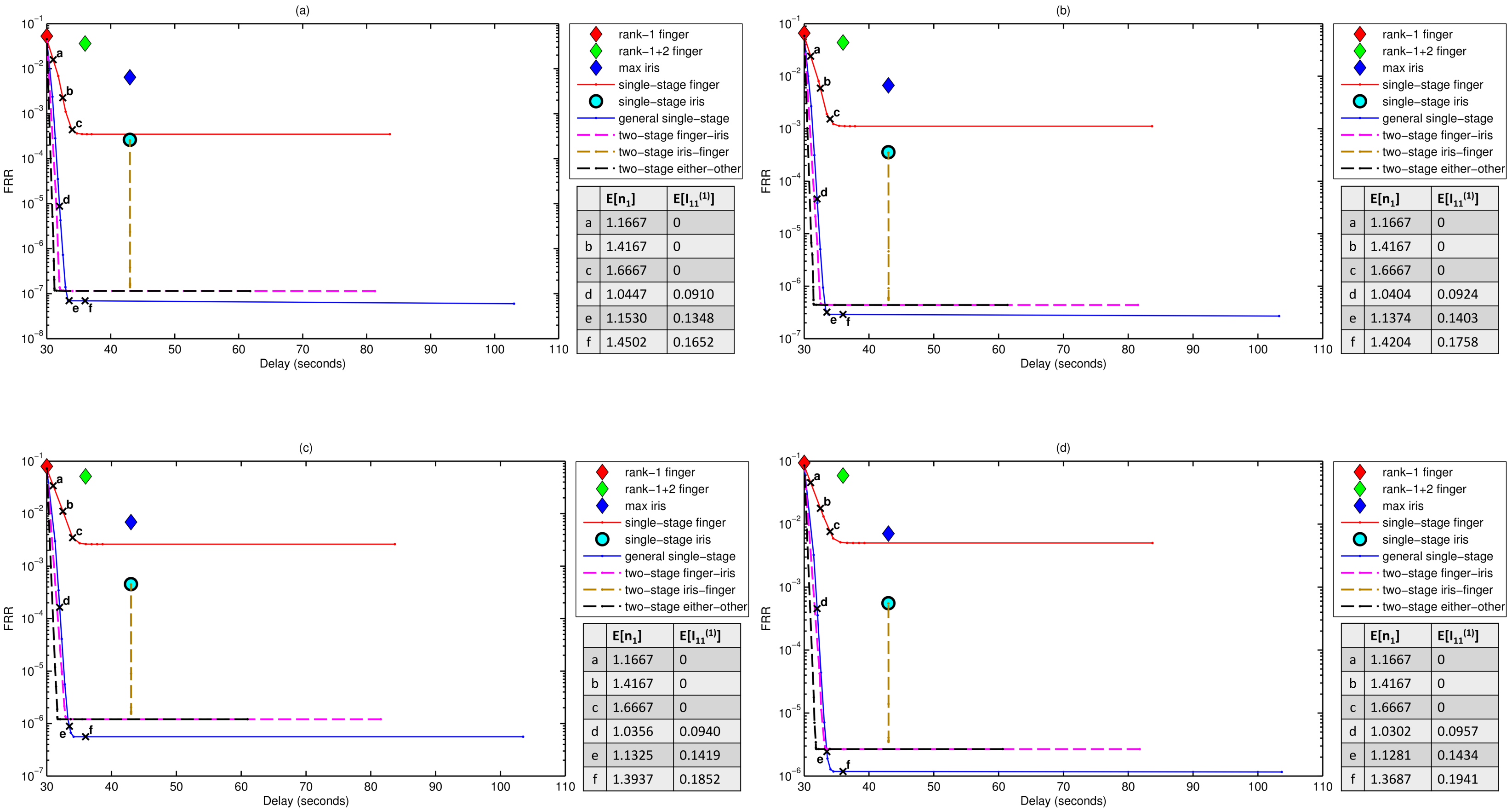

(a)

	$E[n_1]$	$E[I_{11}^{(1)}]$
a	1.1667	0
b	1.4167	0
c	1.6667	0
d	1.0447	0.0910
e	1.1530	0.1348
f	1.4502	0.1652

(b)

	$E[n_1]$	$E[I_{11}^{(1)}]$
a	1.1667	0
b	1.4167	0
c	1.6667	0
d	1.0404	0.0924
e	1.1374	0.1403
f	1.4204	0.1758

(c)

	$E[n_1]$	$E[I_{11}^{(1)}]$
a	1.1667	0
b	1.4167	0
c	1.6667	0
d	1.0356	0.0940
e	1.1325	0.1419
f	1.3937	0.1852

(d)

	$E[n_1]$	$E[I_{11}^{(1)}]$
a	1.1667	0
b	1.4167	0
c	1.6667	0
d	1.0302	0.0957
e	1.1281	0.1434
f	1.3687	0.1941

Figure 7. Results for the three benchmark policies and the six policies in Table 1 of the main text in the inclusion scenario. FRR vs. verification delay tradeoff curves for FRR equals **(a)** 10^{-3}, **(b)** 10^{-4}, **(c)** 10^{-5} and **(d)** 10^{-6}. The mean number of fingers acquired per resident ($E[n_1]$) and the fraction of residents who have their irises acquired ($E[I_{11}^{(1)}]$) are reported for points, a,b,c,x,y,z along two of the tradeoff curves.

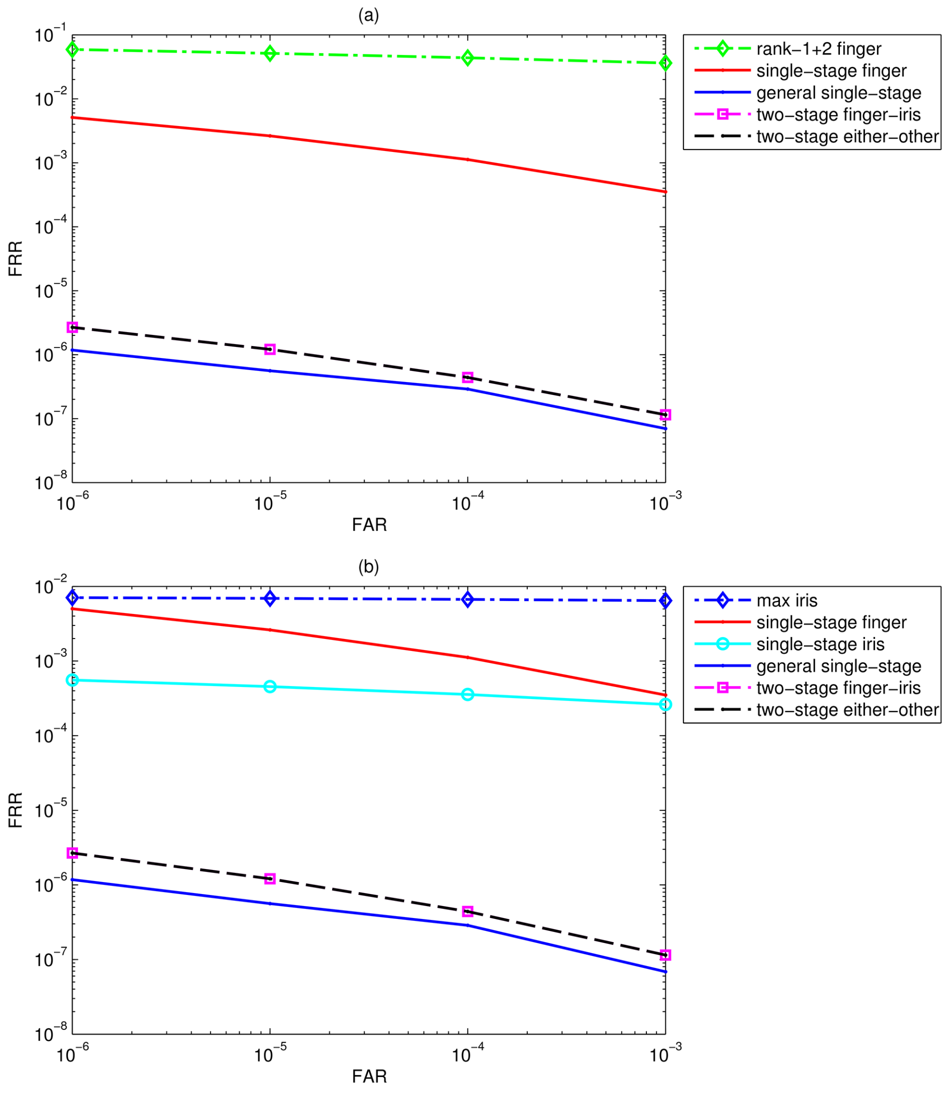

Figure 8. FRR vs. FAR tradeoff curves in the inclusion scenario for mean verification delay values (a) D = 36 sec and (b) D = 43 sec.

Tables

Table 1. Comparison of simulated vs. target FAR values for the exclusion scenario.

Policy	Average Simulated FAR and Average Relative Error When Target FAR is:							
	10^{-3}		10^{-4}		10^{-5}		10^{-6}	
	Average Simulated	Rel Error	Average Simulated	Rel Error	Average Simulated	Rel Error	Average Simulated	Rel Error
Single-stage finger	1.00×10^{-3}	0.1%	1.01×10^{-4}	1.3%	1.00×10^{-5}	0.5%	0.93×10^{-6}	6.66%
Single-stage iris	1.05×10^{-3}	5.4%	0.98×10^{-4}	1.5%	0.87×10^{-5}	13.6%	0.85×10^{-6}	15.0%
General single-stage	1.01×10^{-3}	1.4%	1.02×10^{-4}	1.7%	1.00×10^{-5}	0.1%	0.94×10^{-6}	5.7%
Two-stage iris-finger	1.06×10^{-3}	5.6%	1.00×10^{-4}	0.5%	0.91×10^{-5}	9.2%	0.81×10^{-6}	19.9%
Two-stage finger-iris	1.03×10^{-3}	3.2%	1.02×10^{-4}	2.0%	1.00×10^{-5}	0.33%	0.35×10^{-6}	65.5%
Two-stage either-other	1.04×10^{-3}	3.6%	1.02×10^{-4}	2.1%	0.99×10^{-5}	0.7%	0.34×10^{-6}	65.9%
